# Real-Word Effectiveness of Global COVID-19 Vaccines Against SARS-CoV-2 Variants: A Systematic Review and Meta-Analysis

**DOI:** 10.3389/fmed.2022.820544

**Published:** 2022-05-19

**Authors:** Kai Wang, Lin Wang, Mingzhe Li, Bing Xie, Lu He, Meiyu Wang, Rumin Zhang, Nianzong Hou, Yi Zhang, Fusen Jia

**Affiliations:** ^1^Department of Critical Care Medicine, Zibo Central Hospital, Shandong First Medical University and Shandong Academy of Medical Sciences, Zibo, China; ^2^Independent Researcher, Leeds, United Kingdom; ^3^Department of Hand and Foot Surgery, Zibo Central Hospital, Shandong First Medical University and Shandong Academy of Medical Sciences, Zibo, China; ^4^Hubei University of Medicine, Shiyan, China; ^5^Department of Cardiology, The People's Hospital of Zhangdian District, Zibo, China

**Keywords:** COVID-19, SARS-CoV-2, variant, vaccine, effectiveness, meta-analysis

## Abstract

**Background:**

Currently, promoted vaccinations against SARS-CoV-2 are being given out globally. However, the occurrence of numerous COVID-19 variants has hindered the goal of rapid mitigation of the COVID-19 pandemic by effective mass vaccinations. The real-word effectiveness of the current vaccines against COVID-19 variants has not been assessed by published reviews. Therefore, our study evaluated the overall effectiveness of current vaccines and the differences between the various vaccines and variants.

**Methods:**

PubMed, Embase, Cochrane Library, medRxiv, bioRxiv, and arXiv were searched to screen the eligible studies. The Newcastle–Ottawa scale and the Egger test were applied to estimate the quality of the literature and any publication bias, respectively. The pooled incident rates of different variants after vaccination were estimated by single-arm analysis. Meanwhile, the pooled efficacies of various vaccines against variants were evaluated by two-arm analysis using odds ratios (ORs) and vaccine effectiveness (VE).

**Results:**

A total of 6,118 studies were identified initially and 44 articles were included. We found that the overall incidence of variants post first/second vaccine were 0.07 and 0.03, respectively. The VE of the incidence of variants post first vaccine between the vaccine and the placebo or unvaccinated population was 40% and post second vaccine was 96%, respectively. The sub-single-arm analysis showed a low prevalence rate of COVID-19 variants after specific vaccination with the pooled incidence below 0.10 in most subgroups. Meanwhile, the sub-two-arm analysis indicated that most current vaccines had a good or moderate preventive effect on certain variants considering that the VE in these subgroups was between 66 and 95%, which was broadly in line with the results of the sub-single-arm analysis.

**Conclusion:**

Our meta-analysis shows that the current vaccines that are used globally could prevent COVID-19 infection and restrict the spread of variants to a great extent. We would also support maximizing vaccine uptake with two doses, as the effectiveness of which was more marked compared with one dose. Although the mRNA vaccine was the most effective against variants according to our study, specific vaccines should be taken into account based on the local dominant prevalence of variants.

## Highlights

### What We Already Know About This Topic

COVID-19 has not been fully controlled yet, which has placed a substantial burden on health-care systems and imposed profound negative effects on the economy and society.

A universal SARS-CoV-2 vaccination campaign plays the most critical role in controlling the highly transmissible and pathogenic SARS-CoV-2 infection.

The hope for a rapid mitigation of the COVID-19 pandemic through effective mass vaccination has been dampened by the emergence of numerous SARS-CoV-2 variants worldwide.

The real-word effectiveness of the current COVID-19 vaccines against SARS-CoV-2 variants has not been assessed by a published systematic review and meta-analysis.

### What This Article Tells Us That Is New

Reassuringly, we confirmed the efficacy of vaccines against COVID-19 variants and proved the importance of the booster inoculation after the prime inoculation for the variants, because maximizing vaccine uptake with two doses showed more marked effectiveness than with one dose.

Despite the fact that we found a downward tendency among the effectiveness of vaccines against the newly emerging evolution of SARS-CoV-2 variants in our study, the current vaccines that are used globally could prevent the infection and restrict the transmission of SARS-CoV-2 variants to a great extent.

A two-dose regimen of the mRNA vaccine was the most effective against COVID-19 variants compared to the traditional viral vector vaccine and inactivated vaccine against the placebo group or unvaccinated populations.

The mRNA vaccine was found to be the most effective against variants in our study, however, specific vaccines should be taken into account based on the local dominant prevalence of variants.

## Introduction

In the past 2 years since December 2019, COVID-19, caused by the etiological agent of SARS-CoV-2, has evolved into a global pandemic and a public crisis event, which caused the world to experience a life-changing transition (1, 2). Up to 5:08 pm on 10 March 2022, Central European Time, there were 450,229,635 confirmed cases of COVID-19 and 6,019,085 deaths, according to WHO (3). The considerable morbidity and mortality have brought a heavy economic burden on health-care systems of most countries worldwide and the SARS-CoV-2 virus continues to impose profound negative effects on the economy and society due to measures implemented to control the pandemic. COVID-19 has not been fully controlled yet. Therefore, mask wearing, cleaning our hands, quarantining, ensuring good ventilation indoors, social distancing, avoiding crowds, and therapeutic interventions for treatment are still imperious measures to prevent COVID-19 infection for the foreseeable future. However, an extensive vaccination program for SARS-CoV-2 that shows safety, effectiveness, and cost-efficiency, which is generally thought to be the most promising intervention to eventually end the COVID-19 pandemic by establishing herd immunity among populations, plays the most critical role in controlling the highly transmissible and pathogenic SARS-CoV-2 infection (4, 5).

As a game-changing tool, clinically available COVID-19 vaccines are undergoing unprecedented development by private and public institutions. As of 8 March 2022, 147 vaccine products were in clinical development and another 195 were in the pre-clinical stage (6). Based on traditional and novel technology platforms, these COVID-19 vaccines in clinical development can be divided into at least 10 categories, among which the top five were protein subunit vaccines (48.33%), RNA vaccines (25.17%), viral vector vaccines (non-replicating and replicating, 25, 17%), inactivated vaccines (21.14%), and DNA vaccines (16, 11%) according to the quantity and percentage (7). As of 3 June 2021, WHO proclaimed that some COVID-19 vaccines manufactured by AstraZeneca/Oxford, Pfizer/BioNTech, Moderna, Johnson and Johnson, Sinopharm/Sinovac etc. had reached the required standards of safety and efficacy (8). According to the data of WHO up to now, at least 10 kinds of COVID-19 vaccines, represented by Ad26.COV2.S, BNT162b2, ChAdOx1, mRNA-1273 etc., have been granted WHO Emergency Use Listing (EUL) and prequalification (PQ) (9). A few vaccines in the COVID-19 pandemic have been approved for Emergency Use Authorization (EUA) and/or conditional marketing in several countries, such as Sputnik V, a viral vector vaccine in Russia which was approved on 11 August 2020; BNT162b2, an mRNA vaccine approved in the USA, UK, Canada, and the European Union; an inactivated vaccine produced by Sinopharm in China that was approved on 30 December 2020; and the mRNA-1273 vaccine manufactured by Moderna in the United States (10–12). It is not vaccines that will stop the pandemic, it is vaccination. With the further promotion in the research, development, and application of COVID-19 vaccines by WHO and the regulatory authorities mentioned above, mass SARS-CoV-2 vaccination programs are being widely implemented all over the world. As a result, the global rollout of vaccines offers a glimmer of hope toward terminating COVID-19.

Because SARS-CoV-2 is a class of ribonucleic acid (RNA) coronavirus, its genome changes over time (13). Although most of these changes have little or no influence on the properties of SARS-CoV-2, some may affect the virus' transmission, severity, or how COVID-19 is diagnosed and treated. Since the end of 2020, the occurrence of numerous variants of SARS-CoV-2 has brought a growing threat to global public health. WHO have defined the concepts of variants of interest (VOIs) and variants of concern (VOCs), which could prompt monitoring and research into the variants of global concern (14). Currently, the Centers for Disease Control and Prevention (CDC) are monitoring the four most significant variants (P.1, B.1.1.7, B.1.351B.1.617.2, and B.1.1.529), which may lead to more cases, more hospitalizations, and potentially more deaths than other variants (15). New outbreaks, even in some regions where the virus was initially controlled, and variant strains discovered in multiple countries, either community transmitted or imported, reduced the chance of a rapid termination of the pandemic.

The incidence of variants after vaccination and the effectiveness of vaccines against specific variants of SARS-CoV-2 have always been of interest to WHO, experts, national authorities, institutions, researchers, professionals, common people, and medical workers, however, the conclusions are controversial due to insufficient data. To date, no published systematic reviews or meta-analyses have so far been proved relevant conclusively, therefore, we searched for relevant studies and conducted the present meta-analysis to obtain more precise conclusions on the pooled incidence of variants after vaccination and the vaccine effectiveness (VE) of vaccines against variants compared with placebo. Our systematic review and meta-analysis will offer a few critical guidelines for vaccine selection and promotion, and assist in the current clinical work for preventing and treating COVID-19 variants.

## Materials and Methods

### Search Strategy and Articles Selection

The protocol of our article was according to the PRISMA and MOOSE reporting guidelines (16, 17). We searched PubMed, Cochrane Library, and Embase from 30 December 2019 to 8 March 2022. We also queried medRxiv, bioRxiv, and arXiv for preprints about SARS-CoV-2 variant prevalence after vaccination and the effectiveness of various vaccines against variants. The search terms included (“SARS-CoV-2” OR “COVID-19” OR “2019-nCoV”) AND “vaccin^*^” AND (“varian^*^” OR “mutat^*^”). Key words, subject words, or free words were adjusted according to different requirements of these databases. The references of previously published reviews and articles included in our study were also browsed to acquire more relevant clinical publications.

The records were browsed and all irrelevant papers were removed according to the titles and abstracts by two independent authors from a team of ten. Then, another two authors reviewed the remaining papers to screen potentially eligible ones. Finally, disputes in the process were resolved by discussion of the research group until an agreement was reached for each article.

### Inclusion/Exclusion Criteria

We took into account articles which assessed the prevalence of any type of COVID-19 variant or the efficacy of any type of vaccine against the variants. We evaluated the eligibility criteria of studies using the PICOS (population, intervention/exposure, comparator, outcome, and study) principle (18), which could offer structured approaches to identify relevant data from each paper included. The PICOS principle is as follows: Population—people participating in research associated with vaccines against variants of SARS-CoV-2; intervention/exposure—COVID-19 vaccination; comparator(s)—placebo or unvaccinated population or not applicable due to the single-arm analysis in this study; outcomes—prevalence of SARS-CoV-2 variants after vaccination and/or vaccine effectiveness for prevention or treatment of SARS-CoV-2 variants were evaluated; and study designs—randomized controlled trials, non-randomized studies, comparative trials (C.Ts), cohort studies (C.Ss), observational studies (O.Ss), commentaries, and also letters to the editor were eligible for evaluation, however, editorials, personal opinions, reviews, meta-analyses, conference abstracts, and animal studies were dismissed. We also tried to contact the relevant authors to gain the unpublished data which were required in our study.

The following inclusion criteria were also used to screen all appropriate articles: (1) Articles in English, (2) at least one of the observation indicators was the effectiveness of vaccines against a SARS-CoV-2 variant, (3) studies consisting of at least five patients, and (4) studies with extractable data. The exclusion criteria were as follows: (1) Duplicate studies or study population completely overlapped by other studies, (2) non-accessible full texts, (3) a sample size less than five, (4) studies about pregnant women or neonates, and (5) corresponding outcome parameters that could not be acquired or separated even by contacting the corresponding author.

### Data Extraction

Two relevant authors fetched data from the included articles. The following items were extracted from each article: The first author, publish date, study design, sample size, involved countries or regions, mean or median ages, sex ratio, vaccine name, dose, vaccine type, vaccine developer, comparator, characteristics of vaccine recipients, number of scheduled doses (time of inoculations), study duration, and types of variants. The third author reviewed extracted data at random and disagreements were determined by discussion in the group until a consensus was established.

### Quality Assessment

The Newcastle-Ottawa Scale (NOS) was applied to estimate the quality of the included literature from three points: Patient selection, comparability between groups, and objectivity of results (19). Each aspect received up to 4, 2, and 3 points, respectively and the possible maximum score was 9 points. If the scores were above 4 points, the articles included were considered to have a low or moderate risk of bias. However, studies with points of 4 or fewer were considered to have a high risk of bias and subsequently excluded from our meta-analysis. Two authors independently used NOS to evaluate the quality of the included articles. If they differed in any respect in the quality assessment, other authors offered their opinions to resolve the inconsistencies.

### Statistical Analysis

We used the *I*^2^ (inconsistency indexes) statistical parameter to estimate the heterogeneity between studies included. The value of *I*^2^ assesses the proportion of heterogeneity of all the observed variations and an *I*^2^> 50% is the level of heterogeneity that is attributed to between-study variance. We conducted a fixed-effect model when *I*^2^ <50%, but a random-effect model when *I*^2^ ≥ 50% in the testing of heterogeneity. We performed the Egger test to objectively assess the publication bias of the included studies which were considered to not have publication bias if *p* > 0.05.

The pooled prevalence rate outcomes were evaluated by the incidence rate of a COVID-19 variant after vaccination in single-arm analysis. Meanwhile, the pooled efficacy of vaccines against a SARS-CoV-2 variant was assessed by an odds ratio (OR) and vaccine effectiveness (VE) through comparing the differences of variant cases of SARS-CoV-2 between the vaccination group and placebo or unvaccinated population in two-arm analysis. We calculated the pooled vaccine effectiveness as (1-odds ratio) × 100%, where the odds ratio was equal to the odds of the vaccination population divided by the odds of unvaccinated group.

We also conducted subgroup analyses with delimited and sufficient data based on various vaccines/variants and different doses. If the data of the single-arm analysis were consistent with those of the two-arm analysis in one group, only the two-arm meta-analysis was conducted. All statistical analysis were carried out by R software (version 3.6.1). 95% confidence intervals (CIs) were applied to present the outcomes and a two-tailed *p* <0.05 indicated statistical significance.

## Results

### Literature Selection and Characteristics of Studies Included

**Figure 1:**
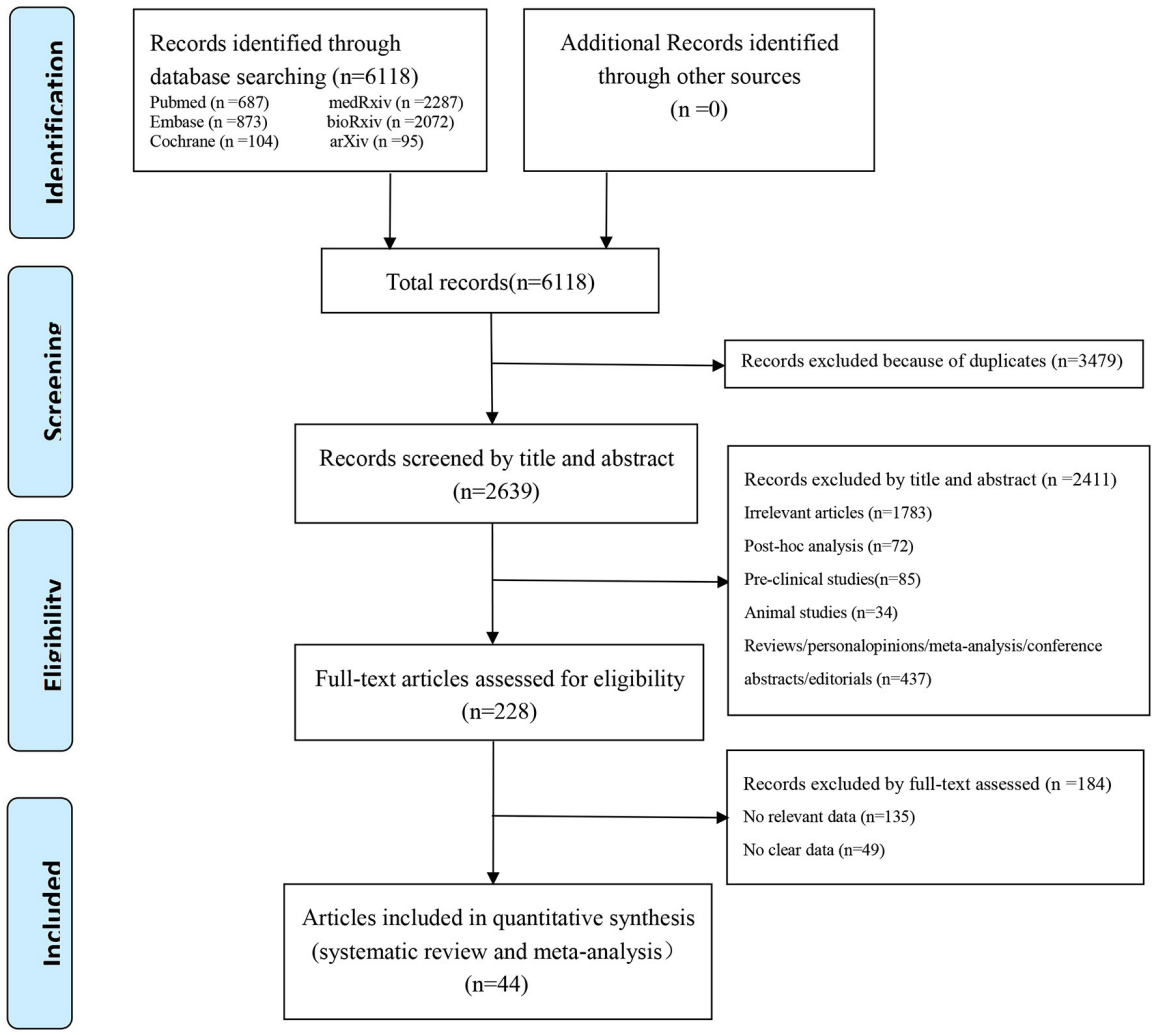
PRISMA flow diagram of the study selection process.

In our preliminary retrieval, we obtained 6,118 studies from PubMed (687), Embase (873), the Cochrane Library (103), medRxiv (2,287), bioRxiv (2072), and arXiv (20). According to the eligible criteria above, 2,639 studies remained after duplicates were initially excluded. Then, 2,411 studies were excluded by title and abstract for the following reasons: Irrelevant articles (*n* = 1,783), post-hoc analysis (*n* = 72), pre-clinical studies (*n* = 85), animal studies (*n* = 34), and reviews/ personal opinions/ meta-analysis/ conference abstracts/ editorials (*n* = 437). After a full-text review, 184 studies without relevant or clear data were further excluded; Consequently, 44 studies (21–64) were finally brought into this systematic review and meta-analysis. The flow diagram summarizing the literature selection process is presented in Figure 1.

Of these studies, 42 were officially published (21–33, 36–64), and two were published on the preprint platform which had not yet been certified by peer review (34, 35). A total of four were blinded, randomized, placebo-controlled trials (21, 22, 42, 55); one was a multicenter, single-blind, randomized phase II/III trial (30); two were multicenter, randomized, placebo-controlled trials (36, 48); 14 were test-negative and case-control trials (23–25, 38, 44, 45, 47, 51, 53, 54, 56, 59, 61, 62); three were matched multicenter or case-control trials (46, 57, 60); two were cross-sectional trials (26, 28); one was a prospective cohort trial (27); three were case-control trials (32, 33, 37); and 14 were observational cohort trials (29, 31, 34, 35, 39–41, 43, 49, 50, 52, 58, 63, 64). These included studies contained eight kinds of COVID-19 vaccines: ChAdOx1 (21, 25, 30, 36, 37, 40, 46, 47, 54, 63), ChAdOx1-S (49), NVX-CoV2373 (22, 42, 55), CoronaVac (23, 35, 45, 56), BBV152 (51), BNT162b2 (24–29, 31–34, 37–41, 43, 44, 48, 50, 52, 57–64), mRNA-1273 (38, 40, 48, 52, 53, 57), and JNJ-78436735 (52). All of which could be classified into viral vector vaccines, subunit vaccines, inactivated vaccines, and mRNA vaccines, respectively, on the basis of different technology platforms. The variants involved in the studies included B.1.1.7 (Alpha), B.1.351 (Beta), P.1/P.1.1/P.1.2/B.1.1.28 (Gamma), B.1.617.2 (Delta), B.1.427/B.1.429 (Epsilon), P.2 (Zeta), B.1.525 (Eta), B.1.526/B.1.526.1/B.1.526.2 (Iota), B.1.617.1 (Kappa), B.1.621/B.1.621.1 (Mu), B.1.1.529/BA (Omicron), R.1, B.1, and B.1.1.33. Among them, four studies were conducted in South Africa (21, 22, 36, 62), seven in the USA (28, 32, 34, 48, 52, 57, 61), seven in the UK (27, 30, 40, 42, 49, 53, 63), five in Brazil (23, 35, 46, 54, 56), four in Israel (29, 33, 60, 64), four in Qatar (24, 38, 44, 59), three in India (25, 47, 51), three in Italy (26, 39, 41), three in France (31, 37, 50), 1 in China (45), 1 in Korea (43), 1 in the USA and Mexico (55), and 1 in French Guiana (58). The baseline characteristics of the literature are presented in Supplementary Table 1.

### Quality Assessment and Publication Bias

All the 44 studies were quality-assessed based on NOS. Among them, 18 studies had nine points (21, 22, 30, 37, 38, 40, 42, 44, 47, 48, 51–56, 60, 61), five had 8 points (31, 33, 36, 46, 59), seven had 7 points (24, 25, 32, 39, 45, 57, 62), 10 had 6 points (23, 26, 28, 34, 41, 43, 50, 58, 63, 64), and four had 5 points (27, 29, 35, 49). There were relatively high risks of bias in the literature of Hall et al. (27), Haas et al. (29), de Faria et al. (35), and Williams et al. (49) in which “selection of the non-exposed patients” and “comparability between groups” were the two most important deduction items. The summary and figures of risk bias in the eligible studies are shown in Table 1.

**Table 1:** Quality evaluation of eligible studies based on the Newcastle-Ottawa Scale.

**References**	**Selection**				**Comparability**	**Outcomes**			**Total** **scores**
	**Representativeness of the exposed cohort**	**Selection of the non-exposed cohort**	**Ascertainment of exposure**	**Demonstration that outcome of interest was not present at start of study**	**Comparability of cohorts on the basis of the design or analysis**	**Assessment of outcome**	**Was follow-up long enough for outcomes to occur?**	**Adequacy of follow-up of cohorts**	
Madhi et al. (21)	1	1	1	1	2	1	1	1	9
Shinde et al. (22)	1	1	1	1	2	1	1	1	9
Hitchings et al. (23)	1	Nil	1	Nil	1	1	1	1	6
Abu-Raddad et al. (24)	1	1	1	Nil	1	1	1	1	7
Lopez Bernal et al. (25)	1	1	1	Nil	1	1	1	1	7
Sansone et al. (26)	1	Nil	1	Nil	1	1	1	1	6
Hall et al., (27)	1	Nil	1	Nil	Nil	1	1	1	5
Jacobson et al., (28)	1	Nil	1	1	Nil	1	1	1	6
Haas et al. (29)	1	Nil	Nil	1	Nil	1	1	1	5
Emary et al. (30)	1	1	1	1	2	1	1	1	9
Bailly et al. (31)	1	1	1	1	1	1	1	1	8
Cavanaugh et al. (32)	1	1	1	1	1	1	Nil	1	7
Kustin et al. (33)	1	1	Nil	1	2	1	1	1	8
Magalis et al. (34)	1	Nil	1	1	Nil	1	1	1	6
de Faria et al. (35)	1	Nil	Nil	1	Nil	1	1	1	5
Irfan et al. (36)	1	1	1	1	2	1	Nil	1	8
Grant et al. (37)	1	1	1	1	2	1	1	1	9
Tang et al. (38)	1	1	1	1	2	1	1	1	9
Rovida et al. (39)	1	1	Nil	1	1	1	1	1	7
Pouwels et al. (40)	1	1	1	1	2	1	1	1	9
Trunfio et al. (41)	1	1	Nil	1	1	1	1	1	6
Heath et al. (42)	1	1	1	1	2	1	1	1	9
Yi et al. (43)	1	Nil	1	1	Nil	1	1	1	6
Chemaitelly et al. (44)	1	1	1	1	2	1	1	1	9
Li et al. (45)	1	1	1	1	1	1	Nil	1	7
Clemens et al. (46)	1	1	1	1	1	1	1	1	8
Thiruvengadam et al. (47)	1	1	1	1	2	1	1	1	9
Tenforde et al. (48)	1	1	1	1	2	1	1	1	9
Williams et al. (49)	1	Nil	Nil	1	Nil	1	1	1	5
Lefèvre et al. (50)	1	Nil	1	1	Nil	1	1	1	6
Desai et al. (51)	1	1	1	1	2	1	1	1	9
Duerr et al. (52)	1	1	1	1	2	1	1	1	9
Bruxvoort et al. (53)	1	1	1	1	2	1	1	1	9
Hitchings et al. (54)	1	1	1	1	2	1	1	1	9
Dunkle et al. (55)	1	1	1	1	2	1	1	1	9
Ranzani et al. (56)	1	1	1	1	2	1	1	1	9
Dickerman et al. (57)	1	1	1	1	Nil	1	1	1	7
Vignier et al. (58)	1	Nil	1	1	Nil	1	1	1	6
Abu-Raddad et al. (59)	1	1	1	1	1	1	1	1	8
Reis et al. (60)	1	1	1	1	2	1	1	1	9
Olson et al. (61)	1	1	1	1	2	1	1	1	9
Collie et al. (62)	1	1	1	1	1	1	Nil	1	7
Eyre et al. (63)	1	Nil	1	1	1	1	Nil	1	6
Mor et al. (64)	1	Nil	1	1	1	1	Nil	1	6

The *p* values derived from Egger's test indicated the inexistence of publication bias in most meta-analyses. High probabilities of publication bias existed in the following subgroup meta-analyses: Incidence of variants post second vaccine, incidence of the B.1.1.7 (Alpha) variant post first vaccine, incidence of the B.1.1.7 (Alpha) variant post second vaccine, incidence of the B.1.1.7 (Alpha) variant post first mRNA vaccine, incidence of the B.1.351 (Beta) variant post second vaccine, incidence of the B.1.351 (Beta) variant post second mRNA vaccine, incidence of the B.1.617.2 (Delta) variant post first vaccine, incidence of the B.1.617.2 (Delta) variant post first viral vector vaccine, efficacy of vaccines against variants post second dose, efficacy of vaccines against the B.1.1.7 (Alpha) variant post second dose, and efficacy of an mRNA vaccine against the B.1.1.7 (Alpha) variant post second dose. The publication bias of these sub-analyses (incidence of variants post second protein subunit vaccine, incidence of variants post second inactivated vaccine, incidence of the B.1.1.7 (Alpha) variant post second protein subunit vaccine, incidence of the B.1.351 (Beta) variant post second viral vector vaccine, incidence of the B.1.351 (Beta) variant post second protein subunit vaccine, incidence of the P.1 (Gamma) variant post second viral vector vaccine, incidence of the B.1.427 (Epsilon) variant post second mRNA vaccine, incidence of the P.2 (Zeta) variant post second vaccine, incidence of the B.1.526 (Iota) variant post second vaccine, incidence of the B.1.526 (Iota) variant post second mRNA vaccine, efficacy of a subunit vaccine against the B.1.1.7 (Alpha) variant post second dose, efficacy of a viral vector vaccine against the P.1 (Gamma) variant post second dose, efficacy of vaccines against the B.1.427 (Epsilon) variant post first dose, efficacy of an mRNA vaccine against the B.1.427 (Epsilon) variant post first dose, efficacy of an mRNA vaccine against the B.1.427 (Epsilon) variant post second dose, efficacy of vaccines against the P.2 (Zeta) variant post second dose, and efficacy of mRNA vaccines against the B.1.526 (Iota) variant post second dose) could not be evaluated for fewer studies were included in each subgroup. The results of the Egger's test are summarized in Supplementary Table 2.

### Meta-Analyses Results

There was substantial heterogeneity (*I*^2^≥ 50%, *p* ≤ 0.05) in most of the groups, hence, the random effects model was conducted in most of these meta-analyses. However, the fixed effects models were used in these analyses as follows: Incidence of the B.1.351 (Beta) variant post second viral vector vaccine, incidence of the B.1.427 (Epsilon) variant post second mRNA vaccine, incidence of the P.2 (Zeta) variant post second vaccine, efficacy of a subunit vaccine against the B.1.1.7 (Alpha) variant post second dose, efficacy of vaccines against the B.1.351 (Beta) variant post first dose, efficacy of an mRNA vaccine against the B.1.351 (Beta) variant post first dose, efficacy of an mRNA vaccine against the P.1 (Gamma) variant post first dose, efficacy of vaccines against the B.1.427 (Epsilon) variant post first dose, efficacy of vaccines against the B.1.427 (Epsilon) variant post second dose, efficacy of an mRNA vaccine against the B.1.427 (Epsilon) variant post first dose, efficacy of an mRNA vaccine against the B.1.427 (Epsilon) variant post second dose, and efficacy of vaccines against the P.2 (Zeta) variant post second dose. The *I*^2^ and *p* values of which were all <50% and >0.05, respectively. The results of the heterogeneity test are shown in Supplementary Table 2.

#### The Pooled Incident Rates of COVID-19 Variants After Vaccination

**Figure 2:**
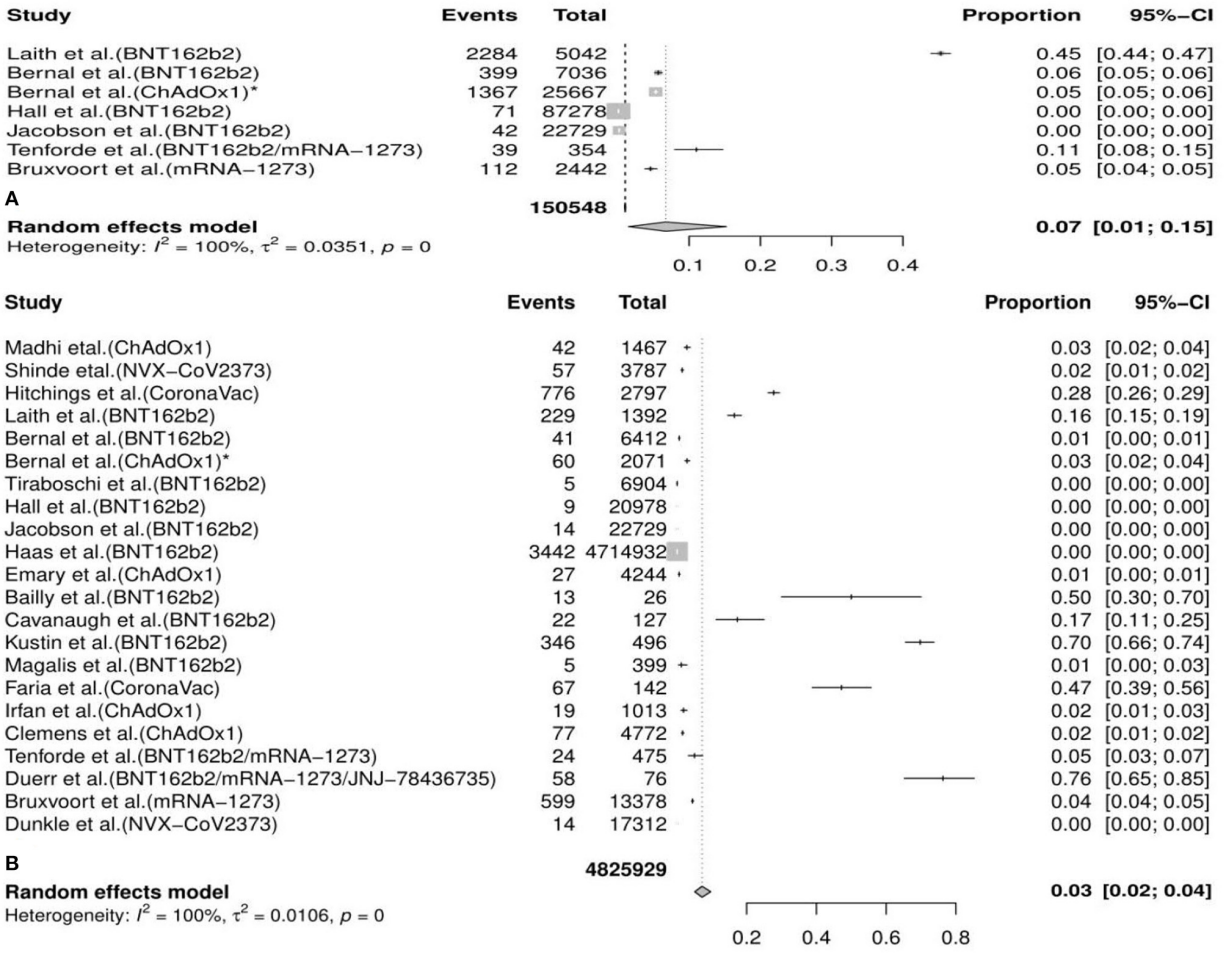
Meta-analysis for the overall incidence of various COVID-19 variants post first vaccine **(A)** and post second vaccine **(B)**.* indicates the second arm in the study of Bernal et al. CI, confidence interval.

In the meta-analysis, we found that the overall incidence of variants post first vaccine was 0.07 [95%CI: 0.01, 0.15] and post second vaccine was 0.03 [95%CI: 0.02, 0.04]. According to the types of vaccines/variants and the first/second dose, the subgroup meta-analyses were divided into 37 categories. The results of subgroup analyses (incidence of variants post first vaccine, incidence of variants post second vaccine, incidence of variants post first mRNA vaccine, incidence of variants post second mRNA vaccine, incidence of variants post second viral vector vaccine, etc.) revealed a significant protective effect of the vaccines against COVID-19 variants with the fact that the pooled incident rates were below 0.10 (pooled incidence=0.07, 95%CI: 0.01, 0.15; 0.02, 95%CI: 0.00, 0.13; 0.07, 95%CI: 0.00, 0.21; 0.06, 95%CI: 0.04, 0.09; 0.02, 95%CI: 0.01, 0.02, etc., respectively). However, the results of the remaining seven subgroup analyses (incidence of variants post second inactivated vaccine, incidence of the B.1.1.7 (Alpha) variant post first mRNA vaccine, incidence of the B.1.351 (Beta) variant post first vaccine, incidence of the B.1.351 (Beta) variant post first mRNA vaccine, incidence of the P.1 (Gamma) variant post first vaccine, incidence of the B.1.617.2 (Delta) variant post first vaccine, and incidence of the B.1.526 (Iota) variant post second mRNA vaccine) presented a moderate protective effect of the vaccines against COVID-19 variants considering that the pooled incident rates were over 0.10 (pooled incidence= 0.37, 95%CI:0.19, 0.57; 0.16, 95%CI: 0.15,0.16; 0.35, 95%CI: 0.04, 0.66; 0.30, 95%CI: 0.14, 0.50; 0.36, 95%CI: 0.26, 0.46; 0.14, 95%CI: 0.11, 0.18; 0.12, 95%CI: 0.01, 0.59, respectively). The details of the meta-analysis results are shown in Table 2, Figure 2, Supplementary Table 2, and Supplementary Figure 1.

**Table 2:** Results of the meta-analysis.

**Variants** **vaccines**	**Overall** **Variants**	**B.1.1.7** **(Alpha)** **variant**	**B.1.351** **(Beta)** **variant**	**P.1** **(Gamma)** **variant**	**B.1.617.2** **(Delta)** **variant**	**B.1.427** **(Epsilon)** **variant**	**P.2** **(Zeta)** **variant**	**B.1.526** **(Iota)** **variant**
Overall vaccines	0.07 [0.01; 0.15]^*^	0.07 [0.05; 0.10]^*^	0.35 [0.04; 0.66]^*^	0.14 [0.02; 0.34]^*^	0.14 [0.11; 0.18]^*^	0.00 [0.00; 0.04]^*^	NA	NA
	0.03 [0.02; 0.04]^†^	0.04 [0.03; 0.05]^†^	0.09 [0.03; 0.19]^†^	0.09 [0.06; 0.16]^†^	0.08 [0.05; 0.11]^†^	0.00 [0.00; 0.01]^†^	0.00 [0.00; 0.22]^†^	0.01 [0.00; 0.80]^†^
	0.40 [0.38, 0.42]^§^	0.66 [0.36; 0.82]^§^	0.16 [0.11; 0.20]^§^	0.35 [0.05; 0.56]^§^	0.38 [0.15; 0.55]^§^	0.78 [0.54; 0.90]^§^	NA	NA
	0.96 [0.93; 0.98]^¶^	0.90 [0.79; 0.95]^¶^	0.42 [0.00; 0.70]^¶^	0.61 [0.50; 0.70]^¶^	0.68 [0.57; 0.76]^¶^	0.95 [0.87; 0.98]^¶^	0.69 [0.55; 0.78]^¶^	0.71 [0.00; 0.96]^¶^
mRNA vaccine (BNT162b2/mRNA-1273/JNJ-78436735)	0.07 [0.00; 0.21]^*^	0.16 [0.15; 0.16]^*^	0.30 [0.14; 0.50]^*^	0.09 [0.00; 0.26]^*^	0.09 [0.03; 0.18]^*^	0.00 [0.00; 0.04]^*^	NA	NA
	0.06 [0.04; 0.09]^†^	0.09 [0.06; 0.14]^†^	0.10 [0.03; 0.22]^†^	0.06 [0.01; 0.16]^†^	0.09 [0.05; 0.14]^†^	0.00 [0.00; 0.04]^†^	NA	0.12 [0.01; 0.59]^†^
	0.35 [0.13; 0.51]^§^	0.64 [0.00; 0.87]^§^	0.16 [0.11; 0.20]^§^	0.57 [0.05; 0.81]^§^	NA	0.78 [0.54; 0.90]^§^	NA	NA
	0.85 [0.28; 0.97]^¶^	0.89 [0.74; 0.95]^¶^	0.40 [0.00; 0.72]^¶^	0.68 [0.00; 0.95]^¶^	0.74 [0.62; 0.82]^¶^	0.95 [0.86; 0.98]^¶^	NA	0.62 [0.00; 0.98]^¶^
Viral vector vaccine (ChAdOx1/ChAdOx1-S)	NA	0.10 [0.07; 0.14]^*^	NA	NA	0.06 [0.02; 0.14]^*^	NA	NA	NA
	0.02 [0.01; 0.02]^†^	0.00 [0.00; 0.01]^†^	0.02 [0.02; 0.03]^†^	0.05 [0.00; 0.67]^†^	0.03 [0.00; 0.09]^†^	NA	NA	NA
	NA	NA	NA	NA	0.50 [0.35; 0.61]^§^	NA	NA	NA
	0.66 [0.51; 0.77]^¶^	0.94 [0.30; 1.00]^¶^	NA	0.57 [0.25; 0.75]^¶^	0.62 [0.31; 0.79]^¶^	NA	NA	NA
Protein subunit vaccine (NVX-CoV2373)	NA	NA	NA	NA	NA	NA	NA	NA
	0.03 [0.00; 0.03]^†^	0.00 [0.00; 0.00]^†^	0.00 [0.00; 0.02]^†^	NA	NA	NA	NA	NA
	NA	NA	NA	NA	NA	NA	NA	NA
	NA	0.89 [0.80; 0.94]^¶^	NA	NA	NA	NA	NA	NA
Inactivated vaccine (CoronaVac/BBV152)	NA	NA	NA	NA	NA	NA	NA	NA
	0.37 [0.19; 0.57]^†^	NA	NA	0.36 [0.26; 0.46]^†^	NA	NA	NA	NA
	NA	NA	NA	NA	NA	NA	NA	NA
	NA	NA	NA	NA	NA	NA	NA	NA

* *Incidence of variants post first vaccine (95% CI)*.

† *Incidence of variants post second vaccine (95% CI)*.

§ *Vaccine effectiveness post first vaccine (95% CI)*.

¶ *Vaccine effectiveness post second vaccine (95% CI)*.

*NA, not applicable; CI, confidence interval*.

#### The Pooled Efficacy of Vaccines Against SARS-CoV-2 Variants

**Figure 3:**
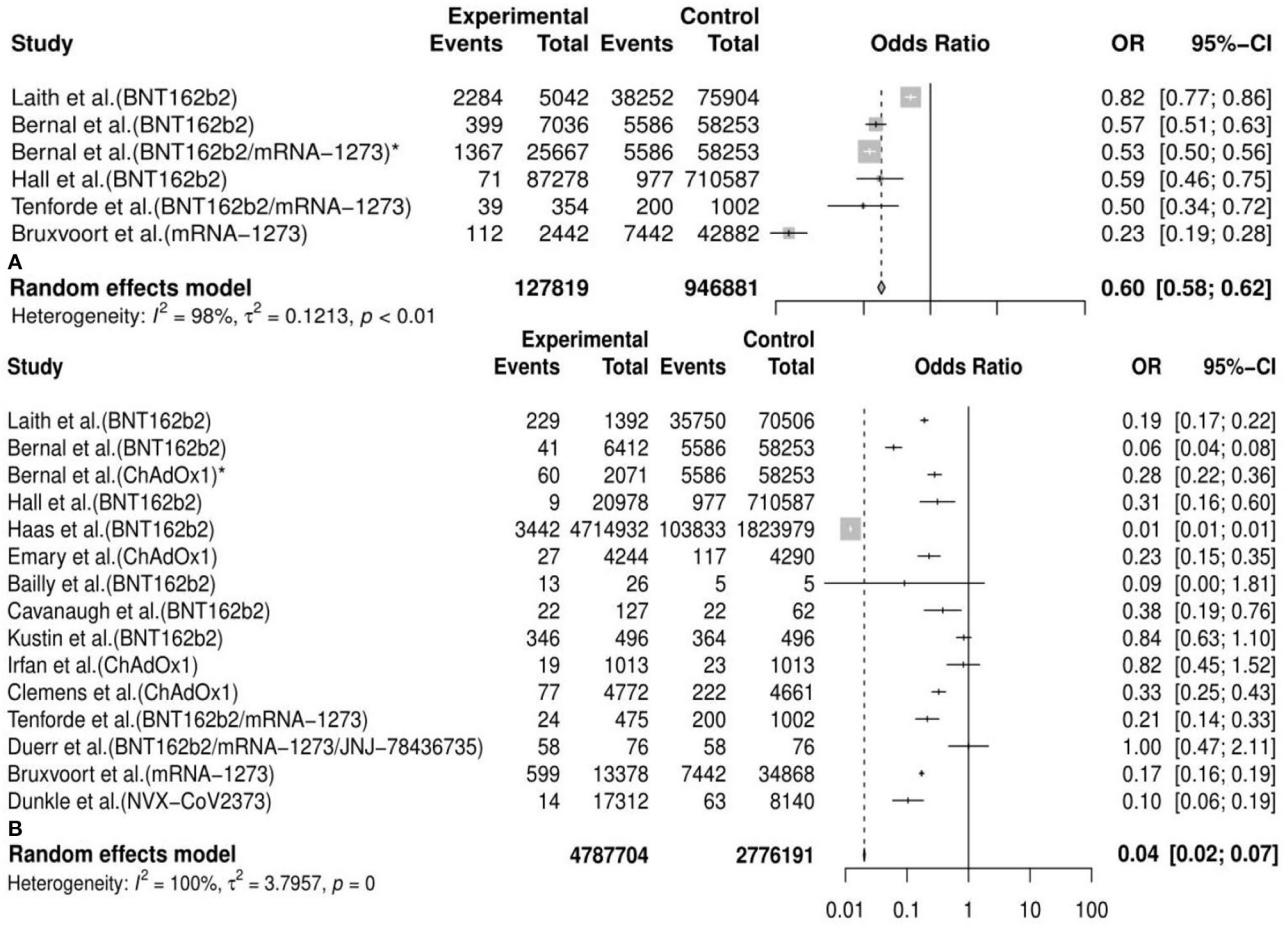
Meta-analysis for the overall efficacy of vaccines against COVID-19 variants post first vaccine **(A)** and post second vaccine **(B)**. * indicates the second arm in the study of Bernal et al. CI. confidence interval; OR, odds ratio.

Generally, we observed that the vaccine effectiveness (VE) of incidence of variants post first vaccine between the vaccine and the placebo or unvaccinated population was 0.40 [95%CI: 0.38, 0.42] and post second vaccine was 0.96 [95%CI: 0.93, 0.98] in the meta-analysis. We also conducted 30 subgroup meta-analyses according to the classifications mentioned above. The results of 20 subgroup (efficacy of an mRNA vaccine against variants post second dose, efficacy of vaccines against the B.1.1.7 (Alpha) variant post second dose, efficacy of an mRNA vaccine against the B.1.1.7 (Alpha) variant post second dose, efficacy of a subunit vaccine against the B.1.1.7 (Alpha) variant post second dose, efficacy of a viral vector vaccine against the B.1.1.7 (Alpha) variant post second dose, etc.) analyses implied that some vaccines had a better preventive and therapeutic effect on certain variants among those cases following the vaccination, placebo, or unvaccinated populations, considering that the VE in these subgroups was between 60% and 95% (VE= 0.85, 95%CI: 0.28, 0.97; 0.90, 95%CI: 0.79, 0.95; 0.89, 95%CI: 0.74, 0.95; 0.89, 95%CI: 0.80, 0.94; 0.94, 95%CI: 0.30, 1.00, etc., respectively). Besides, the remaining results of another 10 subgroup analyses (efficacy of vaccines against the B.1.351 (Beta) variant post first dose, efficacy of vaccines against the P.1 (Gamma) variant post first dose, efficacy of an mRNA vaccine against variants post first dose, efficacy of vaccines against the B.1.351 (Beta) variant post second dose, efficacy of a viral vector vaccine against the P.1 (Gamma) variant post second dose, efficacy of a viral vector vaccine against the P.1 (Gamma) variant post second dose, etc.) showed a passable protective effect of some vaccines against certain COVID-19 variants in view that the VE in these subgroups was between 16% and 57% (VE=0.16, 95%CI: 0.11, 0.20; 0.35, 95%CI: 0.05, 0.56; 0.35, 95%CI: 0.13, 0.51; 0.42, 95%CI: 0.00, 0.70; 0.57, 95%CI: 0.25, 0.75, etc., respectively). All details of the meta-analysis results are shown in Table 2, Figure 3, Supplementary Figure 2, and Supplementary Table 2.

## Discussion

The emergence of COVID-19 variants and their mutations, especially those identified in the UK (B1.1.7, Alpha), South Africa (B1.351, Beta; B.1.1.529, Omicron), Brazil (P.1, Gamma; P.2, Zeta), India (B.1.617.2, Delta; B.1.617.1, Kappa), the USA (B.1.427/B.1.429, Epsilon; B.1.525, Eta; B.1.526, Iota), the Philippines and Japan (P.3, Theta), the South American region (C.37, Lambda), and Columbia (B.1.621, Mu), highlight the conspicuous abilities of SARS-CoV-2 to rapidly generate new gene variants, which have raised concerns about the possibility that these mutants may evade vaccines (65, 66). At present, the lack of understanding of pathogenic and immunologic mechanisms and duration of immunity of vaccines are still the main challenges against combatting the variants of SARS-CoV-2 (67). Although these variants have been demonstrated to dramatically reduce the neutralization by specific antibodies or sera elicited by vaccination against SARS-CoV-2 in several studies recently (68–71), multiple works have verified that vaccine-induced human antibodies could protect against emerging SARS-CoV-2 variants and mitigate the vaccine resistance caused by the current VOCs (72–74). Indeed, the process of neutralizing vaccine-induced antibodies *in vivo* could not mirror the complicated interaction and cross-talk between SARS-CoV-2 and humans *in vivo*. Furthermore, the results of real-world clinical trials were controversial in terms of the conclusions about the effectiveness of vaccines against variants (21–23, 30, 31, 34, 36). On account of the fact that the current vaccines' efficacy has not yet been comprehensively discussed, many unsubstantiated claims have been made by popular media and politicians, which often negatively affect real-world mass vaccination campaigns. Therefore, we mainly focused on existing and available studies and strived to provide a systematic and comprehensive review regarding the incidence of variants after vaccination and the efficacy of vaccines against variants if possible in the meta-analysis.

Based on the consequences of the meta-analysis, we found that the overall incidence of variants post first vaccine was 0.07 [95%CI: 0.01, 0.15] and post second vaccine was 0.03 (95%CI: 0.02, 0.04). The definition of “incidence” in our study indicated the number of cases with any specific variants but other variants detected in the same patients were not repeatedly included. Although SARS-CoV-2 mutates all the time, the newly emerging variants could be predicted and probably be identified by all sequenced genomes. In a neutralizing trial about human monoclonal antibodies induced by vaccines against variants of SARS-CoV-2, Schmitz et al. reported that the escaped variants accounted for <0.008% of sequenced clinically isolated viruses through all publicly available SARS-CoV-2 genome sequences (72). Currently, breakthrough infections in partial or full vaccination populations have been reported but the initial findings indicated that these cases (PVSCs) were uncommon (75, 76). In a cross-sectional study conducted in northern California, Jacobson et al. reported that the incidence of COVID-19 after vaccination was about 0.83% (189/22,729) and the incidence of VOCs (B.1.427 and B.1.429) was only about 0.19% (43/22,729) (28). Our results basically aligned with the conclusions in real-word clinical trials (22, 25, 27, 29, 30). Hence, the estimation in the meta-analysis for the incidence of variants post vaccination was reliable and the relatively low overall incidence confirmed the efficacy of vaccines against COVID-19 variants. Based on the two-arm meta-analysis, the overall vaccine effectiveness (VE) against variants post first vaccine was 0.40 [95%CI: 0.38, 0.42] and post second vaccine was 0.96 [95%CI: 0.93, 0.98]. Regarding the effectiveness of current vaccines against COVID-19, several reviews and meta-analyses have been published, which did not make a distinction between wild-type SARS-CoV-2 and variants (77–79). In a meta-analysis about vaccines of COVID-19 in phase III trials, Cheng et al. concluded that overall vaccines currently had a good protective effect against COVID-19 among patients after vaccination with an efficiency of 83% (95%CI: 0.68–0.91) (77). In another meta-analysis of randomized clinical trials, Pormohammad et al. found that the pooled efficiency of vaccines based on different technical platforms was from 80.2 to 94.6% (79). Therefore, we have reason to think that there are not many differences in vaccines' ability to elicit immune responses when they confront COVID-19 and its variants.

Most vaccines currently in use require two doses and this two-step vaccination process is called “prime-boost”. Generally, individuals were deemed to be fully vaccinated 14 days or longer after acquiring their second dose in a two-vaccination procedure with a mean interval time over 2 weeks (75, 76, 80). Whereas, single-dose vaccination is more feasible and contributes to a higher acceptance of vaccination for the mass population in the real world (80, 81). Both the pressure from the vaccine supply chain and the vaccine hesitation in the public caused by the concern over safety inevitably impede full vaccination (82, 83). Most studies showed that two-dose vaccination had better immunogenicity and efficacy compared with a single-dose regimen for most vaccines. Kow et al. found that the pooled protective rate of the BNT162b2 mRNA vaccine after the first dose was 82%, which was lower than the efficacy of 95% after the second dose (78). Pormohammad et al. concluded that there were no differences among the effectiveness of some COVID-19 vaccines after the first and second dose, such as adenovirus-vectored vaccines (97.6 vs. 99.9%), inactivated vaccines (91.3 vs. 94%), and pro-subunit vaccines (87.3 vs. 95.6%) (79). Nevertheless, they also admitted that this efficacy was estimated according to the amount of neutralizing antibodies but not the incidence rate, which could not substitute the protection rate in the real world. However, the author emphasized that the introduction of the second dose of vaccine could produce more reliable results, because the variation in the efficacy after the second dose was more notable (79). Saad-Roy et al. built a model of immuno-epidemiology and explored whether a one-dose vaccine policy generally protected individuals against COVID-19 in the short run but that partial vaccination inevitably promoted antigenic evolution (84). Our results showed that the vaccines reduced the incidence rate of variants by 71.4% and increased the efficacy against the variants of concern by 140% after the second dose relative to after the first dose, which again proved the importance of the booster inoculation after the prime inoculation, especially for the COVID-19 variants. The theory we suspected may be that if the vaccines train the immune system to recognize a virus repeatedly, then, the immune response might become more durable and broader which could help to screen for SARS-COV-2 with slightly less virulent variants. Moreover, Jacobson et al. reported that the majority of breakthrough cases occurred <2 weeks after the first/second dose of vaccine and emphasized that excellent vaccine effectiveness usually appeared > 2 weeks after the second vaccine (28). Therefore, we suggest that the public should be vaccinated as soon as possible with two doses to build up full immunity against variants of SARS-CoV-2 and highlight the necessity to build strict preventive measures until herd immunity is established after 14 days post the second dose.

When the breakthrough patients began to increase in the early summer of 2021, the necessity of a third dose of COVID-19 vaccine was being comprehensively discussed and analyzed, which still warrants intensive scientific interest and practical importance. In view that our study suggested a second dose of vaccine is more effective in protecting individuals against COVID-19 variants compared with receiving only one dose, it is reasonable to presume that a higher level of protection could be observed in those who completed the three-dose vaccine regime. Admittedly, it is indeed a valid point that a third booster could relieve potential waning vaccine-induced humoral and cellular immunity, possibly increasing immune escape and reducing the effectiveness of vaccines against SARS-CoV-2 variants over time. The findings of Barda et al. demonstrated that a third dose of the BNT162b2 vaccine could address severe COVID-19-related outcomes compared with the standard two-dose strategy (85). In a study of heterologous vaccination, health-care workers in Thailand who received a third dose of ChAdOx1 after completing a two-dose CoronaVac vaccine regime elicited higher neutralizing activity against all variants of concern (86). Thompson et al. emphasized that all unvaccinated adults should get vaccinated with a third dose of an mRNA vaccine as soon as possible when considering that the mRNA vaccine effectiveness was 90 and 82% ≥14 days after dose 3 during the Delta and Omicron predominant periods, respectively (87). Moreover, a booster third dose is necessary for cancer patients, organ transplant recipients, people aged >60 years, etc., whose immune responses are inadequate (88–90). Nevertheless, a third vaccine dose may seem like a luxury and nothing could be more urgent than the elimination of vaccine discrimination and vaccine inequity. Firstly, worldwide vaccine campaigns remain extremely unfair. Numerous industrialized countries such as the UK and the USA have managed to fully vaccinate >60% or covered 50% of their populations, whereas some countries in African have shockingly low vaccination coverage in their population. The administration of a third booster dose is expected to further damage the disequilibrium and it has become an ethical issue (91). Secondly, it remains unclear whether there is an upper limit of mutation, beyond which SARS-COV-2 would not evolve in respect to transmission, virulence, or immune evasion (92). When the ceiling is overcome, for example, a hyperexponential increase in the transmissibility, the need of a third dose and the implementation of Draconian measures are much more valuable (93). Last but not least, vaccine discrimination and vaccine inequity will encourage viral epidemic relapses, even in developed countries with broad vaccination coverage. People should be aware that in an infected individual without vaccination the virus is more prone to mutations than in a vaccinated person (94), and the viral mutation potential is higher in countries that have lower vaccination coverage (95). Thus, we think that the two-dose vaccine schedule could achieve the initial target to prevent COVID-19 variant infection, but in the meantime, a third booster dose is necessary for patients with inadequate immune responses or people who need to safeguard against Omicron immune escape.

For the subgroup-analyses according to different types of vaccines, we found that the incidence of overall variants and the efficacy of a specific vaccine post first mRNA vaccine (BNT162b2/mRNA-1273/JNJ-78436735) were 0.07 and 35%, and post second dose were 0.06 and 85%, respectively; the incidence of overall variants and the efficacy of a specific vaccine post second viral vector vaccine (ChAdOx1/ChAdOx1-S) were 0.02 and 66%, respectively; the efficacy of a specific vaccine post first inactivated vaccine (CoronaVac) was 37%. As the results showed, a two-dose regimen of an mRNA vaccine was more effective against COVID-19 variants than a traditional viral vector vaccine and inactivated vaccine compared with the placebo group or unvaccinated populations. As a gene-based vaccine, BNT162b2 became an mRNA vaccine candidate and went from concept to clinical development in <3months, a rate unprecedented in the history of vaccine development (20). Phase III clinical trials and real-world data showed that a two-dose procedure of BNT162b2 could effectively prevent individuals across all age groups from infections with or without COVID-19 symptoms, and in the meantime significantly reduce the incidence of hospitalizations and decrease the rate of severe disease and death caused by COVID-19 infections (24, 25, 28, 29, 96). mRNA vaccines could elicit broad immune responses against a wide range of SARS-CoV-2 variants, including neutralizing antibodies combined with CD4^+^ and CD8^+^ T cells, which may be responsible for the significant efficacy of BNT162b2/mRNA-1273/JNJ-78436735 (38, 40, 48, 52, 53, 57, 97). Viral vector vaccines and inactivated vaccines are both based on traditional platforms. ChAdOx1 contains the replicated defective adenovirus gene encoding the spike protein of SARS-CoV-2. Although several studies confirmed that ChAdOx1 could elicit specific neutralizing antibodies and an immune response mediated by T cells against SARS-CoV-2, the pooled efficacy of ChAdOx1 was lower than mRNA vaccines (80.2 vs. 94.6%) (30, 98, 99). CoronaVac/BBV152, as a vaccine containing inactivated SARS-CoV-2 that could be suitable for mass production and stably express antibodies with good immunologic tolerant, had fine effectiveness against COVID-19 confirmed by PCR (23, 51, 100). However, it is worth noting that some studies demonstrated that the efficacy of CoronaVac was only 50.39% and it could not induce immune memory (35, 101). Unfortunately, data for the Pro-Subunit and other types of vaccines were not available, hence, the analysis of these vaccines was not included in our study. Just from the respect of efficacy, we recommend mRNA vaccines as the “first-order” promising candidate against COVID-19 variants.

B.1.1.7, containing D614G and eight other spike mutations, was first detected in the UK on 14 December 2020 (66). This variant could enhance transmissibility up to 71% and caused mortality to increase substantially compared with previous mutations (66, 102). We found that the incidence of B.1.1.7 and the effectiveness of vaccines against B.1.1.7 post a second vaccine were 0.04 and 90%, respectively. This moderate effectiveness may be the proof that B.1.1.7 did not demonstrate enhanced immune escape capability. In addition, the efficacy of an mRNA vaccine and vector vaccine against B.1.1.7 post second dose were 89 and 94%, respectively. The difference in the efficacy against B.1.1.7 between BNT162b2 and ChAdOx1 is well grounded in neutralization tests and clinical trials. Muik et al. found the immune sera induced by BNT162b2 generally retained immunocompetence against B.1.1.7 even though there was a slight reduction (73), but Gavin et al. reported that the sera-neutralizing titers induced by ChAdOx1 showed a 2.1–2.5-fold reduction against B.1.1.7 (103). In the real-world setting, the studies of Hall et al. (27), Abu-Raddad et al. (24), and Munitz et al. (74) concluded that the mRNA vaccine of BNT162b2 could prevent the infection of SARS-COV-2 when B1.1.7 was the dominant variant, whereas, Emary et al. (30) found that the efficacy of ChAdOx1 against symptomatic B.1.1.7 patients was 70.4%, which was obviously lower than for non-B.1.1.7 infections (81.5%). B.1.351, containing D614G and nine other spike mutations, was first identified on 18 December 2020 in South Africa (66). This variant caused much greater concern because the diminished protective effectiveness of the current vaccines meant that the South African vaccination strategy completely shifted (104). Our results showed that the incidence of B.1.351 and the effectiveness of vaccines against B.1.351 post second vaccine were 0.09 and 42%, respectively, which indicated that the vaccines provided a less effective protection against B.1.351 than against B.1.1.7. Moreover, the incidence of the B.1.351 variant post second BNT162b2 dose and the effectiveness of the mRNA vaccine against B.1.351 were 0.10 and 40%, respectively, which also demonstrated that the prevention ability of BNT162b2 against B.1.351 decreased significantly when compared with B.1.1.7. The downward tendency among the neutralizing abilities of vaccines against B.1.351 and B.1.1.7 was consistent with our findings. The study by Gavin et al. showed that the decline in the neutralizing abilities against B.1.351 was 7.6-fold but against B.1.1.7 was only 3.3-fold (105). Furthermore, results from Wang et al. revealed that the average loss in neutralization titers against B.1.1.7/B.1.351 was 2/6.5-fold, respectively (68). Liu and Xie et al. (106, 107) believe the drop in neutralization titers against B.1.351 in sera induced by the vaccine could be mainly due to E484K mutation, which is located at the region of the receptor-binding domain (RBD). Our results could also be confirmed by the real-world condition reported by Abu-Raddad et al. (24) who performed a cohort study in Qatar and found that the effectiveness of BNT162b2 was estimated to be 87.0% against B.1.1.7 and 72.1% against the B.1.351. P.1 (Gamma) variant. This variant, which harbors 17 nonsynonymous mutations, was detected in Brazil, and first reported in the USA, showed a 2.6 times more transmissible capacity and significantly increased the risk of hospitalization and ICU admission (66). Similar to the results of B.1.351, the efficacy of vaccines against P.1, including mRNA and viral vector vaccines, were abolished in our study and fall in line with the results of a nationwide study by Wibmer et al. in France which showed that the effectiveness of the mRNA vaccine was estimated at 77% [95% CI:0.63, 0.86] (108). Although the neutralization of convalescent plasma and vaccine sera was reduced by 3.8–4.8-fold during the P.1 epidemic (109), we perceived that the threat posed by P.1 could not be as severe as previous variants in view that the diminution of vaccine protection against P.1 was not as great as B.1.351 and others. The B.1.617.2 (Delta) variant with 10 mutations in the spike protein was initially considered a VOI (variant of interest), but was rapidly classified as a VOC by WHO in view of its sharp rise in infections and mortality. It appears that the ongoing vaccines still offer substantial protection against the B.1.617.2 (Delta) variant, at slightly higher levels compared with P.1 on the basis of the findings in our study. Our results could also be further reproduced in several meta-analyses and neutralization tests, which reported that the B.1.617.2 variant could be neutralized by post-vaccination sera and convalesced successfully with only a mild decrease in its neutralization sensitivity and confirmed that current vaccines could offer higher protection against B.1.617.2 in real-world settings (110, 111). B.1.427 (Epsilon), first identified in California, increased transmissibility by approximately 20% and exhibited moderate resistance to neutralization when using convalescent and post-vaccination sera. However, the efficacy of pooled vaccines against B.1.427 was 95% and, thus we considered the completion of a two-dose vaccine schedule to have a favorable protective effect which helped explain why B.1.427 was classified as a VOC only in the USA but a VOI in other countries (15). Due to the lack of sufficient data about other types of vaccines such as Pro-Subunit and inactivated vaccines and other types of variants such as P.2 and B.1.526, it is regrettable that only a few incidences of some specific vaccines post one or two specific doses could be pooled, which were hard to explain and verify by neutralization tests and clinical trials in a real-world setting.

Yet, there are, at the moment, limited data to systematically evaluate the effectiveness of the existing vaccines against B.1.1.529 (Omicron), which is the fifth VOC categorized by WHO and has become the most widely distributed variant since December 2021. It is suggested that the viral infectivity of Omicron increases 2.8-fold compared to B.1.617.2 which could contribute to the explosive rise in cases (112). Mutations in Omicron, which are responsible for more vaccine breakthroughs and have an overwhelmingly disruptive effect, could substantially reduce or impair the neutralization by monoclonal antibodies (mAbs), convalescent plasma, and vaccine sera compared to mutations in predecessor variants (113–115). Importantly, SARS-CoV-2 may not have reached the top of its evolution and Omicron is perceived to have opened up the broadly untapped potential for future mutations, which may possess more virulent strains and severely affect the global population (116). In this present scenario, it is unlikely that the ongoing vaccines will completely fail against Omicron, considering the findings in our study that the previous VOCs (Alpha, Beta, Gamma, and Delta) have been curbed by COVID-19 vaccines. Dejnirattisai et al. reported that the sterilizing immunity against Omicron induced by vaccines may be diminished, however, cell-mediated immunity might be less affected and ensure that vaccines are still useful in terms of containing infection progression, etc. (113). Most neutralization assays about Omicron were performed *in vivo* which did not fully quantify the immune response *in vivo*. The booster third dose of vaccines, including mRNA, viral vector, and inactivated vaccines, could significantly enhance the neutralizing activity against Omicron both *in vivo* and *in vivo* (85–90, 117, 118). Hence, we perceived that the impact of Omicron has not yet threatened global conformational alterations, and vaccines may still protect people from COVID-19 variants until further information is available.

The strength of this meta-analysis lies in its rapid analysis of the incidence of variants in the COVID-19 pandemic and the efficacy of current vaccines against these variants, which could provide useful insight for the implementation of COVID-19 vaccination in the setting of numerous variants. In the meantime, we must acknowledge that the results of our study should be interpreted with a very cautious approach because it was subject to certain limitations that warrant mention. Firstly, most of the included articles were cohort studies or observational studies, which could not provide the sufficient statistical power of randomized controlled trials (RCTs). Besides, high statistical heterogeneity existed for some subgroup analyses and must be considered when interpreting the outcomes. Secondly, some studies included insufficient or inexact numbers of participants or variants, which suggested there was a contingent risk of misestimation of the incidence of variants or the efficacy of the vaccines. Thirdly, up to now, most of the included vaccines and variants were mRNA vaccines or vector vaccines and B.1.1.7, B.1.351, etc., respectively. Some current vaccines and variants were not brought into the present study because of the incomplete data. Thus, the summaries of the clinical trials may not coincide with the real world reality, and the generalizability of our findings is unknown. Last but not least, the safety or the adverse events of COVID-19 vaccines and the ability to spread or virulence of the variants were not evaluated in our study, which might lead to one-sidedness in a comprehensive understanding of COVID-19 vaccines against variants.

In this study, we first presented the preliminary conclusions about the results of the current vaccines against the emerging variants. According to the situation, scientists around the world are focusing on learning more about whether the current authorized vaccines will protect people from infection caused by SARS-CoV-2 variants in the real world. The next generation of vaccines, such as a bivalent vaccine by Johnson & Johnson, a booster vaccine by Moderna, mRNA multivalent vaccines by GlaxoSmithKline and CVNV, etc., might play a pivotal role in preventing and controlling the variants of SARS-CoV-2 worldwide.

## Conclusion

Our meta-analysis shows that the current vaccines that are used globally could restrict the spread and prevent infection of SARS-CoV-2 variants to a great extent. We would also support maximizing vaccine uptake with two doses as the most effective compared to only one dose. Although the mRNA vaccine was found to be the most effective against variants in our study, specific vaccines should be taken into account based on the local dominant prevalence of variants. Furthermore, the conclusions should be used cautiously in consideration of the limited data. In the future, we emphasize the importance of continued testing and case management which will be further elucidate whether vaccines play a protective role against the ongoing evolution of SARS-CoV-2 variants.

## Data Availability Statement

The original contributions presented in the study are included in the article/Supplementary Material, further inquiries can be directed to the corresponding authors.

## Author Contributions

NH, KW, YZ, and FJ designed the work. NH, KW, BX, LW, LH, YZ, and MW performed the literature review and data abstraction. NH, KW, ML, RZ, and FJ were involved in the statistical analysis. All authors read and approved the final manuscript.

## Funding

This work was supported by grants from the Science and Technology Development Project of Medical & Health of Shandong Province (Nos. 202010000131 and 202104070065) and the Key Research and Development Project of Zibo (Policy Guidance program) (No. 2020ZC010048). There was no sponsorship involved in these projects.

## Conflict of Interest

The authors declare that the research was conducted in the absence of any commercial or financial relationships that could be construed as a potential conflict of interest.

## Publisher's Note

All claims expressed in this article are solely those of the authors and do not necessarily represent those of their affiliated organizations, or those of the publisher, the editors and the reviewers. Any product that may be evaluated in this article, or claim that may be made by its manufacturer, is not guaranteed or endorsed by the publisher.

## Supplementary Material

Supplementary Figure 1 Subgroup analysis for the pooled incidence of variants post first mRNA vaccine **(A)**, variants post second mRNA vaccine **(B)**, variants post second viral vector vaccine **(C)**, variants post second protein subunit vaccine **(D)**, variants post second inactivated vaccine **(E)**, B.1.1.7 (Alpha) variant post first vaccine **(F)**, B.1.1.7 (Alpha) variant post second vaccine **(G)**, B.1.1.7 (Alpha) variant post first mRNA vaccine **(H)**, B.1.1.7 (Alpha) variant post second mRNA vaccine **(I)**, B.1.1.7 (Alpha) variant post first viral vector vaccine **(J)**, B.1.1.7 (Alpha) variant post second viral vector vaccine **(K)**, B.1.1.7 (Alpha) variant post second protein subunit vaccine **(L)**, B.1.351 (Beta) variant post first vaccine **(M)**, B.1.351 (Beta) variant post second vaccine **(N)**, B.1.351 (Beta) variant post first mRNA vaccine **(O)**, B.1.351 (Beta) variant post second mRNA vaccine **(P)**, B.1.351 (Beta) variant post second viral vector vaccine **(Q)**, B.1.351 (Beta) variant post second protein subunit vaccine **(R)**, P.1 (Gamma) variant post first vaccine **(S)**, P.1 (Gamma) variant post second vaccine **(T)**, P.1 (Gamma) variant post first mRNA vaccine **(U)**, P.1 (Gamma) variant post second mRNA vaccine **(V)**, P.1 (Gamma) variant post second inactivated vaccine **(W)**, P.1 (Gamma) variant post second viral vector vaccine **(X)**, B.1.617.2 (Delta) variant post first vaccine **(Y)**, B.1.617.2 (Delta) variant post second vaccine Zz), B.1.617.2 (Delta) variant post first mRNA vaccine **(AA)**, B.1.617.2 (Delta) variant post second mRNA vaccine **(BB)**, B.1.617.2 (Delta) variant post first viral vector vaccine **(CC)**, B.1.617.2 (Delta) variant post second viral vector vaccine **(DD)**, B.1.427 (Epsilon) variant post first vaccine **(EE)**, B.1.427 (Epsilon) variant post second vaccine **(FF)**, B.1.427 (Epsilon) variant post first mRNA vaccine **(GG)**, B.1.427 (Epsilon) variant post second mRNA vaccine **(HH)**, P.2 (Zeta) variant post second vaccine **(II)**, B.1.526 (Iota) variant post second vaccine **(JJ)**, and B.1.526 (Iota) variant post second mRNA vaccine **(KK)**. ^*^ and ^**^ indicate the second and third arm in the corresponding studies, respectively. CI, confidence interval; OR, odds ratio.

Supplementary Figure 2 Subgroup analysis for the pooled efficacy of mRNA vaccine against variants post first dose **(A)**, mRNA vaccine against variants post second dose **(B)**, viral vector vaccine against variants post second dose **(C)**, vaccines against the B.1.1.7 (Alpha) variant post first dose **(D)**,vaccines against the B.1.1.7 (Alpha) variant post second dose **(E)**, mRNA vaccine against the B.1.1.7 (Alpha) variant post first dose **(F)**, mRNA vaccine against the B.1.1.7 (Alpha) variant post second dose **(G)**, subunit vaccine against the B.1.1.7 (Alpha) variant post second dose **(H)**, viral vector vaccine against the B.1.1.7 (Alpha) variant post second dose **(I)**, vaccines against the B.1.351 (Beta) variant post first dose **(J)**, vaccines against the B.1.351 (Beta) variant post second dose **(K)**, mRNA vaccine against the B.1.351 (Beta) variant post first dose **(L)**, mRNA vaccine against the B.1.351 (Beta) variant post second dose **(M)**, vaccines against the P.1 (Gamma) variant post first dose **(N)**, vaccines against the P.1 (Gamma) variant post second dose **(O)**, mRNA vaccine against the P.1 (Gamma) variant post first dose **(P)**, mRNA vaccine against the P.1 (Gamma) variant post second dose **(Q)**, viral vector vaccine against the P.1 (Gamma) variant post second dose **(R)**, vaccines against the B.1.617.2 (Delta) variant post first dose **(S)**, vaccines against the B.1.617.2 (Delta) variant post second dose **(T)**, mRNA vaccine against the B.1.617.2 (Delta) variant post second dose **(U)**, viral vector vaccine against the B.1.617.2 (Delta) variant post first dose **(V)**, viral vector vaccine against the B.1.617.2 (Delta) variant post second dose **(W)**, vaccines against the B.1.427 (Epsilon) variant post first dose **(X)**, vaccines against the B.1.427 (Epsilon) variant post second dose **(Y)**, mRNA vaccine against the B.1.427 (Epsilon) variant post first dose **(Z)**, mRNA vaccine against the B.1.427 (Epsilon) variant post second dose **(AA)**, vaccines against the P.2 (Zeta) variant post second dose **(BB)**, vaccines against the B.1.526 (Iota) variant post second dose **(CC)**, and mRNA vaccines against the B.1.526 (Iota) variant post second dose **(DD)**. ^*^ and ^**^ indicate the second and third arm in the corresponding studies, respectively. CI, confidence interval; OR, odds ratio.

Supplementary Table 1 Characteristics of the studies included.

Supplementary Table 2 Results of the Egger test, the heterogeneity test, and the meta-analysis.

## Abbreviations

OR: Odds ratio
VE: Vaccine effectiveness
CI: Confidence intervals
COVID-19: Coronavirus disease 2019
SARS-CoV-2: Severe acute respiratory syndrome coronavirus 2
RNA: Ribonucleic acid
WHO: World Health Organization
EUL: Emergency use listing
PQ: Prequalification
VOIs: Variants of interest
VOCs: Variants of concern
CDC: The Centers for Disease Control and Prevention
PRISMA: The Preferred Reporting Items for Systematic Reviews and Meta-Analyses
MOOSE: Meta-analyses of Observational Studies in Epidemiology
PICOS, Population, intervention/exposure, comparator, outcome: and study
C.Ts: Comparative trials
C.Ss: Cohort studies
O.Ss: Observational studies
SD: Standard deviation
RBD: Receptor-binding domain
mAbs: Monoclonal antibodies.
