# Supplementary material for: Real-Word Effectiveness of Global COVID-19 Vaccines Against SARS-CoV-2 Variants: A Systematic Review and Meta-Analysis
Source: Front Med (Lausanne). 2022 May 19;9:820544. doi: 10.3389/fmed.2022.820544 (PMC9160927; doi:10.3389/fmed.2022.820544)
Supplement: Supplementary Figure 1 — Subgroup analysis for the pooled incidence of variants post first mRNA vaccine (A), variants post second mRNA vaccine (B), variants post second viral vector vaccine (C), variants post second protein subunit vaccine (D), variants post second inactivated vaccine (E), B.1.1.7 (Alpha) variant post first vaccine (F), B.1.1.7 (Alpha) variant post second vaccine (G), B.1.1.7 (Alpha) variant post first mRNA vaccine (H), B.1.1.7 (Alpha) variant post second mRNA vaccine (I), B.1.1.7 (Alpha) variant post first viral vector vaccine (J), B.1.1.7 (Alpha) variant post second viral vector vaccine (K), B.1.1.7 (Alpha) variant post second protein subunit vaccine (L), B.1.351 (Beta) variant post first vaccine (M), B.1.351 (Beta) variant post second vaccine (N), B.1.351 (Beta) variant post first mRNA vaccine (O), B.1.351 (Beta) variant post second mRNA vaccine (P), B.1.351 (Beta) variant post second viral vector vaccine (Q), B.1.351 (Beta) variant post second protein subunit vaccine (R), P.1 (Gamma) variant post first vaccine (S), P.1 (Gamma) variant post second vaccine (T), P.1 (Gamma) variant post first mRNA vaccine (U), P.1 (Gamma) variant post second mRNA vaccine (V), P.1 (Gamma) variant post second inactivated vaccine (W), P.1 (Gamma) variant post second viral vector vaccine (X), B.1.617.2 (Delta) variant post first vaccine (Y), B.1.617.2 (Delta) variant post second vaccine Zz), B.1.617.2 (Delta) variant post first mRNA vaccine (AA), B.1.617.2 (Delta) variant post second mRNA vaccine (BB), B.1.617.2 (Delta) variant post first viral vector vaccine (CC), B.1.617.2 (Delta) variant post second viral vector vaccine (DD), B.1.427 (Epsilon) variant post first vaccine (EE), B.1.427 (Epsilon) variant post second vaccine (FF), B.1.427 (Epsilon) variant post first mRNA vaccine (GG), B.1.427 (Epsilon) variant post second mRNA vaccine (HH), P.2 (Zeta) variant post second vaccine (II), B.1.526 (Iota) variant post second vaccine (JJ), and B.1.526 (Iota) variant post second mRNA vaccine (KK). * a [file Data_Sheet_1.pdf]

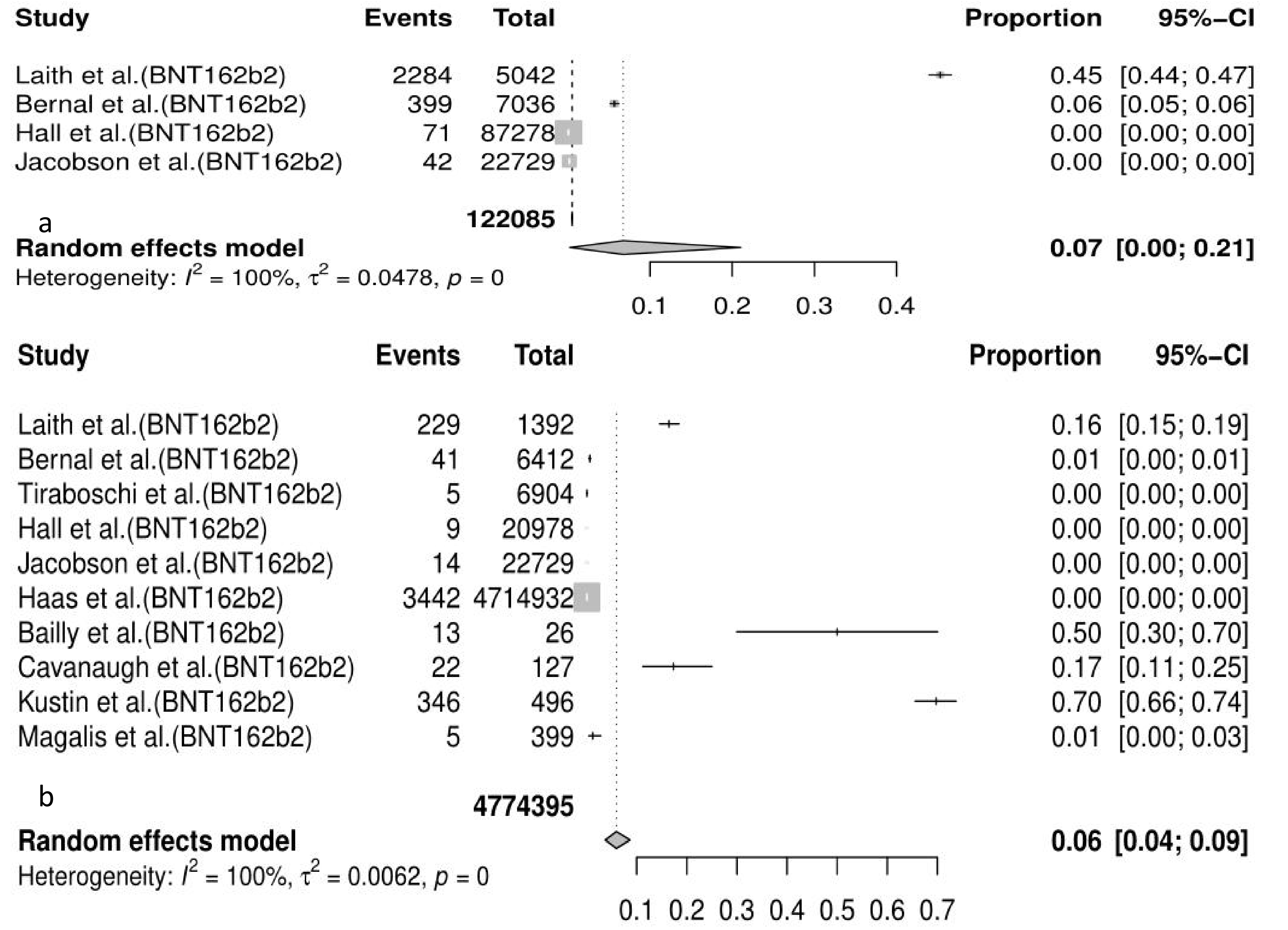
Study Events Total Proportion 95%-CI
Laith et al.(BNT162b2) 2284 5042 0.45 [0.44; 0.47]
Bernal et al.(BNT162b2) 399 7036 0.06 [0.05; 0.06]
Hall et al.(BNT162b2) 71 87278 0.00 [0.00; 0.00]
Jacobson et al.(BNT162b2) 42 22729 0.00 [0.00; 0.00]
122085
a
Random effects model 0.07 [0.00; 0.21]
Heterogeneity: I^2 = 100%, τ^2 = 0.0478, p = 0
0.1 0.2 0.3 0.4
Study Events Total Proportion 95%-CI
Laith et al.(BNT162b2) 229 1392 0.16 [0.15; 0.19]
Bernal et al.(BNT162b2) 41 6412 0.01 [0.00; 0.01]
Tiraboschi et al.(BNT162b2) 5 6904 0.00 [0.00; 0.00]
Hall et al.(BNT162b2) 9 20978 0.00 [0.00; 0.00]
Jacobson et al.(BNT162b2) 14 22729 0.00 [0.00; 0.00]
Haas et al.(BNT162b2) 3442 4714932 0.00 [0.00; 0.00]
Bailly et al.(BNT162b2) 13 26 0.50 [0.30; 0.70]
Cavanaugh et al.(BNT162b2) 22 127 0.17 [0.11; 0.25]
Kustin et al.(BNT162b2) 346 496 0.70 [0.66; 0.74]
Magalis et al.(BNT162b2) 5 399 0.01 [0.00; 0.03]
b
4774395
Random effects model 0.06 [0.04; 0.09]
Heterogeneity: I^2 = 100%, τ^2 = 0.0062, p = 0
0.1 0.2 0.3 0.4 0.5 0.6 0.7

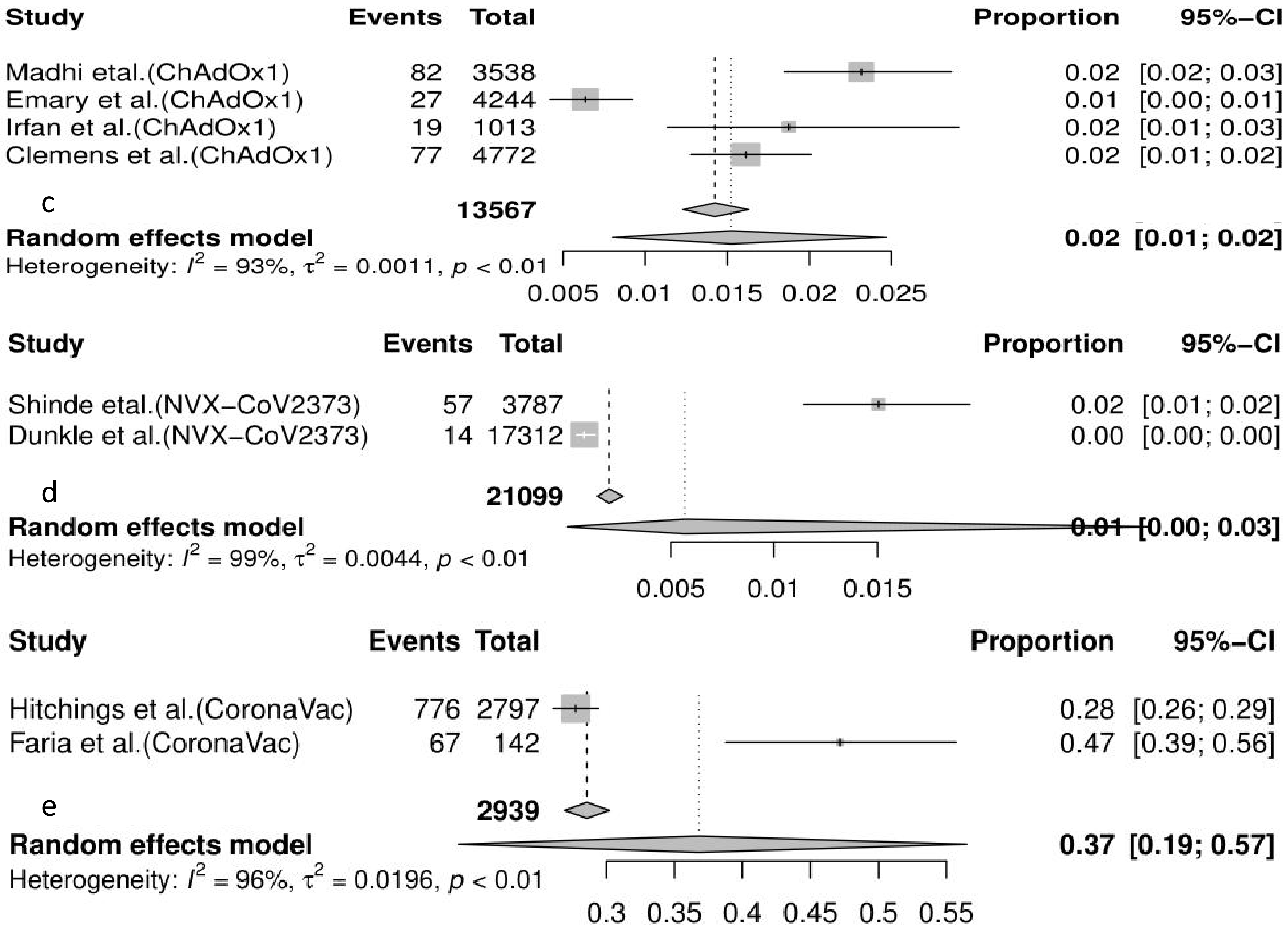
Study Events Total Proportion 95%−CI
Madhi etal.(ChAdOx1) 82 3538 0.02 [0.02; 0.03]
Emary et al.(ChAdOx1) 27 4244 0.01 [0.00; 0.01]
Irfan et al.(ChAdOx1) 19 1013 0.02 [0.01; 0.03]
Clemens et al.(ChAdOx1) 77 4772 0.02 [0.01; 0.02]
c
13567
Random effects model 0.02 [0.01; 0.02]
Heterogeneity: I^2 = 93%, τ^2 = 0.0011, p < 0.01
0.005 0.01 0.015 0.02 0.025
Study Events Total Proportion 95%−CI
Shinde etal.(NVX−CoV2373) 57 3787 0.02 [0.01; 0.02]
Dunkle et al.(NVX−CoV2373) 14 17312 0.00 [0.00; 0.00]
d
21099
Random effects model 0.01 [0.00; 0.03]
Heterogeneity: I^2 = 99%, τ^2 = 0.0044, p < 0.01
0.005 0.01 0.015
Study Events Total Proportion 95%−CI
Hitchings et al.(CoronaVac) 776 2797 0.28 [0.26; 0.29]
Faria et al.(CoronaVac) 67 142 0.47 [0.39; 0.56]
e
2939
Random effects model 0.37 [0.19; 0.57]
Heterogeneity: I^2 = 96%, τ^2 = 0.0196, p < 0.01
0.3 0.35 0.4 0.45 0.5 0.55

Study	Events	Total	Proportion	95%-CI
Laith et al.(BNT162b2)	892	2133	0.42	[0.40; 0.44]
Bernal et al.(BNT162b2)	49	7036	0.01	[0.01; 0.01]
Bernal et al.(ChAdOx1)*	230	25667	0.01	[0.01; 0.01]
Hall et al.(BNT162b2)	71	87278	0.00	[0.00; 0.00]
Pouwels et al.(BNT162b2)	714	219626	0.00	[0.00; 0.00]
Pouwels et al.(ChAdOx1)*	542	379437	0.00	[0.00; 0.00]
Trunfio et al.(BNT162b2)	45	899	0.05	[0.04; 0.07]
Chemaitelly et al.(BNT162b2)	63	154	0.41	[0.33; 0.49]
Tenforde et al.(BNT162b2/mRNA−1273)	28	354	0.08	[0.05; 0.11]
Bruxvoort et al.(mRNA−1273)	14	649	0.02	[0.01; 0.04]
Dickerman et al.(BNT162b2)	1135	219842	0.01	[0.00; 0.01]
Dickerman et al.(mRNA−1273)*	881	219842	0.00	[0.00; 0.00]
Abu−Raddad et al.(BNT162b2)	773	1888	0.41	[0.39; 0.43]
Eyre et al.(ChAdOx1)	3739	12307	0.30	[0.30; 0.31]
Eyre et al.(BNT162b2)*	3829	20999	0.18	[0.18; 0.19]
		**1198111**		
**Random effects model**			**0.07**	**[0.05; 0.10]**

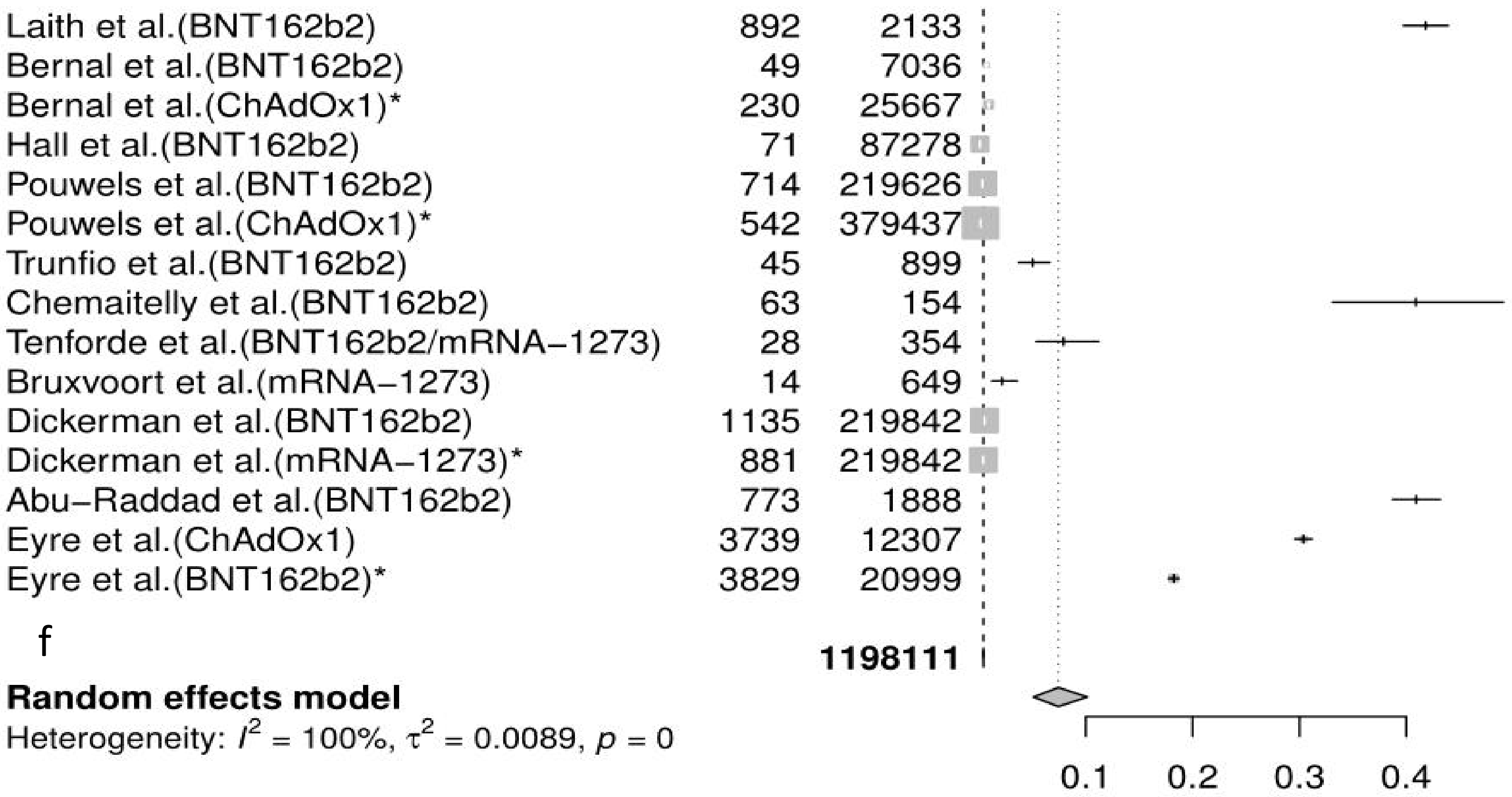

f

Heterogeneity: $I^2 = 100\%$, $\tau^2 = 0.0089$, $p = 0$

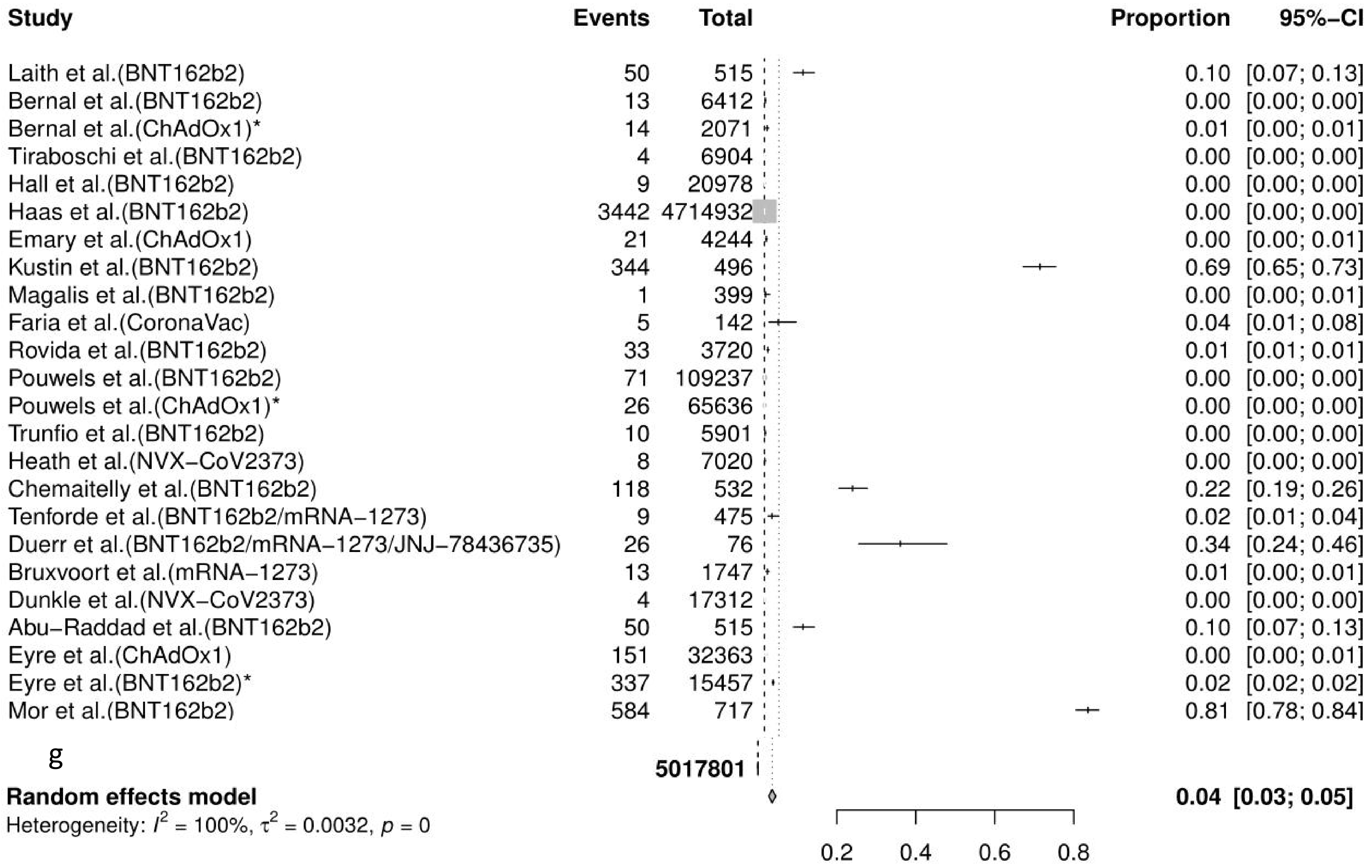
Study Events Total Proportion 95%−CI
Laith et al.(BNT162b2) 50 515 0.10 [0.07; 0.13]
Bernal et al.(BNT162b2) 13 6412 0.00 [0.00; 0.00]
Bernal et al.(ChAdOx1)* 14 2071 0.01 [0.00; 0.01]
Tiraboschi et al.(BNT162b2) 4 6904 0.00 [0.00; 0.00]
Hall et al.(BNT162b2) 9 20978 0.00 [0.00; 0.00]
Haas et al.(BNT162b2) 3442 4714932 0.00 [0.00; 0.00]
Emary et al.(ChAdOx1) 21 4244 0.00 [0.00; 0.01]
Kustin et al.(BNT162b2) 344 496 0.69 [0.65; 0.73]
Magalis et al.(BNT162b2) 1 399 0.00 [0.00; 0.01]
Faria et al.(CoronaVac) 5 142 0.04 [0.01; 0.08]
Rovida et al.(BNT162b2) 33 3720 0.01 [0.01; 0.01]
Pouwels et al.(BNT162b2) 71 109237 0.00 [0.00; 0.00]
Pouwels et al.(ChAdOx1)* 26 65636 0.00 [0.00; 0.00]
Trunfio et al.(BNT162b2) 10 5901 0.00 [0.00; 0.00]
Heath et al.(NVX−CoV2373) 8 7020 0.00 [0.00; 0.00]
Chemaitelly et al.(BNT162b2) 118 532 0.22 [0.19; 0.26]
Tenforde et al.(BNT162b2/mRNA−1273) 9 475 0.02 [0.01; 0.04]
Duerr et al.(BNT162b2/mRNA−1273/JNJ−78436735) 26 76 0.34 [0.24; 0.46]
Bruxvoort et al.(mRNA−1273) 13 1747 0.01 [0.00; 0.01]
Dunkle et al.(NVX−CoV2373) 4 17312 0.00 [0.00; 0.00]
Abu−Raddad et al.(BNT162b2) 50 515 0.10 [0.07; 0.13]
Eyre et al.(ChAdOx1) 151 32363 0.00 [0.00; 0.01]
Eyre et al.(BNT162b2)* 337 15457 0.02 [0.02; 0.02]
Mor et al.(BNT162b2) 584 717 0.81 [0.78; 0.84]
g
5017801
Random effects model 0.04 [0.03; 0.05]
Heterogeneity: I^2 = 100%, τ^2 = 0.0032, p = 0
0.2 0.4 0.6 0.8

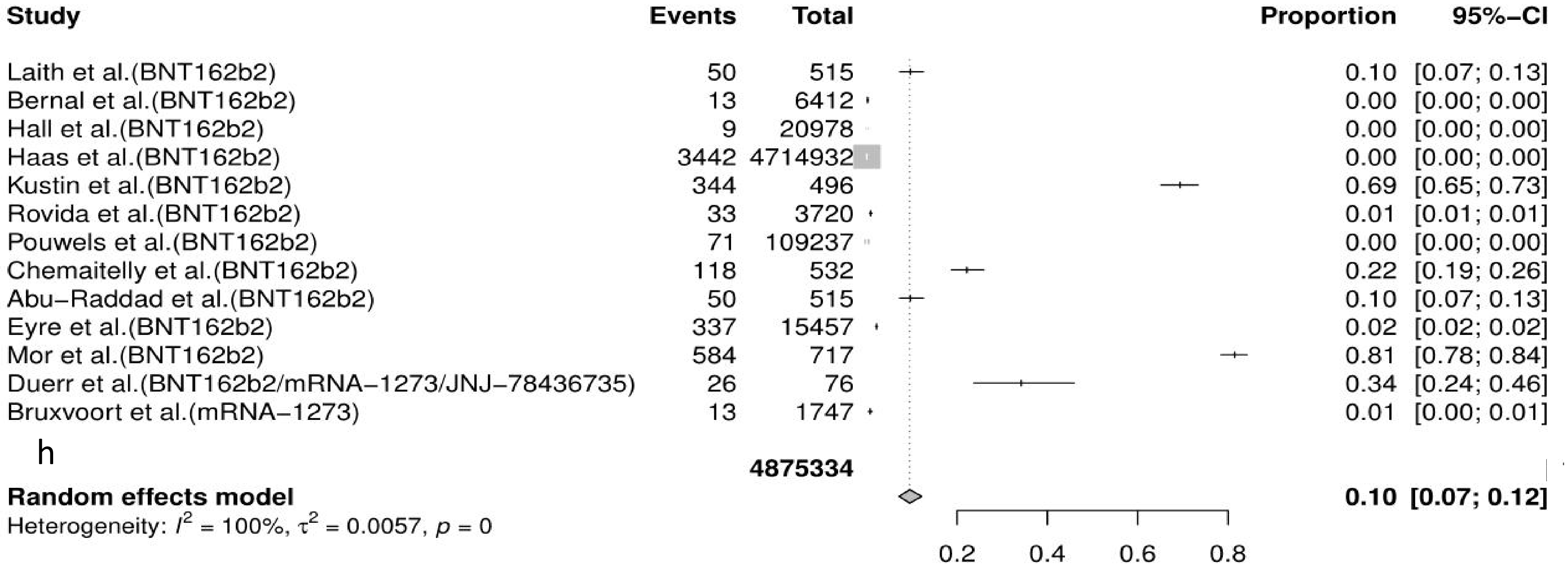
Study Events Total Proportion 95%-CI
Laith et al.(BNT162b2) 50 515 0.10 [0.07; 0.13]
Bernal et al.(BNT162b2) 13 6412 0.00 [0.00; 0.00]
Hall et al.(BNT162b2) 9 20978 0.00 [0.00; 0.00]
Haas et al.(BNT162b2) 3442 4714932 0.00 [0.00; 0.00]
Kustin et al.(BNT162b2) 344 496 0.69 [0.65; 0.73]
Rovida et al.(BNT162b2) 33 3720 0.01 [0.01; 0.01]
Pouwels et al.(BNT162b2) 71 109237 0.00 [0.00; 0.00]
Chemaitelly et al.(BNT162b2) 118 532 0.22 [0.19; 0.26]
Abu−Raddad et al.(BNT162b2) 50 515 0.10 [0.07; 0.13]
Eyre et al.(BNT162b2) 337 15457 0.02 [0.02; 0.02]
Mor et al.(BNT162b2) 584 717 0.81 [0.78; 0.84]
Duerr et al.(BNT162b2/mRNA−1273/JNJ−78436735) 26 76 0.34 [0.24; 0.46]
Bruxvoort et al.(mRNA−1273) 13 1747 0.01 [0.00; 0.01]
h
4875334
Random effects model 0.10 [0.07; 0.12]
Heterogeneity: I^2 = 100%, τ^2 = 0.0057, p = 0
0.2 0.4 0.6 0.8

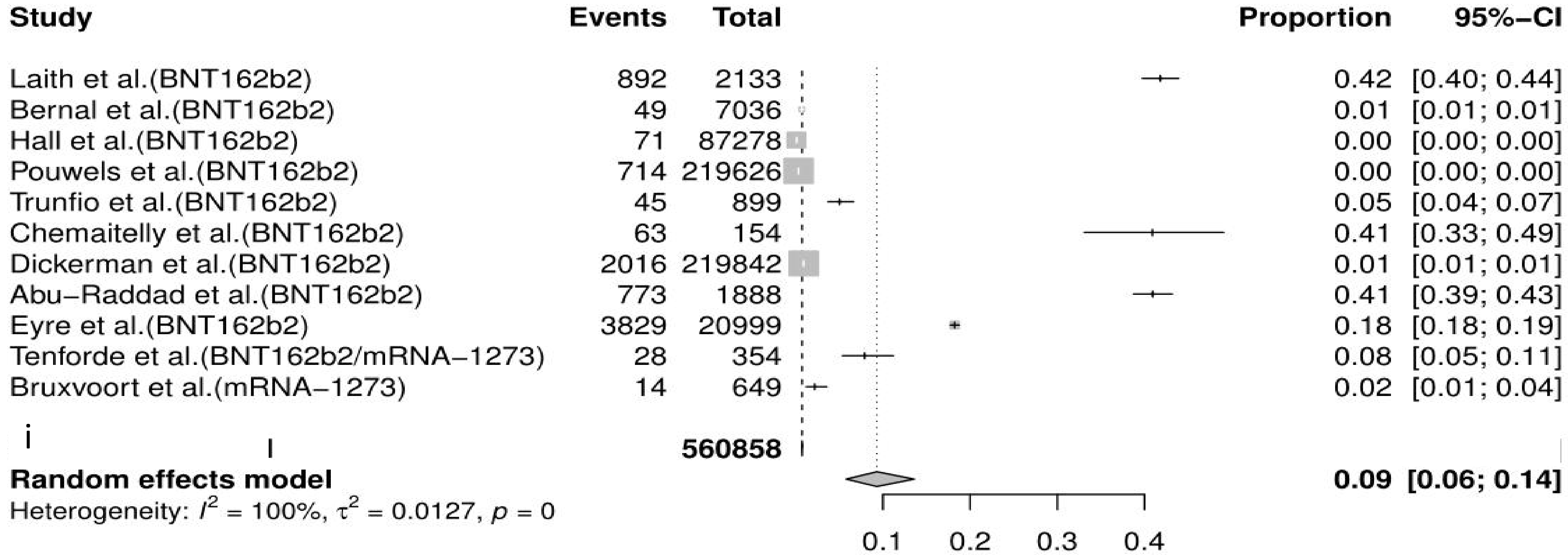
Study Events Total Proportion 95%-CI
Laith et al.(BNT162b2) 892 2133 0.42 [0.40; 0.44]
Bernal et al.(BNT162b2) 49 7036 0.01 [0.01; 0.01]
Hall et al.(BNT162b2) 71 87278 0.00 [0.00; 0.00]
Pouwels et al.(BNT162b2) 714 219626 0.00 [0.00; 0.00]
Trunfio et al.(BNT162b2) 45 899 0.05 [0.04; 0.07]
Chemaitelly et al.(BNT162b2) 63 154 0.41 [0.33; 0.49]
Dickerman et al.(BNT162b2) 2016 219842 0.01 [0.01; 0.01]
Abu−Raddad et al.(BNT162b2) 773 1888 0.41 [0.39; 0.43]
Eyre et al.(BNT162b2) 3829 20999 0.18 [0.18; 0.19]
Tenforde et al.(BNT162b2/mRNA−1273) 28 354 0.08 [0.05; 0.11]
Bruxvoort et al.(mRNA−1273) 14 649 0.02 [0.01; 0.04]
i
560858
Random effects model 0.09 [0.06; 0.14]
Heterogeneity: I^2 = 100%, τ^2 = 0.0127, p = 0
0.1 0.2 0.3 0.4

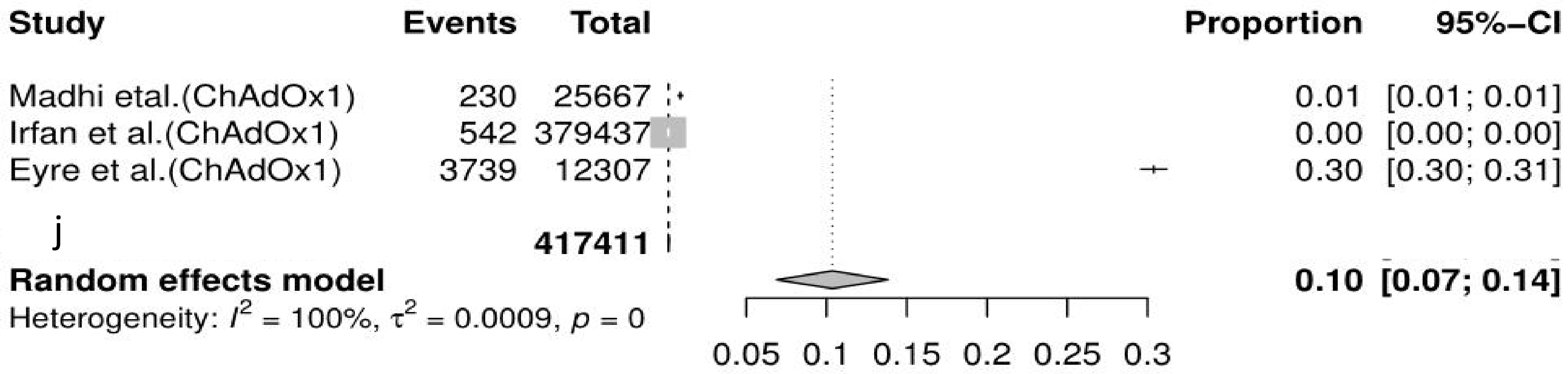
Study Events Total Proportion 95%−CI
Madhi etal.(ChAdOx1) 230 25667 0.01 [0.01; 0.01]
Irfan et al.(ChAdOx1) 542 379437 0.00 [0.00; 0.00]
Eyre et al.(ChAdOx1) 3739 12307 0.30 [0.30; 0.31]
j
417411
Random effects model 0.10 [0.07; 0.14]
Heterogeneity: I^2 = 100%, τ^2 = 0.0009, p = 0
0.05 0.1 0.15 0.2 0.25 0.3

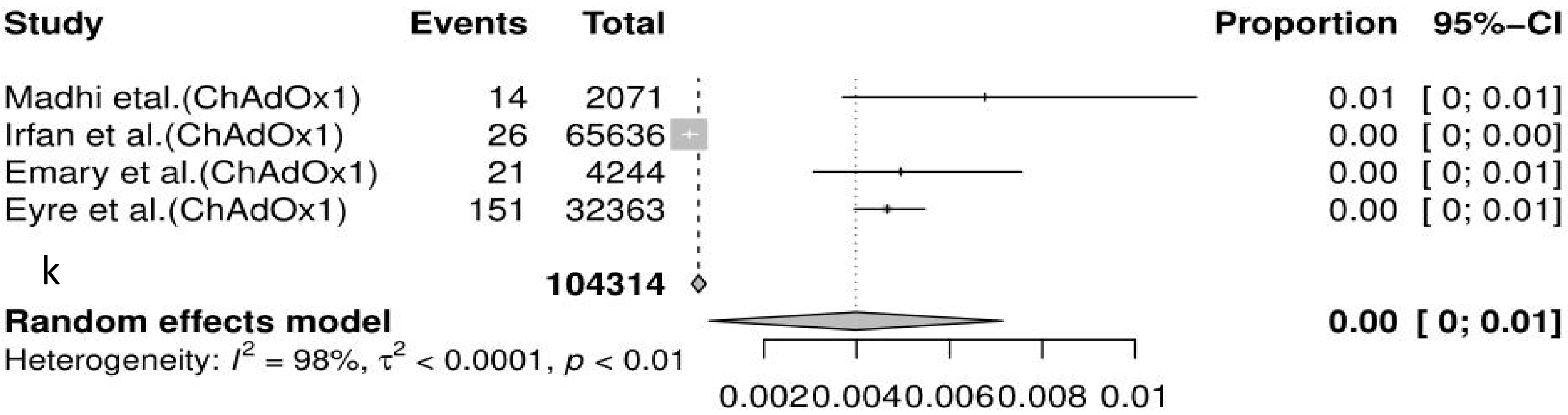
Study Events Total Proportion 95%−CI
Madhi etal.(ChAdOx1) 14 2071 0.01 [0; 0.01]
Irfan et al.(ChAdOx1) 26 65636 0.00 [0; 0.00]
Emary et al.(ChAdOx1) 21 4244 0.00 [0; 0.01]
Eyre et al.(ChAdOx1) 151 32363 0.00 [0; 0.01]
k
104314
Random effects model 0.00 [0; 0.01]
Heterogeneity: I^2 = 98%, τ^2 < 0.0001, p < 0.01
0.0020.0040.0060.008 0.01

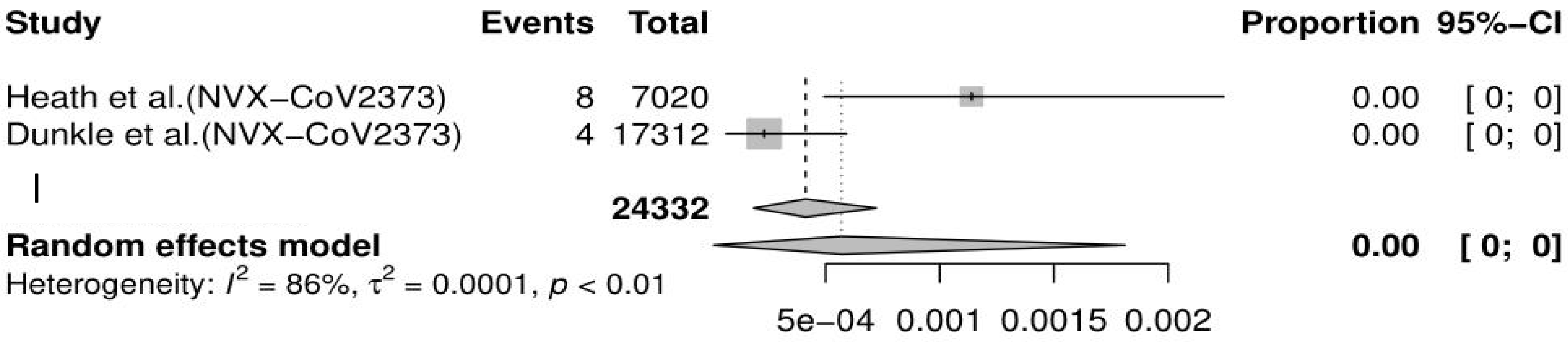
Study Events Total Proportion 95%−CI
Heath et al.(NVX−CoV2373) 8 7020 0.00 [0; 0]
Dunkle et al.(NVX−CoV2373) 4 17312 0.00 [0; 0]
l
24332
Random effects model 0.00 [0; 0]
Heterogeneity: I^2 = 86%, τ^2 = 0.0001, p < 0.01
5e−04 0.001 0.0015 0.002

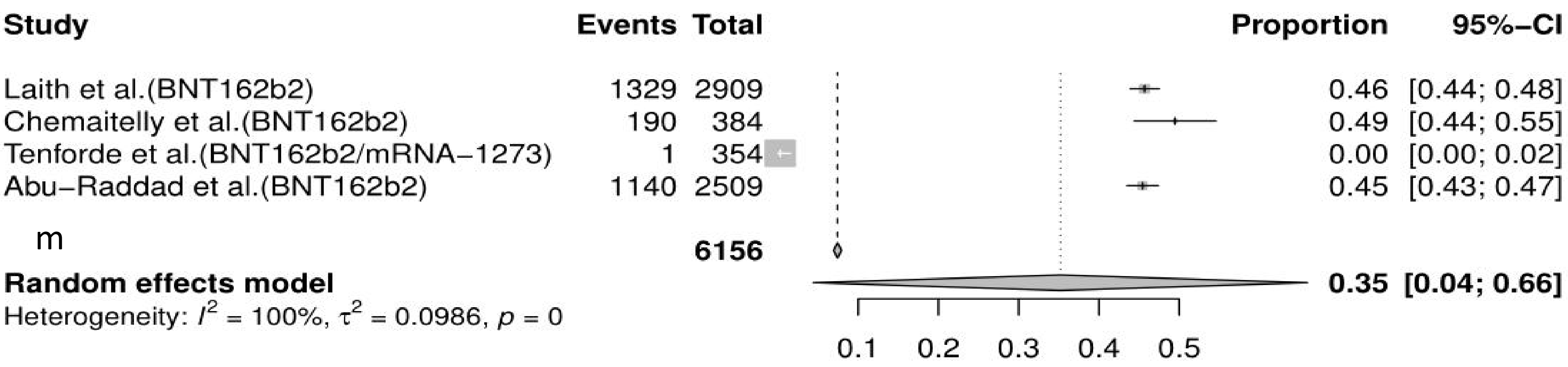
Study
Events Total
Proportion 95%−CI
Laith et al.(BNT162b2) 1329 2909 0.46 [0.44; 0.48]
Chemaitelly et al.(BNT162b2) 190 384 0.49 [0.44; 0.55]
Tenforde et al.(BNT162b2/mRNA−1273) 1 354 0.00 [0.00; 0.02]
Abu−Raddad et al.(BNT162b2) 1140 2509 0.45 [0.43; 0.47]
m
6156
Random effects model 0.35 [0.04; 0.66]
Heterogeneity: I^2 = 100%, τ^2 = 0.0986, p = 0
0.1 0.2 0.3 0.4 0.5

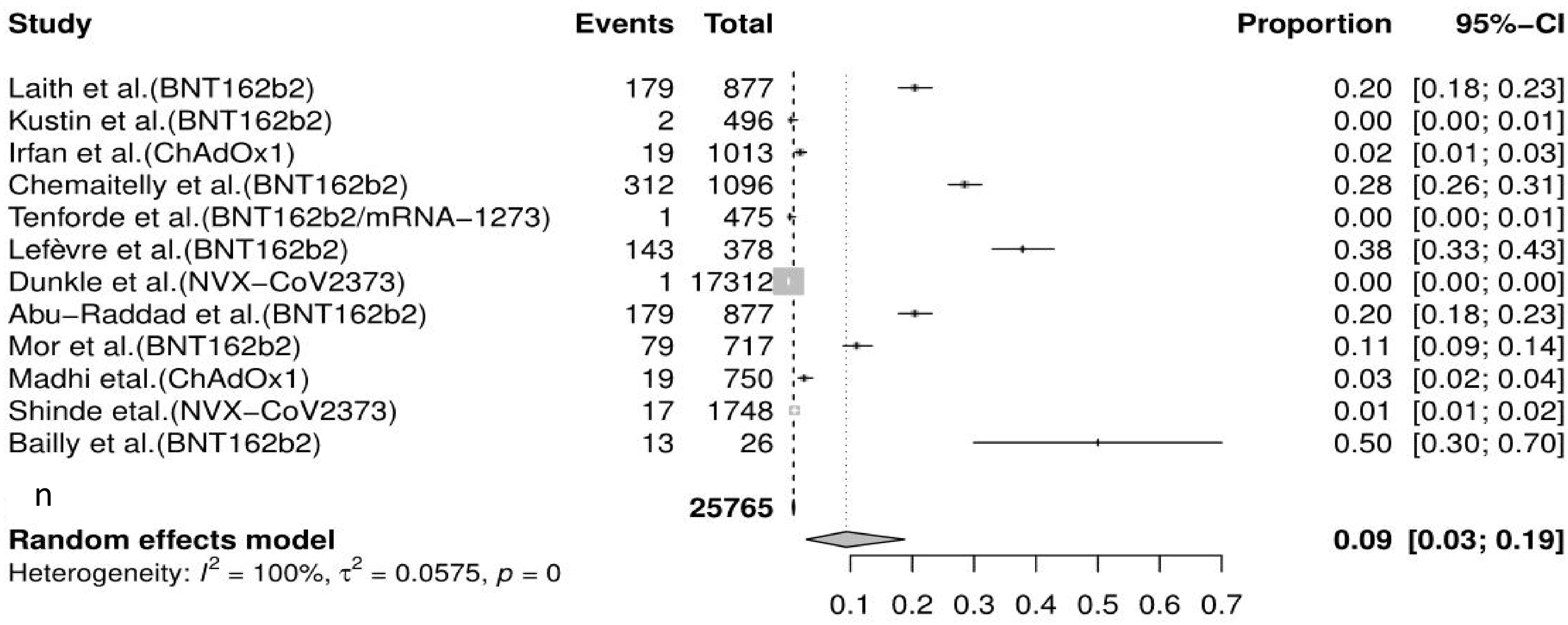
Study
Events Total
Proportion 95%−CI
Laith et al.(BNT162b2) 179 877 0.20 [0.18; 0.23]
Kustin et al.(BNT162b2) 2 496 0.00 [0.00; 0.01]
Irfan et al.(ChAdOx1) 19 1013 0.02 [0.01; 0.03]
Chemaitelly et al.(BNT162b2) 312 1096 0.28 [0.26; 0.31]
Tenforde et al.(BNT162b2/mRNA−1273) 1 475 0.00 [0.00; 0.01]
Lefèvre et al.(BNT162b2) 143 378 0.38 [0.33; 0.43]
Dunkle et al.(NVX−CoV2373) 1 17312 0.00 [0.00; 0.00]
Abu−Raddad et al.(BNT162b2) 179 877 0.20 [0.18; 0.23]
Mor et al.(BNT162b2) 79 717 0.11 [0.09; 0.14]
Madhi etal.(ChAdOx1) 19 750 0.03 [0.02; 0.04]
Shinde etal.(NVX−CoV2373) 17 1748 0.01 [0.01; 0.02]
Bailly et al.(BNT162b2) 13 26 0.50 [0.30; 0.70]
n
25765
Random effects model 0.09 [0.03; 0.19]
Heterogeneity: I^2 = 100%, τ^2 = 0.0575, p = 0
0.1 0.2 0.3 0.4 0.5 0.6 0.7

o

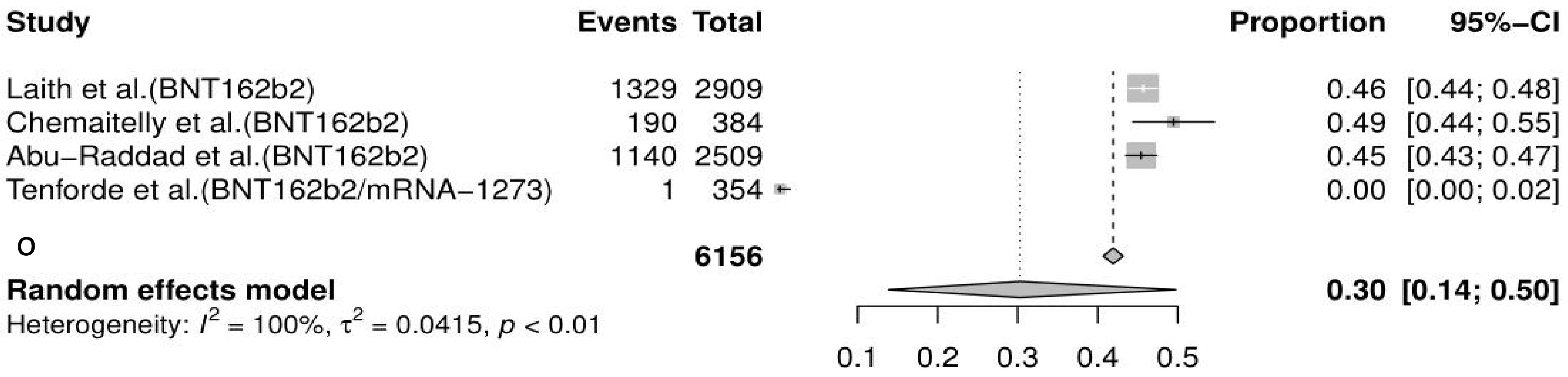
Study
Events
Total
Proportion
95%-CI
Laith et al.(BNT162b2) 1329 2909 0.46 [0.44; 0.48]
Chemaitelly et al.(BNT162b2) 190 384 0.49 [0.44; 0.55]
Abu−Raddad et al.(BNT162b2) 1140 2509 0.45 [0.43; 0.47]
Tenforde et al.(BNT162b2/mRNA−1273) 1 354 0.00 [0.00; 0.02]
6156
Random effects model 0.30 [0.14; 0.50]
Heterogeneity: $I^2 = 100\%$, $\tau^2 = 0.0415$, $p < 0.01$
0.1 0.2 0.3 0.4 0.5

p

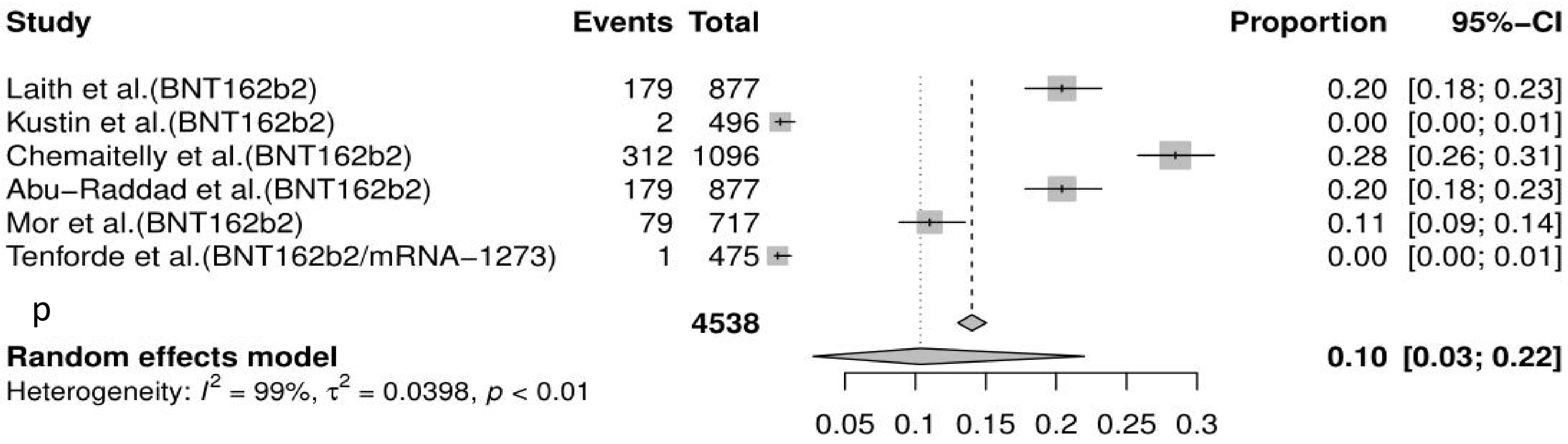
Study
Events
Total
Proportion
95%-CI
Laith et al.(BNT162b2) 179 877 0.20 [0.18; 0.23]
Kustin et al.(BNT162b2) 2 496 0.00 [0.00; 0.01]
Chemaitelly et al.(BNT162b2) 312 1096 0.28 [0.26; 0.31]
Abu−Raddad et al.(BNT162b2) 179 877 0.20 [0.18; 0.23]
Mor et al.(BNT162b2) 79 717 0.11 [0.09; 0.14]
Tenforde et al.(BNT162b2/mRNA−1273) 1 475 0.00 [0.00; 0.01]
4538
Random effects model 0.10 [0.03; 0.22]
Heterogeneity: $I^2 = 99\%$, $\tau^2 = 0.0398$, $p < 0.01$
0.05 0.1 0.15 0.2 0.25 0.3

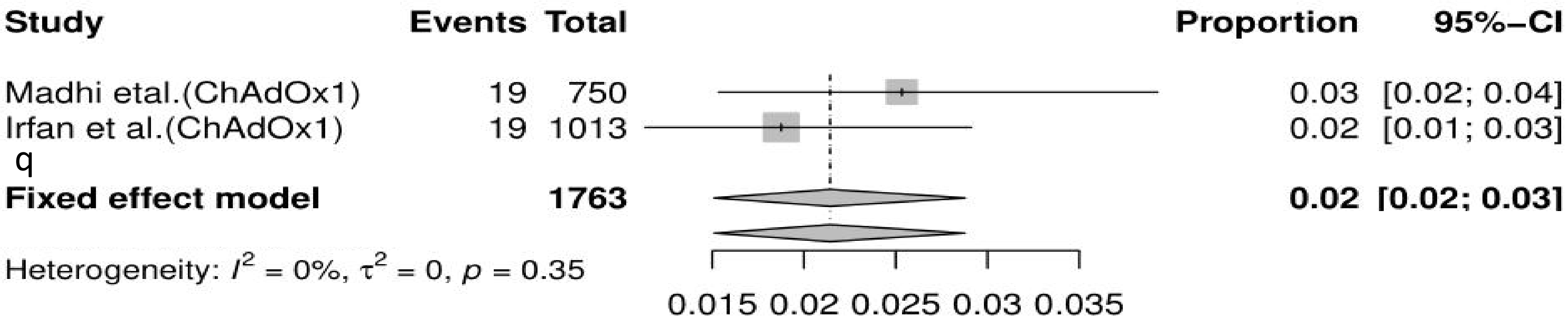
Study Events Total Proportion 95%−CI
Madhi etal.(ChAdOx1) 19 750 0.03 [0.02; 0.04]
Irfan et al.(ChAdOx1) 19 1013 0.02 [0.01; 0.03]
Fixed effect model 1763 0.02 [0.02; 0.03]
Heterogeneity: $I^2 = 0\%$, $\tau^2 = 0$, $p = 0.35$
0.015 0.02 0.025 0.03 0.035
q

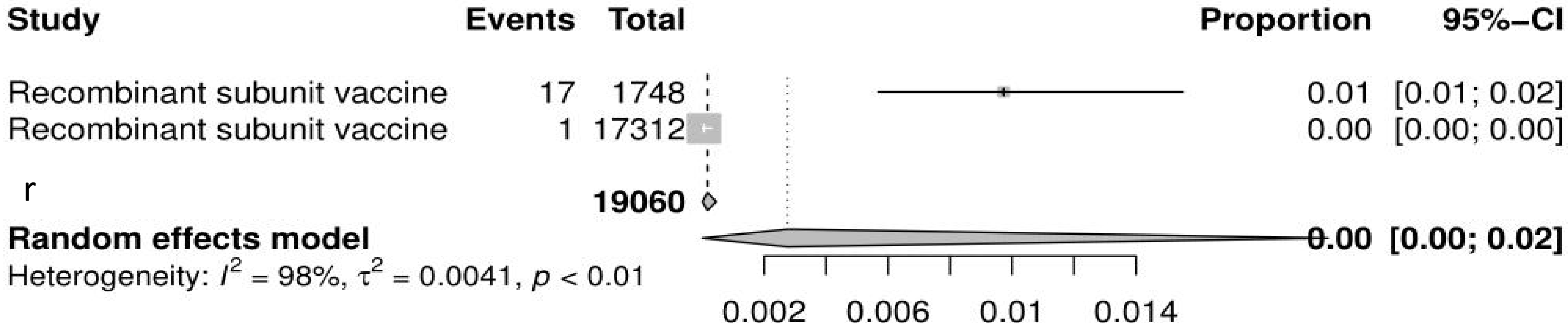
Study Events Total Proportion 95%−CI
Recombinant subunit vaccine 17 1748 0.01 [0.01; 0.02]
Recombinant subunit vaccine 1 17312 0.00 [0.00; 0.00]
19060
Random effects model 0.00 [0.00; 0.02]
Heterogeneity: $I^2 = 98\%$, $\tau^2 = 0.0041$, $p < 0.01$
0.002 0.006 0.01 0.014
r

s

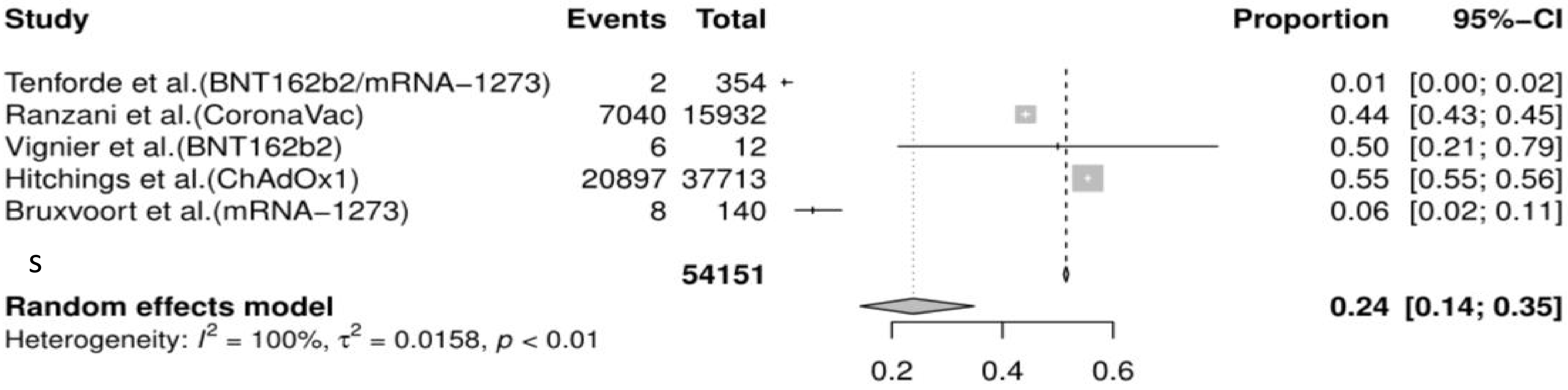
Study Events Total Proportion 95%−CI
Tenforde et al.(BNT162b2/mRNA−1273) 2 354 0.01 [0.00; 0.02]
Ranzani et al.(CoronaVac) 7040 15932 0.44 [0.43; 0.45]
Vignier et al.(BNT162b2) 6 12 0.50 [0.21; 0.79]
Hitchings et al.(ChAdOx1) 20897 37713 0.55 [0.55; 0.56]
Bruxvoort et al.(mRNA−1273) 8 140 0.06 [0.02; 0.11]
54151
Random effects model 0.24 [0.14; 0.35]
Heterogeneity: I^2 = 100%, τ^2 = 0.0158, p < 0.01
0.2
0.4
0.6

t

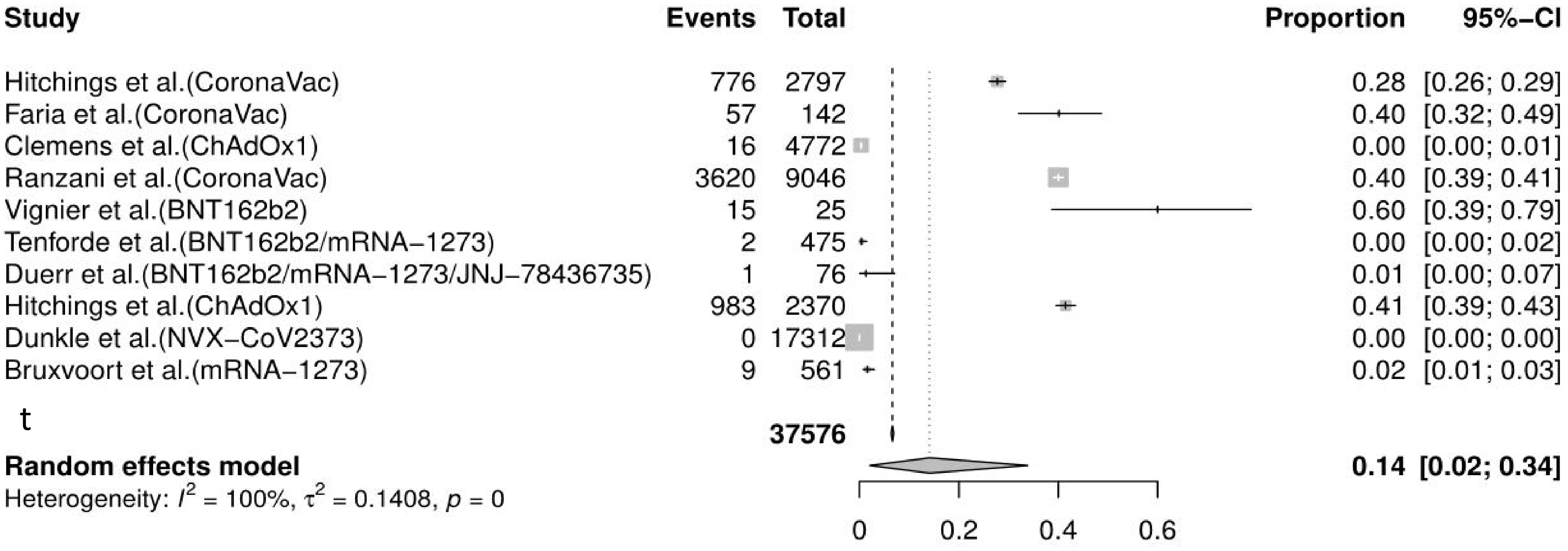
Study Events Total Proportion 95%−CI
Hitchings et al.(CoronaVac) 776 2797 0.28 [0.26; 0.29]
Faria et al.(CoronaVac) 57 142 0.40 [0.32; 0.49]
Clemens et al.(ChAdOx1) 16 4772 0.00 [0.00; 0.01]
Ranzani et al.(CoronaVac) 3620 9046 0.40 [0.39; 0.41]
Vignier et al.(BNT162b2) 15 25 0.60 [0.39; 0.79]
Tenforde et al.(BNT162b2/mRNA−1273) 2 475 0.00 [0.00; 0.02]
Duerr et al.(BNT162b2/mRNA−1273/JNJ−78436735) 1 76 0.01 [0.00; 0.07]
Hitchings et al.(ChAdOx1) 983 2370 0.41 [0.39; 0.43]
Dunkle et al.(NVX−CoV2373) 0 17312 0.00 [0.00; 0.00]
Bruxvoort et al.(mRNA−1273) 9 561 0.02 [0.01; 0.03]
37576
Random effects model 0.14 [0.02; 0.34]
Heterogeneity: I^2 = 100%, τ^2 = 0.1408, p = 0
0
0.2
0.4
0.6

u

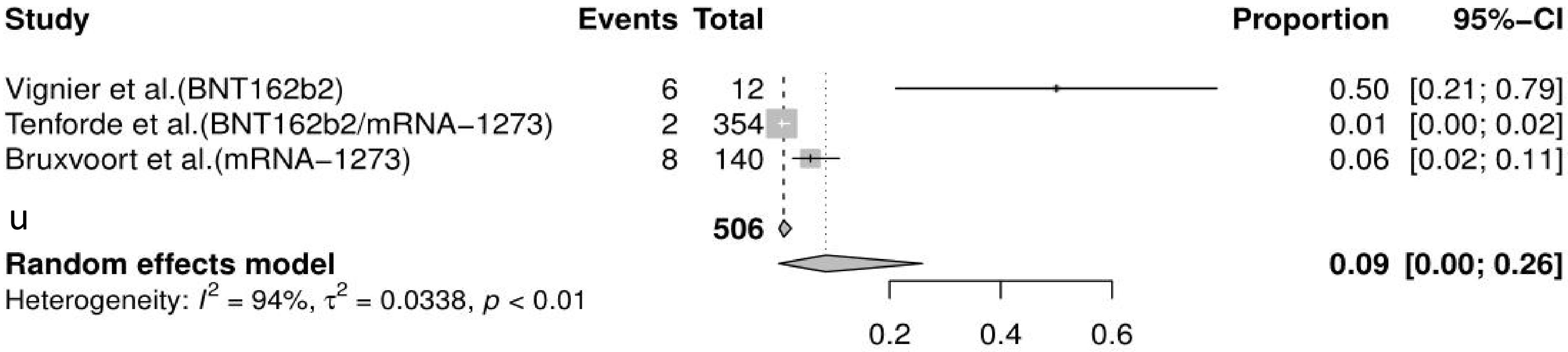
Study
Events
Total
Proportion
95%−CI
Vignier et al.(BNT162b2) 6 12 0.50 [0.21; 0.79]
Tenforde et al.(BNT162b2/mRNA−1273) 2 354 0.01 [0.00; 0.02]
Bruxvoort et al.(mRNA−1273) 8 140 0.06 [0.02; 0.11]
506
Random effects model 0.09 [0.00; 0.26]
Heterogeneity: $I^2 = 94\%$, $\tau^2 = 0.0338$, $p < 0.01$
0.2
0.4
0.6

v

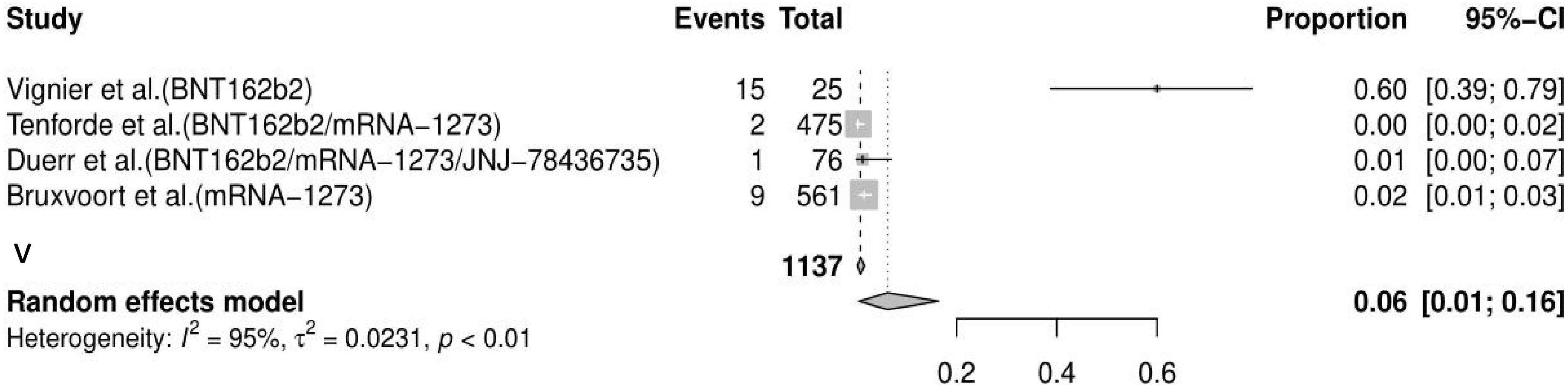
Study
Events
Total
Proportion
95%−CI
Vignier et al.(BNT162b2) 15 25 0.60 [0.39; 0.79]
Tenforde et al.(BNT162b2/mRNA−1273) 2 475 0.00 [0.00; 0.02]
Duerr et al.(BNT162b2/mRNA−1273/JNJ−78436735) 1 76 0.01 [0.00; 0.07]
Bruxvoort et al.(mRNA−1273) 9 561 0.02 [0.01; 0.03]
1137
Random effects model 0.06 [0.01; 0.16]
Heterogeneity: $I^2 = 95\%$, $\tau^2 = 0.0231$, $p < 0.01$
0.2
0.4
0.6

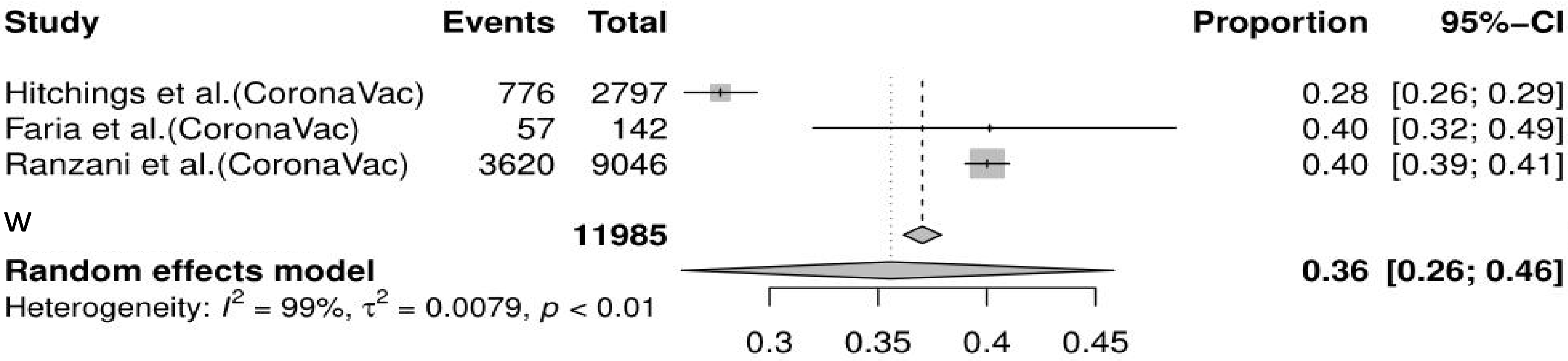
Study Events Total Proportion 95%-CI
Hitchings et al.(CoronaVac) 776 2797 0.28 [0.26; 0.29]
Faria et al.(CoronaVac) 57 142 0.40 [0.32; 0.49]
Ranzani et al.(CoronaVac) 3620 9046 0.40 [0.39; 0.41]
w
11985
Random effects model 0.36 [0.26; 0.46]
Heterogeneity: I^2 = 99%, τ^2 = 0.0079, p < 0.01
0.3 0.35 0.4 0.45

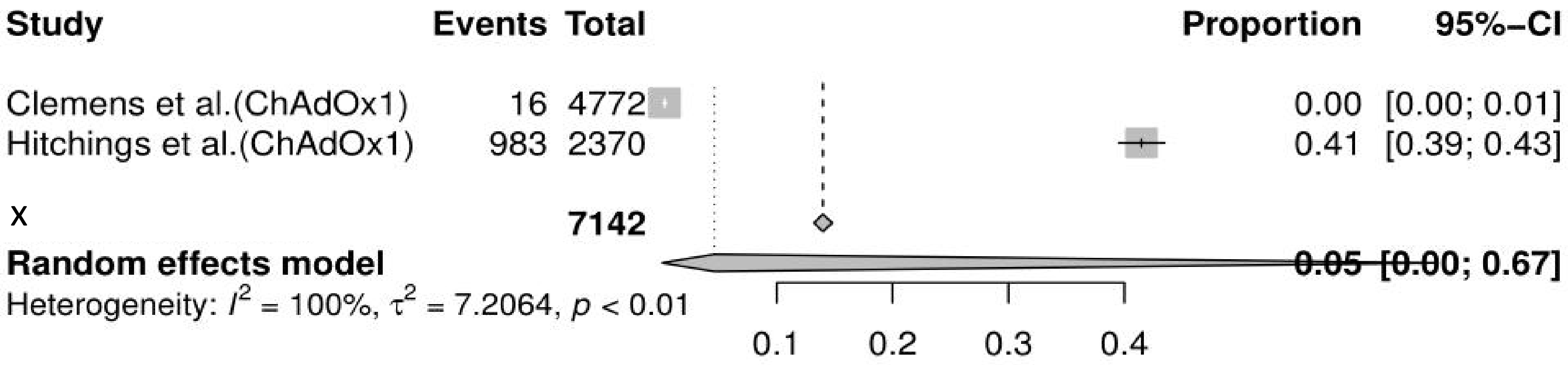
Study Events Total Proportion 95%-CI
Clemens et al.(ChAdOx1) 16 4772 0.00 [0.00; 0.01]
Hitchings et al.(ChAdOx1) 983 2370 0.41 [0.39; 0.43]
x
7142
Random effects model 0.05 [0.00; 0.67]
Heterogeneity: I^2 = 100%, τ^2 = 7.2064, p < 0.01
0.1 0.2 0.3 0.4

Study	Events	Total	Proportion	95%-CI
Bernal et al.(BNT162b2)	28	6412	0.00	[0.00; 0.01]
Bernal et al.(ChAdOx1)*	46	2071	0.02	[0.02; 0.03]
Grant et al.(BNT162b2)	2202	3927	0.56	[0.55; 0.58]
Grant et al.(ChAdOx1)*	153	247	0.62	[0.56; 0.68]
Tang et al. (BNT162b2)	998	6590	0.15	[0.14; 0.16]
Tang et al. (mRNA−1273)*	150	1718	0.09	[0.07; 0.10]
Tang et al. (BNT162b2/mRNA−1273)**	1174	8036	0.15	[0.14; 0.15]
Pouwels et al.(BNT162b2)	488	227907	0.00	[0.00; 0.00]
Pouwels et al.(ChAdOx1)*	1328	379985	0.00	[0.00; 0.00]
Yi et al.(BNT162b2)	24	56	0.43	[0.30; 0.57]
Chemaitelly et al.(BNT162b2)	650	1768	0.37	[0.35; 0.39]
Li et al.(CoronaVac)	12	109	0.11	[0.06; 0.18]
Williams et al(ChAdOx1)	18	30	0.60	[0.41; 0.77]
Desai et al.(BBV152)	186	449	0.41	[0.37; 0.46]
Bruxvoort et al.(mRNA−1273)	232	4820	0.05	[0.04; 0.05]
Reis et al.(BNT162b2)	75	46815	0.00	[0.00; 0.00]
Olson et al.(BNT162b2)	6	179	0.03	[0.01; 0.07]
y		**691119**		
**Random effects model**			**0.14**	**[0.11; 0.18]**

Heterogeneity: $I^2 = 100\%$, $\tau^2 = 0.0132$, $p = 0$

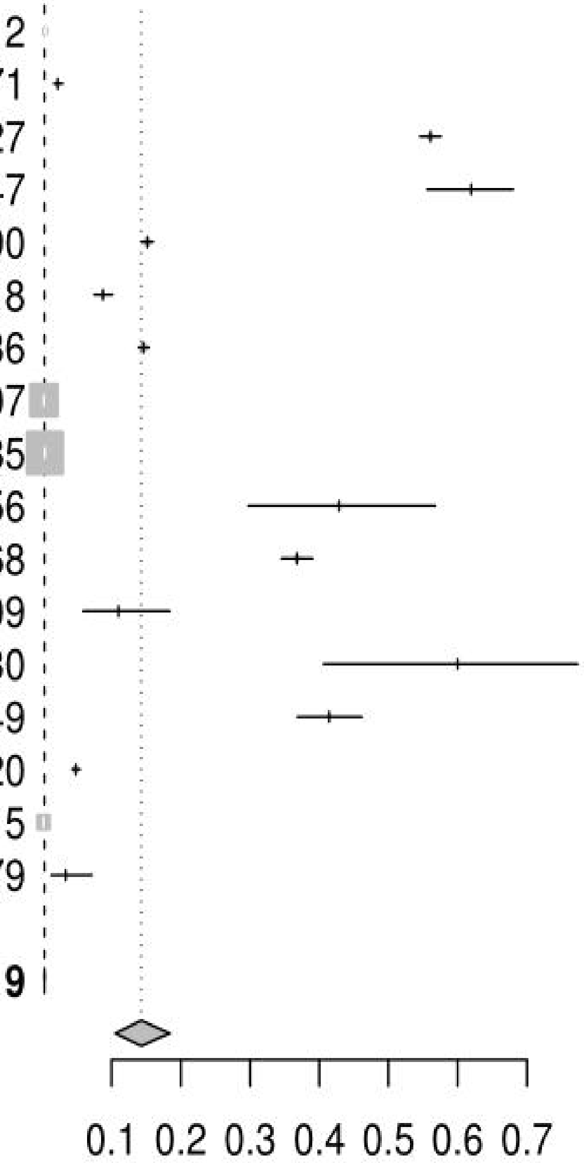

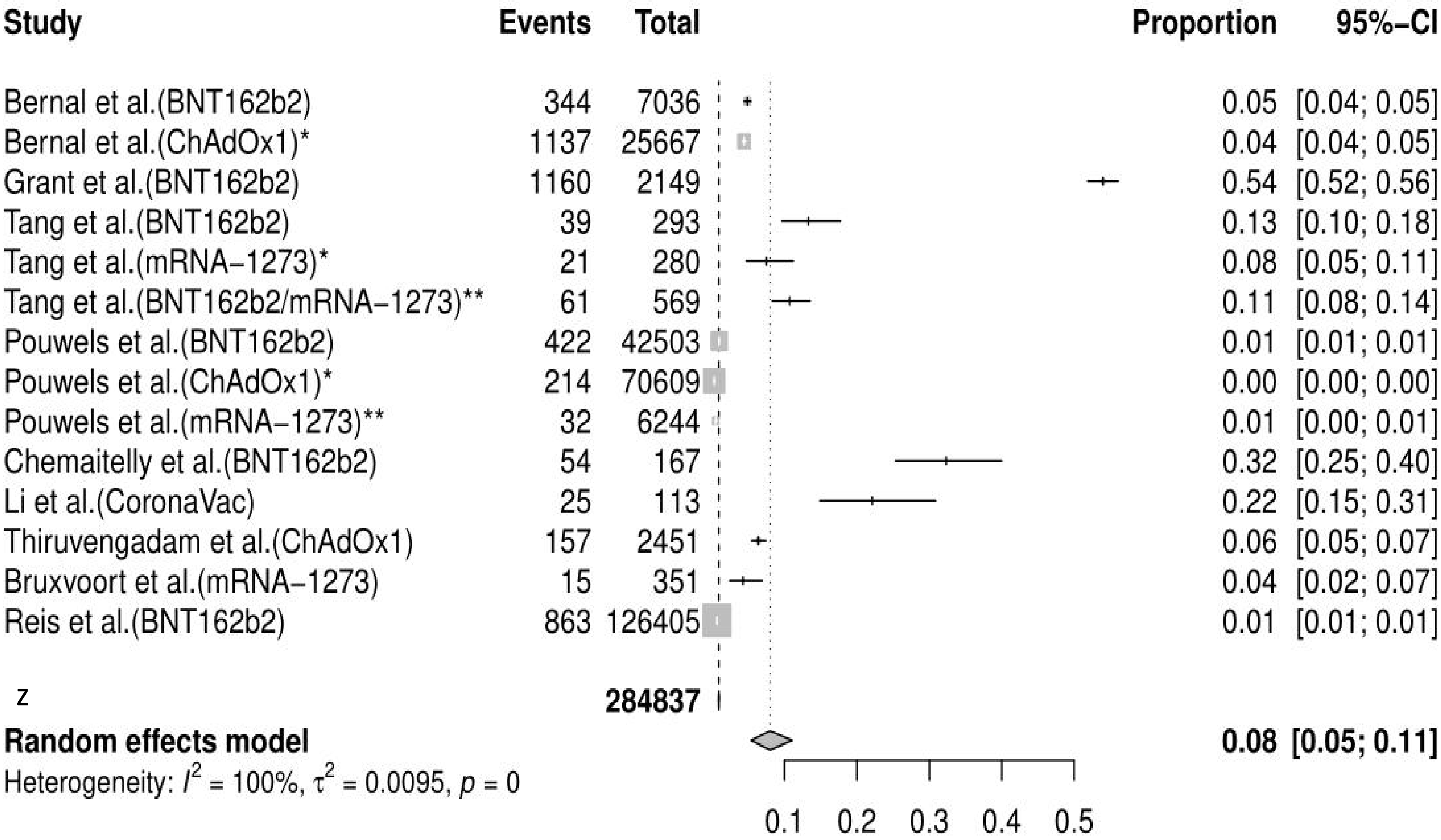
Study Events Total Proportion 95%−CI
Bernal et al.(BNT162b2) 344 7036 0.05 [0.04; 0.05]
Bernal et al.(ChAdOx1)* 1137 25667 0.04 [0.04; 0.05]
Grant et al.(BNT162b2) 1160 2149 0.54 [0.52; 0.56]
Tang et al.(BNT162b2) 39 293 0.13 [0.10; 0.18]
Tang et al.(mRNA−1273)* 21 280 0.08 [0.05; 0.11]
Tang et al.(BNT162b2/mRNA−1273)** 61 569 0.11 [0.08; 0.14]
Pouwels et al.(BNT162b2) 422 42503 0.01 [0.01; 0.01]
Pouwels et al.(ChAdOx1)* 214 70609 0.00 [0.00; 0.00]
Pouwels et al.(mRNA−1273)** 32 6244 0.01 [0.00; 0.01]
Chemaitelly et al.(BNT162b2) 54 167 0.32 [0.25; 0.40]
Li et al.(CoronaVac) 25 113 0.22 [0.15; 0.31]
Thiruvengadam et al.(ChAdOx1) 157 2451 0.06 [0.05; 0.07]
Bruxvoort et al.(mRNA−1273) 15 351 0.04 [0.02; 0.07]
Reis et al.(BNT162b2) 863 126405 0.01 [0.01; 0.01]
z
284837
Random effects model 0.08 [0.05; 0.11]
Heterogeneity: I² = 100%, τ² = 0.0095, p = 0
0.1 0.2 0.3 0.4 0.5

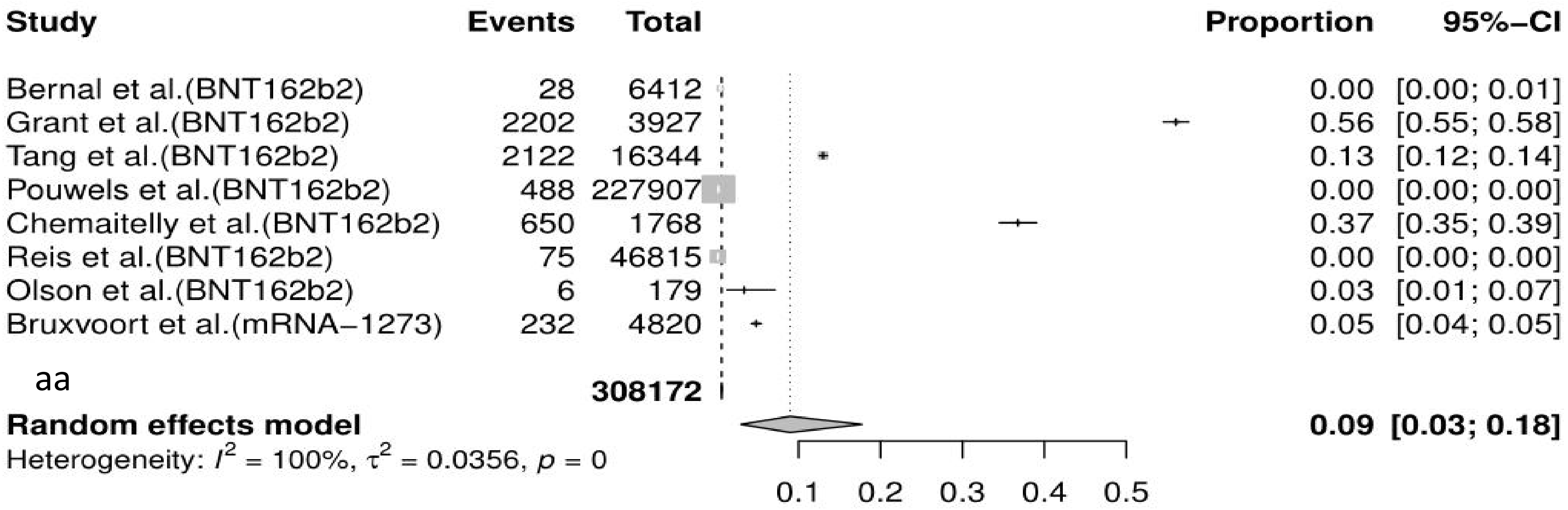
Study
Events
Total
Proportion
95%-CI
Bernal et al.(BNT162b2) 28 6412 0.00 [0.00; 0.01]
Grant et al.(BNT162b2) 2202 3927 0.56 [0.55; 0.58]
Tang et al.(BNT162b2) 2122 16344 0.13 [0.12; 0.14]
Pouwels et al.(BNT162b2) 488 227907 0.00 [0.00; 0.00]
Chemaitelly et al.(BNT162b2) 650 1768 0.37 [0.35; 0.39]
Reis et al.(BNT162b2) 75 46815 0.00 [0.00; 0.00]
Olson et al.(BNT162b2) 6 179 0.03 [0.01; 0.07]
Bruxvoort et al.(mRNA−1273) 232 4820 0.05 [0.04; 0.05]
aa
308172
Random effects model 0.09 [0.03; 0.18]
Heterogeneity: I^2 = 100%, τ^2 = 0.0356, p = 0
0.1
0.2
0.3
0.4
0.5

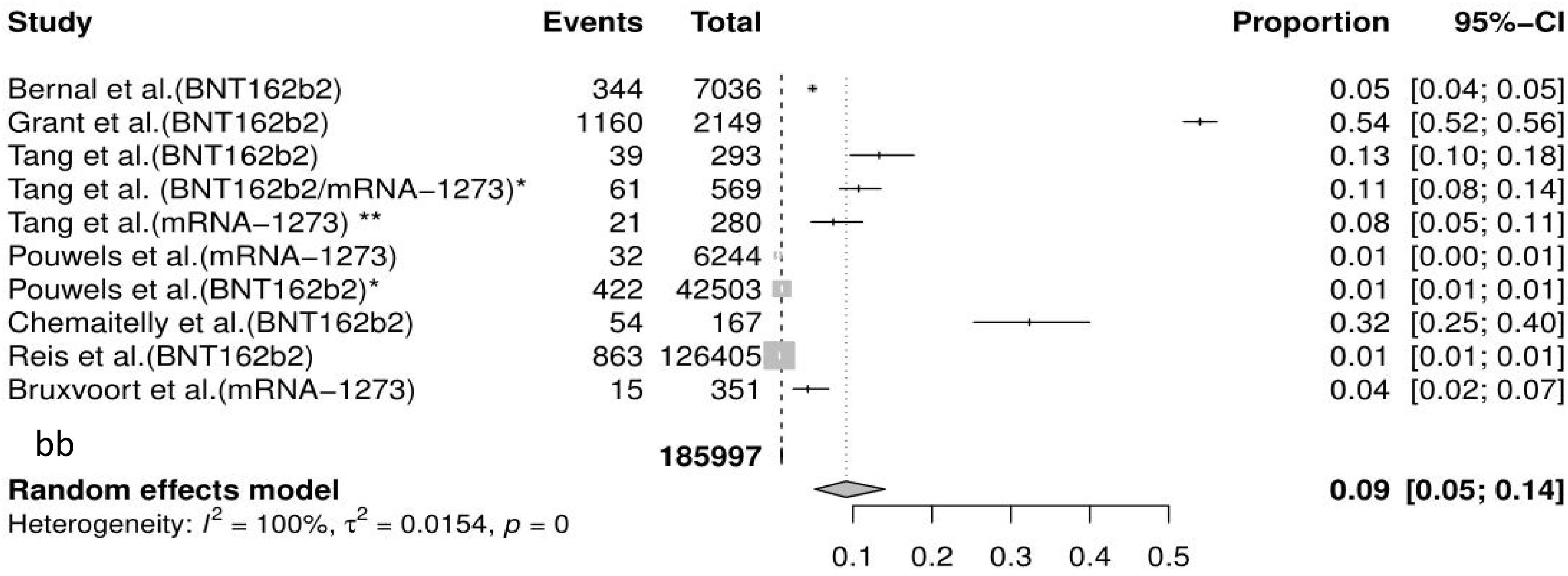
Study
Events
Total
Proportion
95%-CI
Bernal et al.(BNT162b2) 344 7036 0.05 [0.04; 0.05]
Grant et al.(BNT162b2) 1160 2149 0.54 [0.52; 0.56]
Tang et al.(BNT162b2) 39 293 0.13 [0.10; 0.18]
Tang et al. (BNT162b2/mRNA−1273)* 61 569 0.11 [0.08; 0.14]
Tang et al.(mRNA−1273) ** 21 280 0.08 [0.05; 0.11]
Pouwels et al.(mRNA−1273) 32 6244 0.01 [0.00; 0.01]
Pouwels et al.(BNT162b2)* 422 42503 0.01 [0.01; 0.01]
Chemaitelly et al.(BNT162b2) 54 167 0.32 [0.25; 0.40]
Reis et al.(BNT162b2) 863 126405 0.01 [0.01; 0.01]
Bruxvoort et al.(mRNA−1273) 15 351 0.04 [0.02; 0.07]
bb
185997
Random effects model 0.09 [0.05; 0.14]
Heterogeneity: I^2 = 100%, τ^2 = 0.0154, p = 0
0.1
0.2
0.3
0.4
0.5

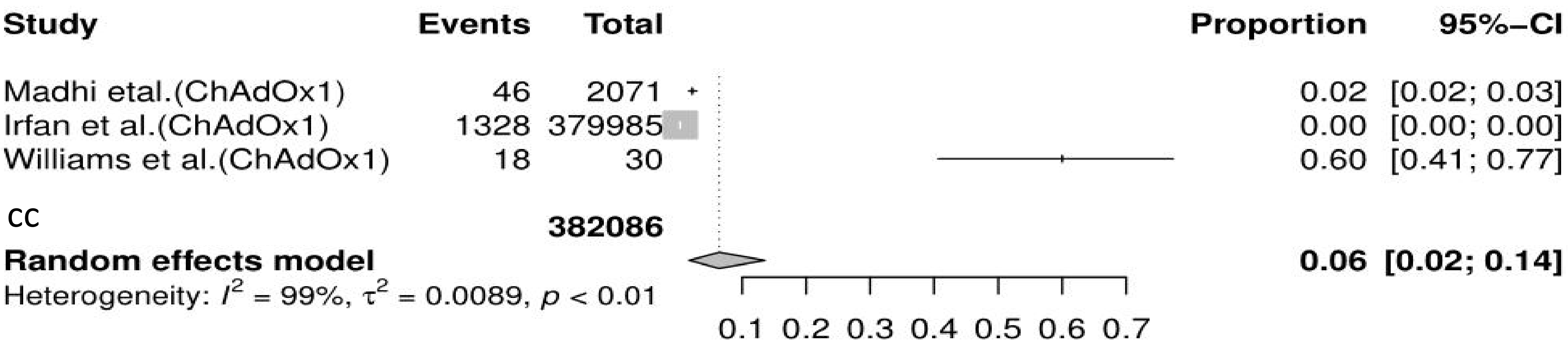
Study Events Total Proportion 95%–CI
Madhi etal.(ChAdOx1) 46 2071 0.02 [0.02; 0.03]
Irfan et al.(ChAdOx1) 1328 379985 0.00 [0.00; 0.00]
Williams et al.(ChAdOx1) 18 30 0.60 [0.41; 0.77]
cc
382086
Random effects model 0.06 [0.02; 0.14]
Heterogeneity: I^2 = 99%, τ^2 = 0.0089, p < 0.01
0.1 0.2 0.3 0.4 0.5 0.6 0.7

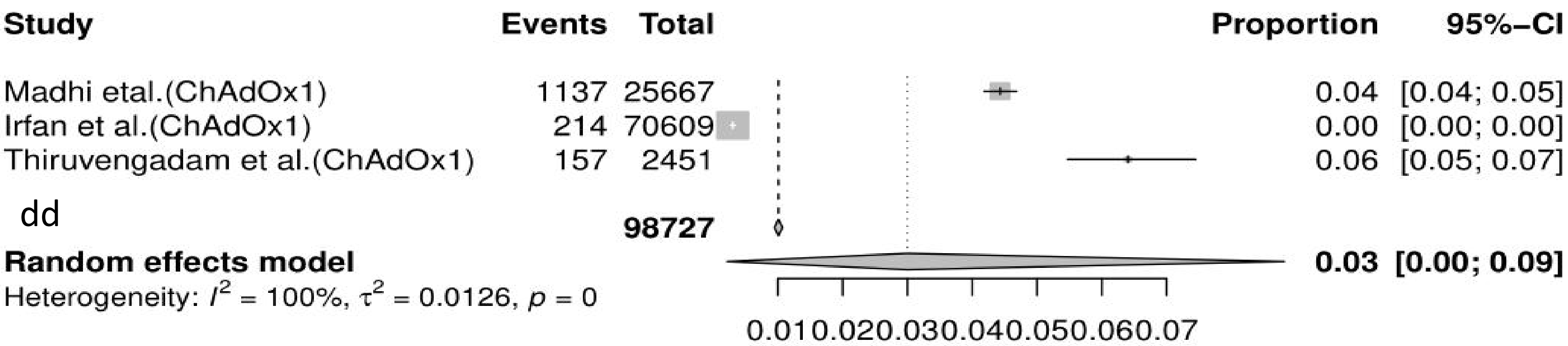
Study Events Total Proportion 95%–CI
Madhi etal.(ChAdOx1) 1137 25667 0.04 [0.04; 0.05]
Irfan et al.(ChAdOx1) 214 70609 0.00 [0.00; 0.00]
Thiruvengadam et al.(ChAdOx1) 157 2451 0.06 [0.05; 0.07]
dd
98727
Random effects model 0.03 [0.00; 0.09]
Heterogeneity: I^2 = 100%, τ^2 = 0.0126, p = 0
0.010.020.030.040.050.060.07

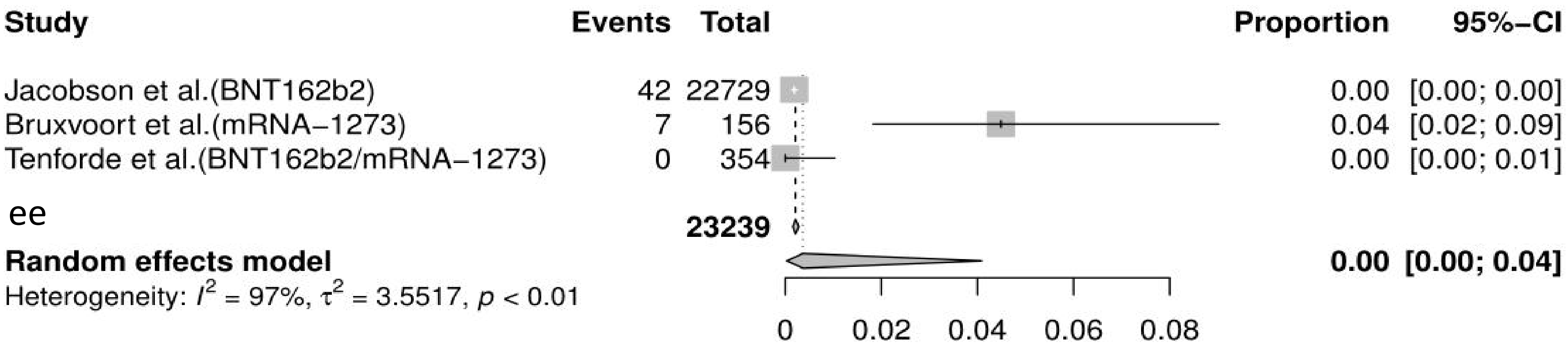
Study
Events
Total
Proportion
95%-CI
Jacobson et al.(BNT162b2) 42 22729 0.00 [0.00; 0.00]
Bruxvoort et al.(mRNA–1273) 7 156 0.04 [0.02; 0.09]
Tenforde et al.(BNT162b2/mRNA–1273) 0 354 0.00 [0.00; 0.01]
ee
23239
Random effects model 0.00 [0.00; 0.04]
Heterogeneity: I^2 = 97%, τ^2 = 3.5517, p < 0.01
0
0.02
0.04
0.06
0.08

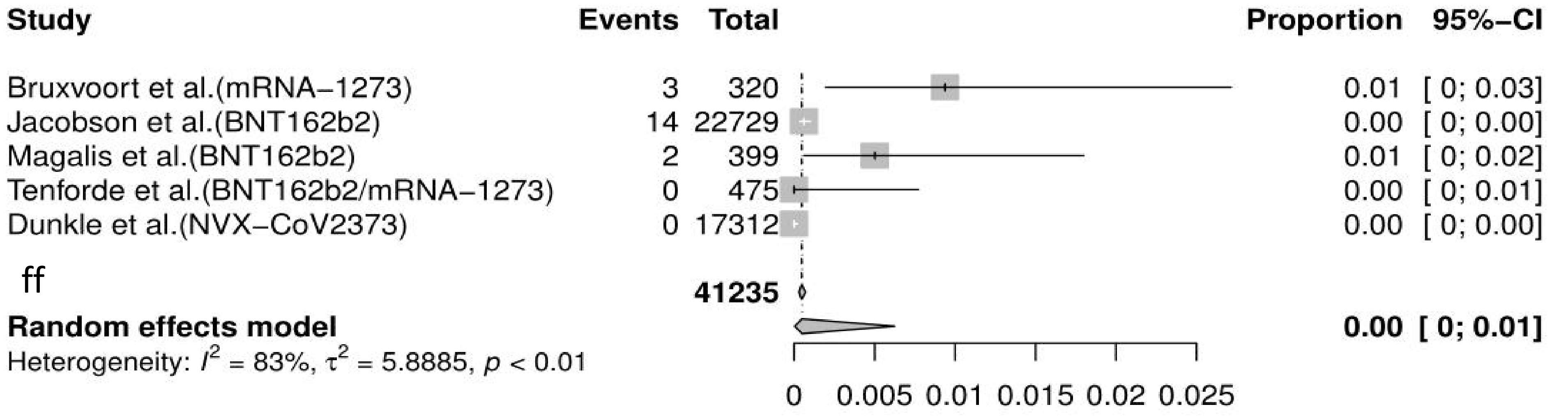
Study
Events
Total
Proportion
95%-CI
Bruxvoort et al.(mRNA–1273) 3 320 0.01 [0; 0.03]
Jacobson et al.(BNT162b2) 14 22729 0.00 [0; 0.00]
Magalis et al.(BNT162b2) 2 399 0.01 [0; 0.02]
Tenforde et al.(BNT162b2/mRNA–1273) 0 475 0.00 [0; 0.01]
Dunkle et al.(NVX–CoV2373) 0 17312 0.00 [0; 0.00]
ff
41235
Random effects model 0.00 [0; 0.01]
Heterogeneity: I^2 = 83%, τ^2 = 5.8885, p < 0.01
0
0.005
0.01
0.015
0.02
0.025

gg

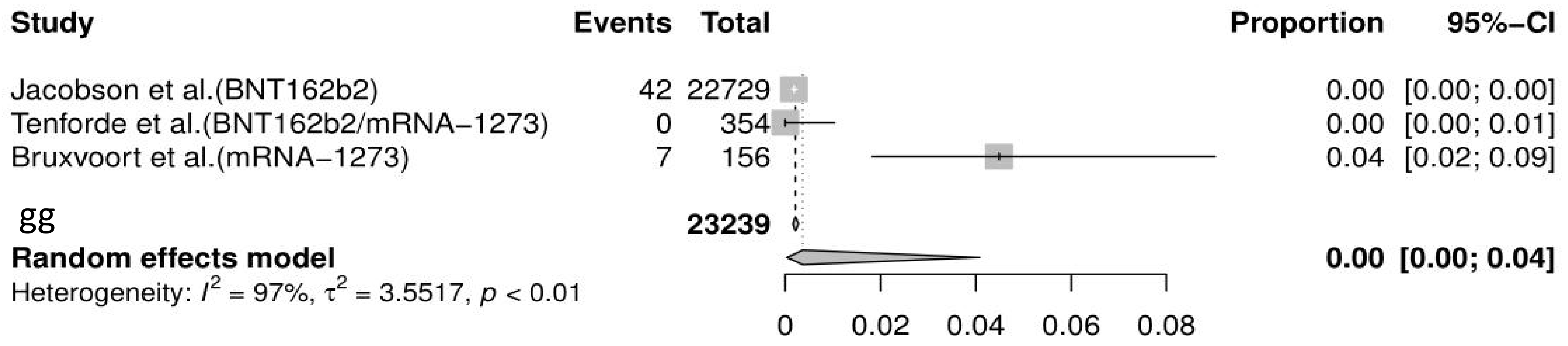
Study
Events
Total
Proportion
95%-CI
Jacobson et al.(BNT162b2) 42 22729 0.00 [0.00; 0.00]
Tenforde et al.(BNT162b2/mRNA-1273) 0 354 0.00 [0.00; 0.01]
Bruxvoort et al.(mRNA-1273) 7 156 0.04 [0.02; 0.09]
23239
Random effects model 0.00 [0.00; 0.04]
Heterogeneity: $I^2 = 97\%$, $\tau^2 = 3.5517$, $p < 0.01$
0
0.02
0.04
0.06
0.08

hh

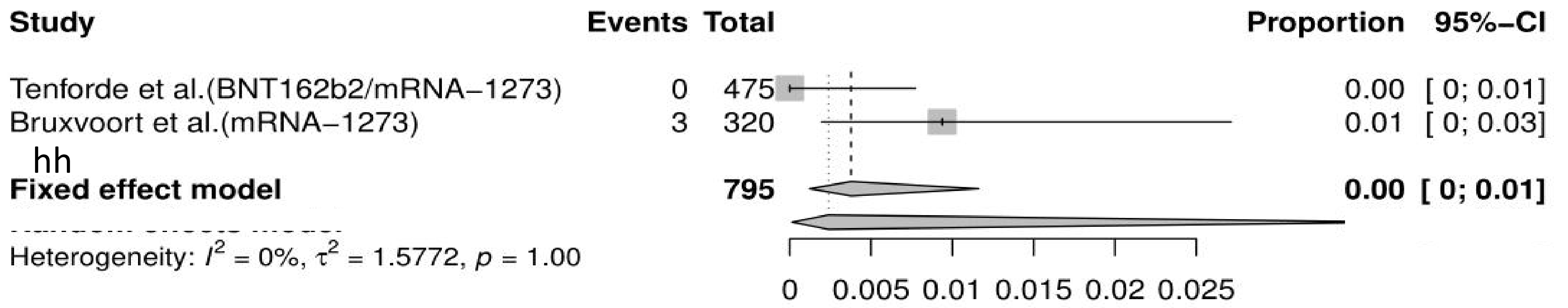
Study
Events
Total
Proportion
95%-CI
Tenforde et al.(BNT162b2/mRNA-1273) 0 475 0.00 [0; 0.01]
Bruxvoort et al.(mRNA-1273) 3 320 0.01 [0; 0.03]
Fixed effect model 795 0.00 [0; 0.01]
Heterogeneity: $I^2 = 0\%$, $\tau^2 = 1.5772$, $p = 1.00$
0
0.005
0.01
0.015
0.02
0.025

ii

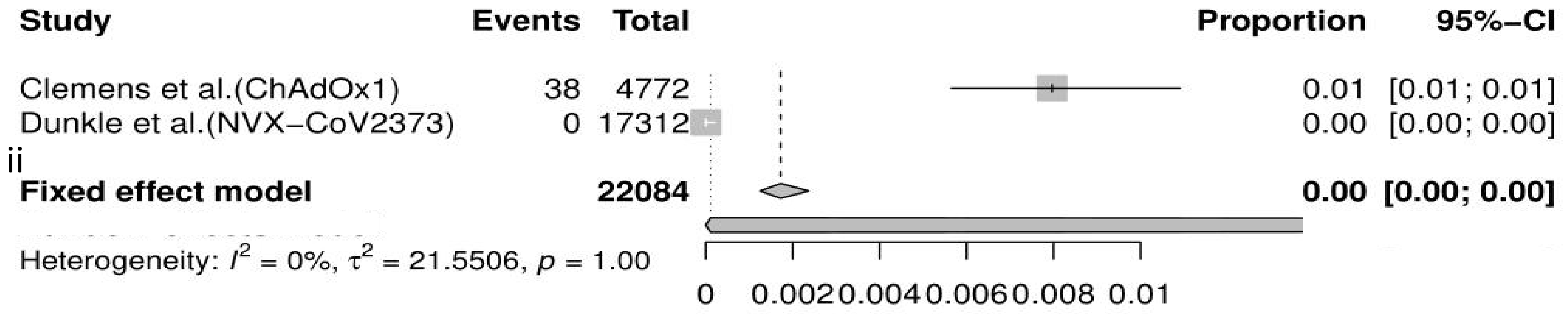
Study
Events
Total
Proportion
95%-CI
Clemens et al.(ChAdOx1) 38 4772 0.01 [0.01; 0.01]
Dunkle et al.(NVX-CoV2373) 0 17312 0.00 [0.00; 0.00]
Fixed effect model 22084 0.00 [0.00; 0.00]
Heterogeneity: $I^2 = 0\%$, $\tau^2 = 21.5506$, $p = 1.00$
0
0.002
0.004
0.006
0.008
0.01

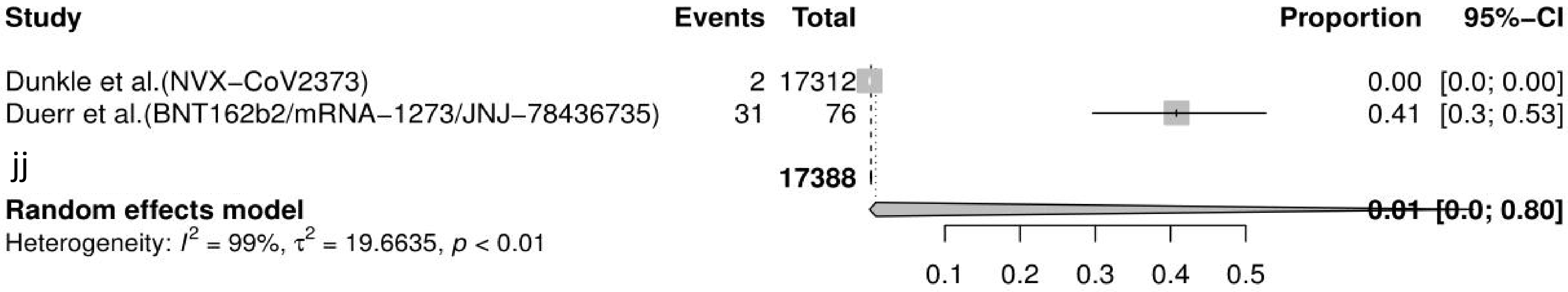
Study
Events
Total
Proportion
95%−CI
Dunkle et al.(NVX−CoV2373) 2 17312 0.00 [0.0; 0.00]
Duerr et al.(BNT162b2/mRNA−1273/JNJ−78436735) 31 76 0.41 [0.3; 0.53]
jj
17388
Random effects model 0.01 [0.0; 0.80]
Heterogeneity: I^2 = 99%, τ^2 = 19.6635, p < 0.01
0.1
0.2
0.3
0.4
0.5

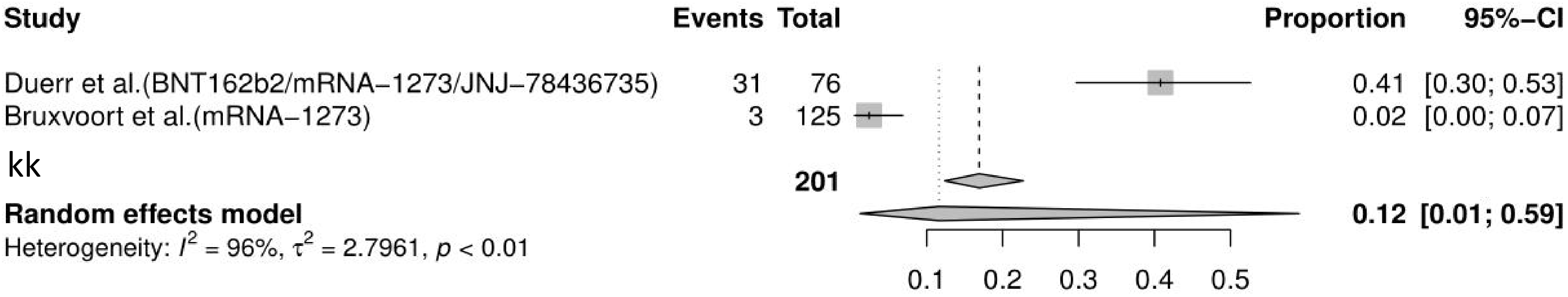
Study
Events
Total
Proportion
95%−CI
Duerr et al.(BNT162b2/mRNA−1273/JNJ−78436735) 31 76 0.41 [0.30; 0.53]
Bruxvoort et al.(mRNA−1273) 3 125 0.02 [0.00; 0.07]
kk
201
Random effects model 0.12 [0.01; 0.59]
Heterogeneity: I^2 = 96%, τ^2 = 2.7961, p < 0.01
0.1
0.2
0.3
0.4
0.5
