# Supplementary material for: Real-Word Effectiveness of Global COVID-19 Vaccines Against SARS-CoV-2 Variants: A Systematic Review and Meta-Analysis
Source: Front Med (Lausanne). 2022 May 19;9:820544. doi: 10.3389/fmed.2022.820544 (PMC9160927; doi:10.3389/fmed.2022.820544)
Supplement: Supplementary Figure 2 — Subgroup analysis for the pooled efficacy of mRNA vaccine against variants post first dose (A), mRNA vaccine against variants post second dose (B), viral vector vaccine against variants post second dose (C), vaccines against the B.1.1.7 (Alpha) variant post first dose (D),vaccines against the B.1.1.7 (Alpha) variant post second dose (E), mRNA vaccine against the B.1.1.7 (Alpha) variant post first dose (F), mRNA vaccine against the B.1.1.7 (Alpha) variant post second dose (G), subunit vaccine against the B.1.1.7 (Alpha) variant post second dose (H), viral vector vaccine against the B.1.1.7 (Alpha) variant post second dose (I), vaccines against the B.1.351 (Beta) variant post first dose (J), vaccines against the B.1.351 (Beta) variant post second dose (K), mRNA vaccine against the B.1.351 (Beta) variant post first dose (L), mRNA vaccine against the B.1.351 (Beta) variant post second dose (M), vaccines against the P.1 (Gamma) variant post first dose (N), vaccines against the P.1 (Gamma) variant post second dose (O), mRNA vaccine against the P.1 (Gamma) variant post first dose (P), mRNA vaccine against the P.1 (Gamma) variant post second dose (Q), viral vector vaccine against the P.1 (Gamma) variant post second dose (R), vaccines against the B.1.617.2 (Delta) variant post first dose (S), vaccines against the B.1.617.2 (Delta) variant post second dose (T), mRNA vaccine against the B.1.617.2 (Delta) variant post second dose (U), viral vector vaccine against the B.1.617.2 (Delta) variant post first dose (V), viral vector vaccine against the B.1.617.2 (Delta) variant post second dose (W), vaccines against the B.1.427 (Epsilon) variant post first dose (X), vaccines against the B.1.427 (Epsilon) variant post second dose (Y), mRNA vaccine against the B.1.427 (Epsilon) variant post first dose (Z), mRNA vaccine against the B.1.427 (Epsilon) variant post second dose (AA), vaccines against the P.2 (Zeta) variant post second dose (BB), vaccines against the B.1.526 (Iota) variant po [file Data_Sheet_2.pdf]

a

Study	Experimental Events	Experimental Total	Control Events	Control Total	OR	95%-CI
Laith et al.(BNT162b2)	2284	5042	38252	75904	0.82	[0.77; 0.86]
Bernal et al.(BNT162b2)	399	7036	5586	58253	0.57	[0.51; 0.63]
Hall et al.(BNT162b2)	71	87278	977	710587	0.59	[0.46; 0.75]
		**99356**		**844744**		
**Random effects model**					**0.65**	**[0.49; 0.87]**

Heterogeneity: $I^2 = 95\%$, $\tau^2 = 0.0583$, $p < 0.01$

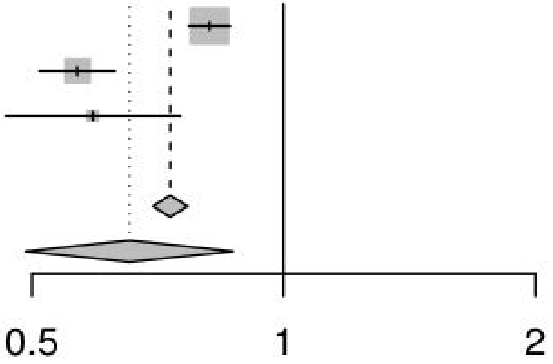

b

Study	Experimental Events	Experimental Total	Control Events	Control Total	OR	95%-CI
Laith et al.(BNT162b2)	229	1392	35750	70506	0.19	[0.17; 0.22]
Bernal et al.(BNT162b2)	41	6412	5586	58253	0.06	[0.04; 0.08]
Hall et al.(BNT162b2)	9	20978	977	710587	0.31	[0.16; 0.60]
Haas et al.(BNT162b2)	3442	4714932	103833	1823979	0.01	[0.01; 0.01]
Bailly et al.(BNT162b2)	13	26	5	5	0.09	[0.00; 1.81]
Cavanaugh et al.(BNT162b2)	22	127	22	62	0.38	[0.19; 0.76]
Kustin et al.(BNT162b2)	346	496	364	496	0.84	[0.63; 1.10]
		**4744363**		**2663888**		
**Random effects model**					**0.15**	**[0.03; 0.72]**

Heterogeneity: $I^2 = 100\%$, $\tau^2 = 4.3658$, $p = 0$

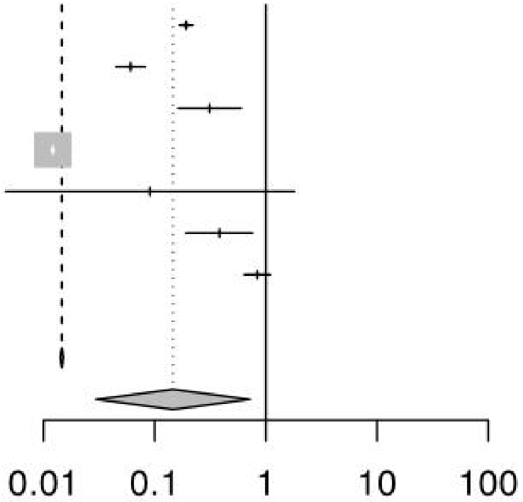

c

Study	Experimental Events	Experimental Total	Control Events	Control Total	OR	95%-CI
Madhi etal.(ChAdOx1)	60	2071	5586	58253	0.28	[0.22; 0.36]
Emary et al.(ChAdOx1)	27	4244	117	4290	0.23	[0.15; 0.35]
Irfan et al.(ChAdOx1)	19	1013	23	1013	0.82	[0.45; 1.52]
Clemens et al.(ChAdOx1)	77	4772	222	4661	0.33	[0.25; 0.43]
		**12100**		**68217**		
**Random effects model**					**0.34**	**[0.23; 0.49]**

Heterogeneity: $I^2 = 76\%$, $\tau^2 = 0.0984$, $p < 0.01$

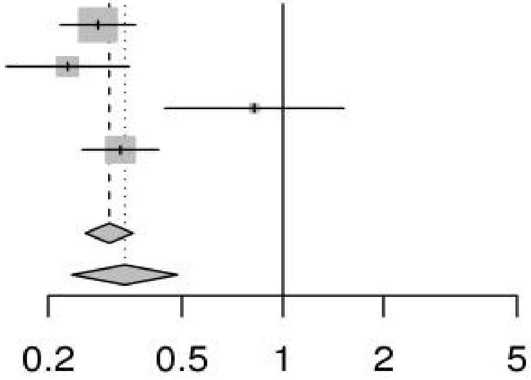

d

Study	Experimental Events	Experimental Total	Control Events	Control Total	OR	95%-CI
Laith et al.(BNT162b2)	892	2133	18075	35801	0.70	[0.65; 0.77]
Bernal et al.(BNT162b2)	49	7036	695	58253	0.58	[0.43; 0.78]
Bernal et al.(BNT162b2)*	230	25667	695	58253	0.75	[0.64; 0.87]
Hall et al.(BNT162b2)	71	87278	977	710587	0.59	[0.46; 0.75]
Pouwels et al.(BNT162b2)	714	219626	15834	1986043	0.41	[0.38; 0.44]
Pouwels et al.(ChAdOx1)*	542	379437	15834	1986043	0.18	[0.16; 0.19]
Chemaitelly et al.(BNT162b2)	63	154	3165	6302	0.69	[0.50; 0.95]
Tenforde et al.(BNT162b2/mRNA−1273)	28	354	112	1002	0.68	[0.44; 1.05]
Bruxvoort et al.(mRNA−1273)	14	649	1409	7889	0.10	[0.06; 0.17]
Abu−Raddad et al.(BNT162b2)	773	1888	51973	103604	0.69	[0.63; 0.76]
Eyre et al.(ChAdOx1)	3739	12307	52321	65117	0.11	[0.10; 0.11]
Eyre et al.(BNT162b2)*	3829	20999	52321	65117	0.05	[0.05; 0.06]
		**757528**		**5084011**		
**Random effects model**					**0.34**	**[0.18; 0.64]**

Heterogeneity: $I^2 = 100\%$, $\tau^2 = 1.1959$, $p = 0$

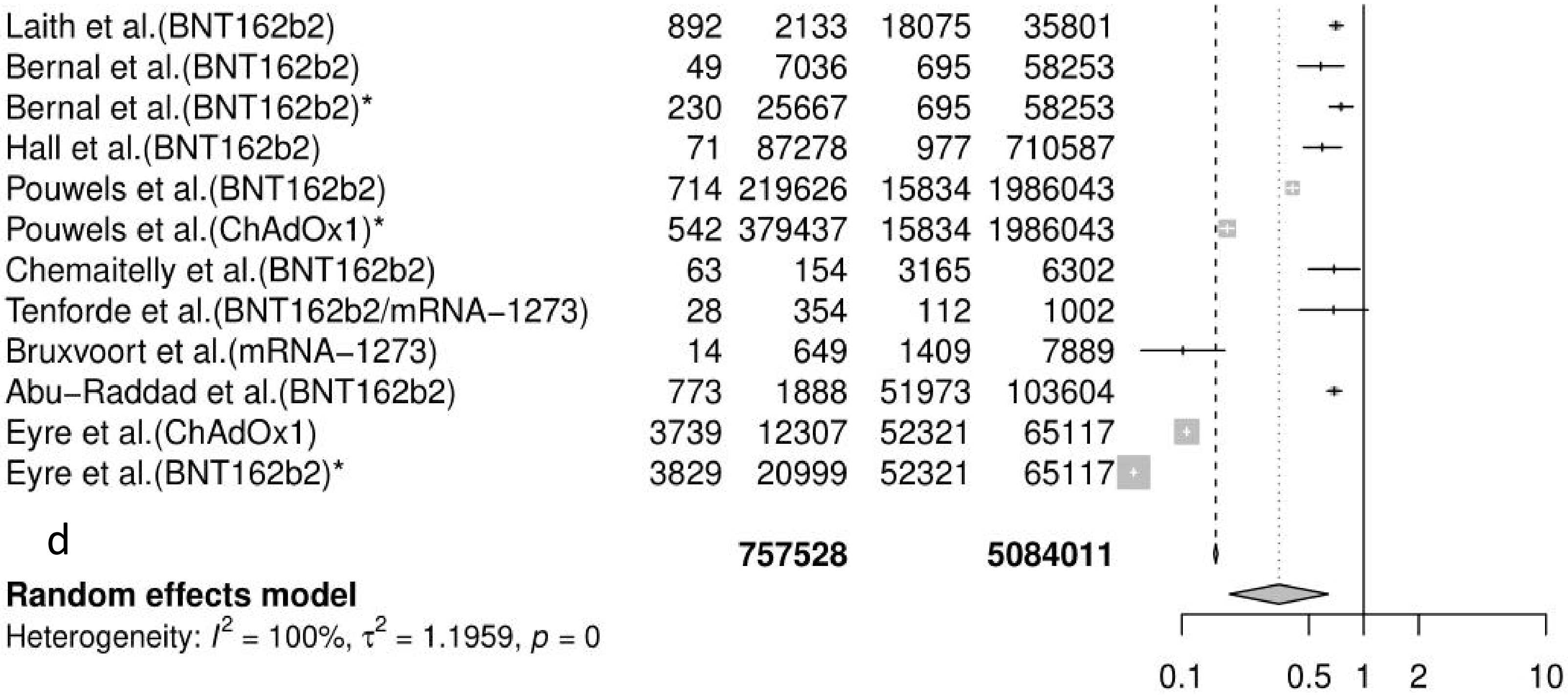

Study	Experimental Events	Experimental Total	Control Events	Control Total	OR	95%-CI
Laith et al.(BNT162b2)	50	515	16354	32293	0.10	[0.08; 0.14]
Bernal et al.(BNT162b2)	13	6412	695	58253	0.17	[0.10; 0.29]
Bernal et al.(ChAdOx1)*	14	2071	695	58253	0.56	[0.33; 0.96]
Hall et al.(BNT162b2)	9	20978	977	710587	0.31	[0.16; 0.60]
Haas et al.(BNT162b2)	3442	4714932	103833	1823979	0.01	[0.01; 0.01]
Emary et al.(ChAdOx1)	21	4244	54	4290	0.39	[0.24; 0.65]
Kustin et al.(BNT162b2)	344	496	355	496	0.90	[0.68; 1.18]
Rovida et al.(BNT162b2)	33	3720	20	346	0.15	[0.08; 0.26]
Pouwels et al.(BNT162b2)	71	109237	15834	1986043	0.08	[0.06; 0.10]
Pouwels et al.(ChAdOx1) *	26	65636	15834	1986043	0.05	[0.03; 0.07]
Heath et al.(NVX−CoV2373)	8	7020	58	7019	0.14	[0.07; 0.29]
Chemaitelly et al.(BNT162b2)	118	532	9512	18728	0.28	[0.22; 0.34]
Tenforde et al.(BNT162b2/mRNA−1273)	9	475	112	1002	0.15	[0.08; 0.31]
Duerr et al.(BNT162b2/mRNA−1273/JNJ−78436735)	26	76	29	76	0.84	[0.43; 1.63]
Bruxvoort et al.(mRNA−1273)	13	1747	1409	6785	0.03	[0.02; 0.05]
Dunkle et al.(NVX−CoV2373)	4	17312	27	8140	0.07	[0.02; 0.20]
Abu−Raddad et al.(BNT162b2)	50	515	16354	32293	0.10	[0.08; 0.14]
Eyre et al.(ChAdOx1)	151	32363	52321	65117	0.00	[0.00; 0.00]
Eyre et al.(BNT162b2)*	337	15457	52321	65117	0.01	[0.00; 0.01]
Mor et al.(BNT162b2)	584	717	2254	2333	0.15	[0.11; 0.21]
e		**5004455**		**6867193**		
**Random effects model**					**0.10**	**[0.05; 0.21]**

Heterogeneity: $I^2 = 100\%$, $\tau^2 = 2.8773$, $p = 0$

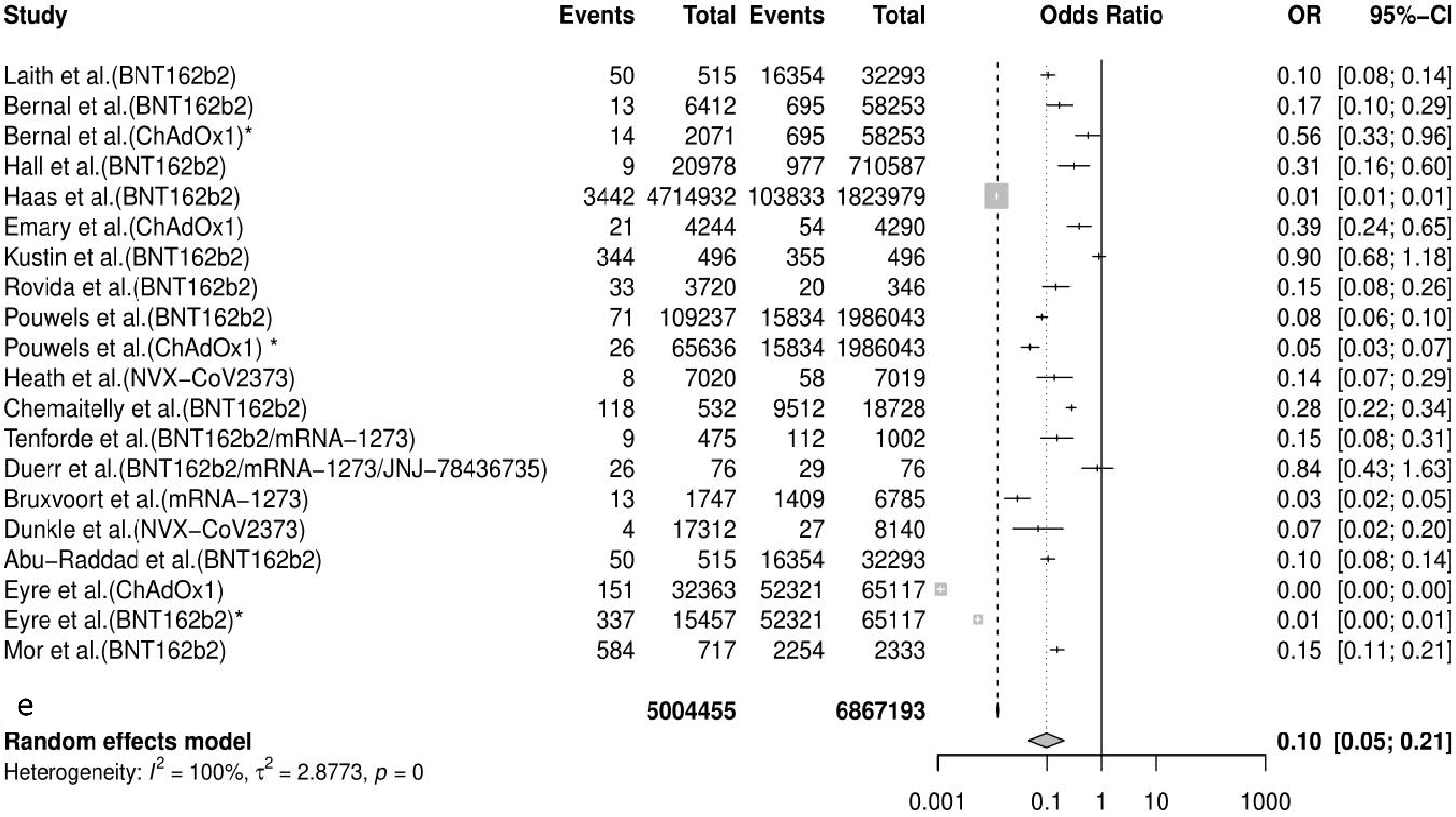

Study	Experimental Events	Experimental Total	Control Events	Control Total	Odds Ratio	OR	95%-CI
Laith et al.(BNT162b2)	892	2133	18075	35801		0.70	[0.65; 0.77]
Bernal et al.(BNT162b2)	49	7036	695	58253		0.58	[0.43; 0.78]
Hall et al.(BNT162b2)	71	87278	977	710587		0.59	[0.46; 0.75]
Pouwels et al.(BNT162b2)	714	219626	15834	1986043		0.41	[0.38; 0.44]
Chemaitelly et al.(BNT162b2)	63	154	3165	6302		0.69	[0.50; 0.95]
Abu−Raddad et al.(BNT162b2)	773	1888	51973	103604		0.69	[0.63; 0.76]
Eyre et al.(BNT162b2)	3829	20999	52321	65117		0.05	[0.05; 0.06]
Bruxvoort et al.(mRNA−1273)	14	649	1409	7889		0.10	[0.06; 0.17]
		**339763**		**2973596**			
**Random effects model**						**0.36**	**[0.13; 1.00]**

f

Heterogeneity: $I^2 = 100\%$, $\tau^2 = 2.1904$, $p = 0$

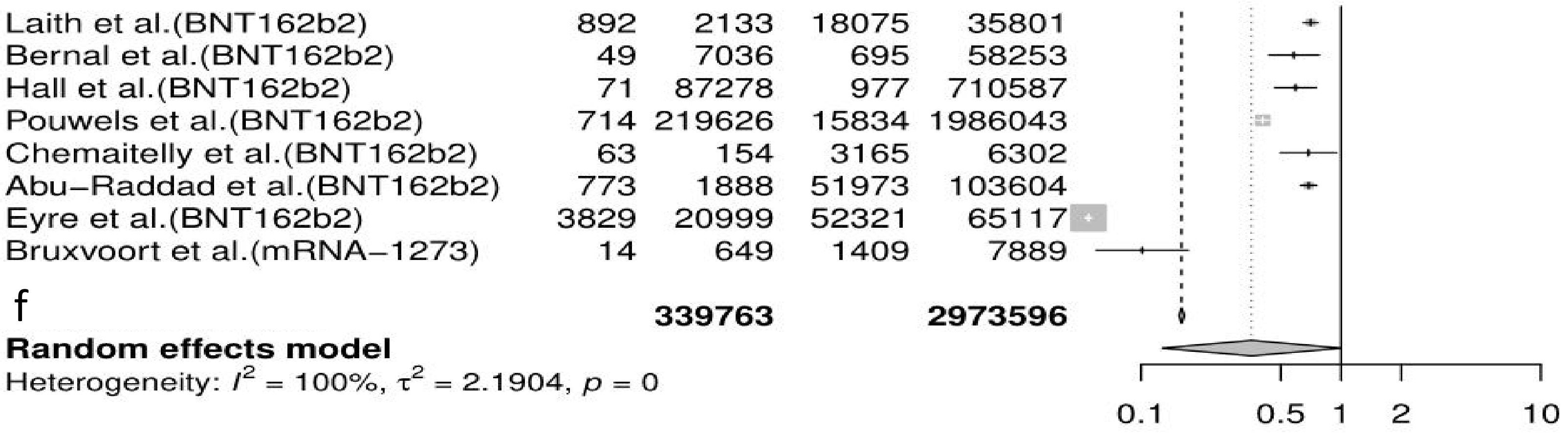

Study	Experimental Events	Experimental Total	Control Events	Control Total	Odds Ratio	OR	95%-CI
Laith et al.(BNT162b2)	50	515	16354	32293		0.10	[0.08; 0.14]
Bernal et al.(BNT162b2)	13	6412	695	58253		0.17	[0.10; 0.29]
Hall et al.(BNT162b2)	9	20978	977	710587		0.31	[0.16; 0.60]
Haas et al.(BNT162b2)	3442	4714932	103833	1823979		0.01	[0.01; 0.01]
Kustin et al.(BNT162b2)	344	496	355	496		0.90	[0.68; 1.18]
Rovida et al.(BNT162b2)	33	3720	20	346		0.15	[0.08; 0.26]
Pouwels et al.(BNT162b2)	71	109237	15834	1986043		0.08	[0.06; 0.10]
Chemaitelly et al.(BNT162b2)	118	532	9512	18728		0.28	[0.22; 0.34]
Abu−Raddad et al.(BNT162b2)	50	515	16354	32293		0.10	[0.08; 0.14]
Eyre et al.(BNT162b2)	337	15457	52321	65117		0.01	[0.00; 0.01]
Mor et al.(BNT162b2)	584	717	2254	2333		0.15	[0.11; 0.21]
Duerr et al.(BNT162b2/mRNA−1273/JNJ−78436735)	26	76	29	76		0.84	[0.43; 1.63]
Bruxvoort et al.(mRNA−1273)	13	1747	1409	6785		0.03	[0.02; 0.05]
		**4875334**		**4737329**			
**Random effects model**						**0.11**	**[0.05; 0.26]**

g

Heterogeneity: $I^2 = 100\%$, $\tau^2 = 2.5342$, $p = 0$

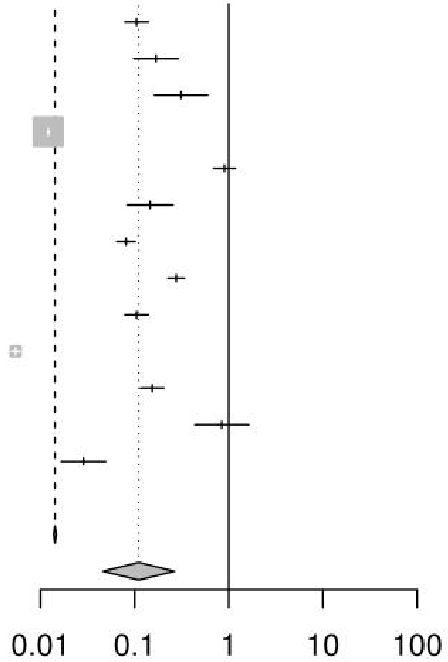

Study	Experimental Events	Experimental Total	Control Events	Control Total	OR	95%-CI
Heath et al.(NVX–CoV2373)	8	7020	58	7019	0.14	[0.07; 0.29]
Dunkle et al.(NVX–CoV2373)	4	17312	27	8140	0.07	[0.02; 0.20]
**Fixed effect model**		**24332**		**15159**	**0.11**	**[0.06; 0.20]**

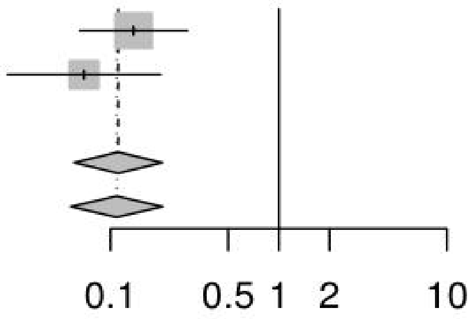

h

Heterogeneity: $I^2 = 7\%$, $\tau^2 = 0.0160$, $p = 0.30$

Study	Experimental Events	Experimental Total	Control Events	Control Total	OR	95%-CI
Madhi etal.(ChAdOx1)	14	2071	695	58253	0.56	[0.33; 0.96]
Irfan et al.(ChAdOx1)	26	65636	15834	1986043	0.05	[0.03; 0.07]
Emary et al.(ChAdOx1)	21	4244	54	4290	0.39	[0.24; 0.65]
Eyre et al.(ChAdOx1)	151	32363	52321	65117	0.00	[0.00; 0.00]
		**104314**		**2113703**		
**Random effects model**					**0.06**	**[0.00; 1.70]**

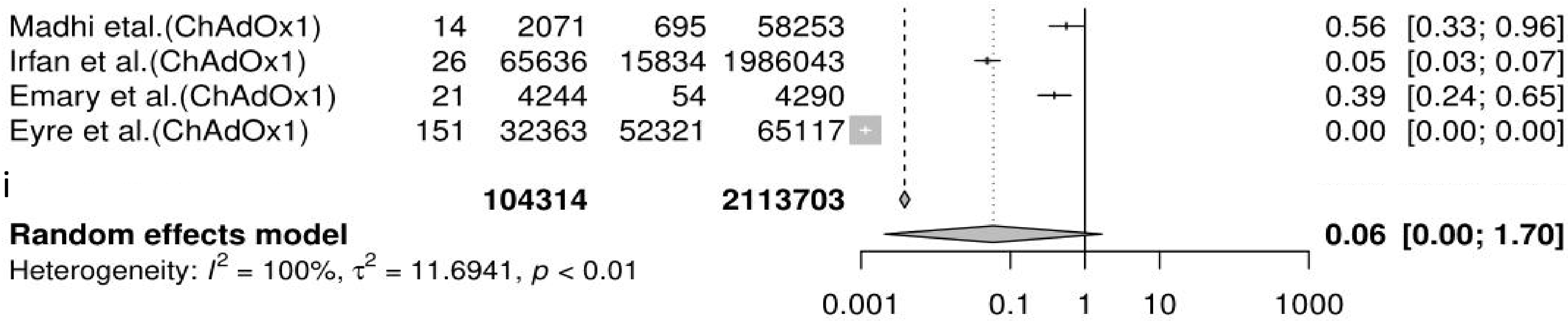

i

Heterogeneity: $I^2 = 100\%$, $\tau^2 = 11.6941$, $p < 0.01$

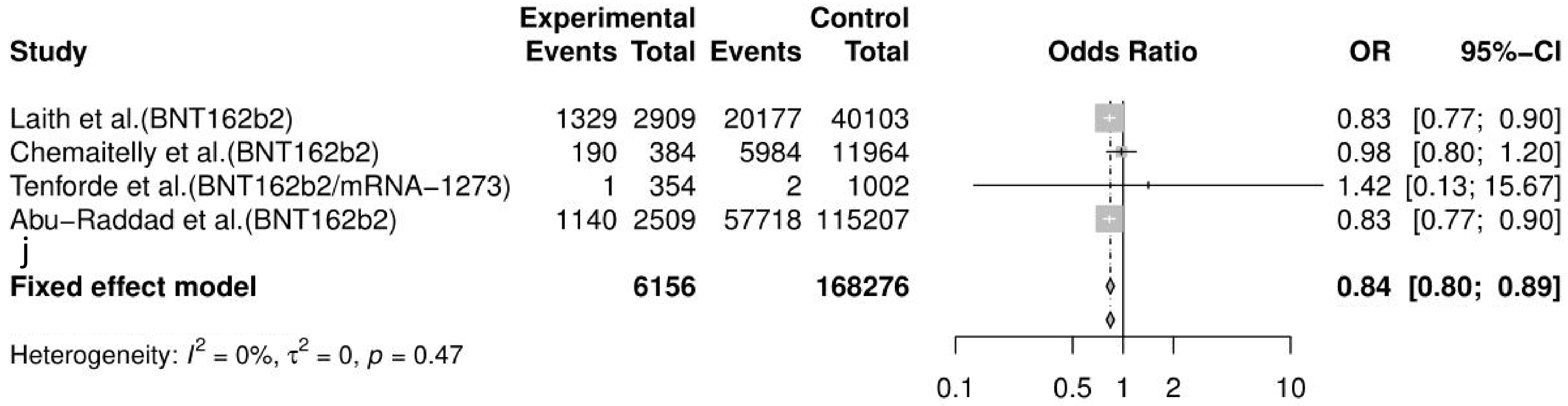
Experimental
Control
Study
Events Total Events Total
Odds Ratio
OR 95%-CI
Laith et al.(BNT162b2) 1329 2909 20177 40103 0.83 [0.77; 0.90]
Chemaitelly et al.(BNT162b2) 190 384 5984 11964 0.98 [0.80; 1.20]
Tenforde et al.(BNT162b2/mRNA-1273) 1 354 2 1002 1.42 [0.13; 15.67]
Abu-Raddad et al.(BNT162b2) 1140 2509 57718 115207 0.83 [0.77; 0.90]
j
Fixed effect model 6156 168276 0.84 [0.80; 0.89]
Heterogeneity: I^2 = 0%, τ^2 = 0, p = 0.47
0.1 0.5 1 2 10

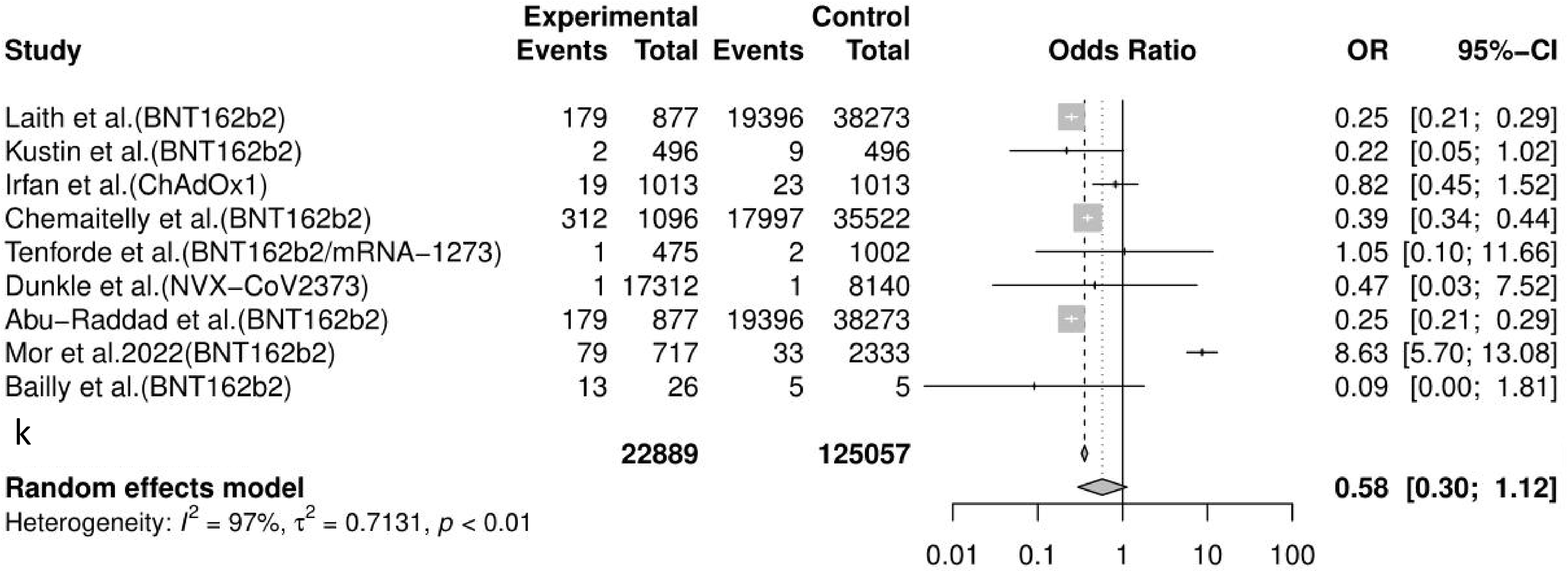
Experimental
Control
Study
Events Total Events Total
Odds Ratio
OR 95%-CI
Laith et al.(BNT162b2) 179 877 19396 38273 0.25 [0.21; 0.29]
Kustin et al.(BNT162b2) 2 496 9 496 0.22 [0.05; 1.02]
Irfan et al.(ChAdOx1) 19 1013 23 1013 0.82 [0.45; 1.52]
Chemaitelly et al.(BNT162b2) 312 1096 17997 35522 0.39 [0.34; 0.44]
Tenforde et al.(BNT162b2/mRNA-1273) 1 475 2 1002 1.05 [0.10; 11.66]
Dunkle et al.(NVX-CoV2373) 1 17312 1 8140 0.47 [0.03; 7.52]
Abu-Raddad et al.(BNT162b2) 179 877 19396 38273 0.25 [0.21; 0.29]
Mor et al.2022(BNT162b2) 79 717 33 2333 8.63 [5.70; 13.08]
Bailly et al.(BNT162b2) 13 26 5 5 0.09 [0.00; 1.81]
k
22889 125057
Random effects model 0.58 [0.30; 1.12]
Heterogeneity: I^2 = 97%, τ^2 = 0.7131, p < 0.01
0.01 0.1 1 10 100

Study	Experimental Events	Experimental Total	Control Events	Control Total	OR	95%-CI
Laith et al.(BNT162b2)	1329	2909	20177	40103	0.83	[0.77; 0.90]
Chemaitelly et al.(BNT162b2)	190	384	5984	11964	0.98	[0.80; 1.20]
Abu−Raddad et al.(BNT162b2)	1140	2509	57718	115207	0.83	[0.77; 0.90]
Tenforde et al.(BNT162b2/mRNA−1273)	1	354	2	1002	1.42	[0.13; 15.67]
l						
**Fixed effect model**		**6156**		**168276**	**0.84**	**[0.80; 0.89]**

Heterogeneity: $I^2 = 0\%$, $\tau^2 = 0$, $p = 0.47$

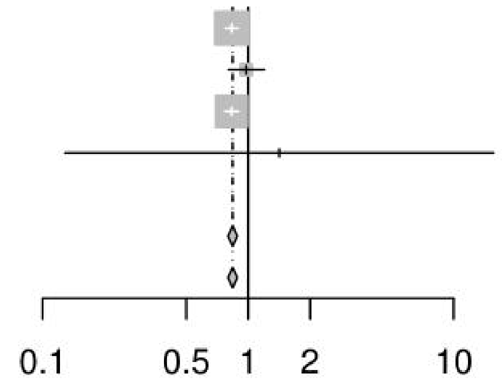

Study	Experimental Events	Experimental Total	Control Events	Control Total	OR	95%-CI
Laith et al.(BNT162b2)	179	877	19396	38273	0.25	[0.21; 0.29]
Kustin et al.(BNT162b2)	2	496	9	496	0.22	[0.05; 1.02]
Chemaitelly et al.(BNT162b2)	312	1096	17997	35522	0.39	[0.34; 0.44]
Abu−Raddad et al.(BNT162b2)	179	877	19396	38273	0.25	[0.21; 0.29]
Mor et al.2022.(BNT162b2)	79	717	33	2333	8.63	[5.70; 13.08]
Tenforde et al.(BNT162b2/mRNA−1273)	1	475	2	1002	1.05	[0.10; 11.66]
m		**4538**		**115899**		
**Random effects model**					**0.60**	**[0.28; 1.28]**

Heterogeneity: $I^2 = 98\%$, $\tau^2 = 0.7275$, $p < 0.01$

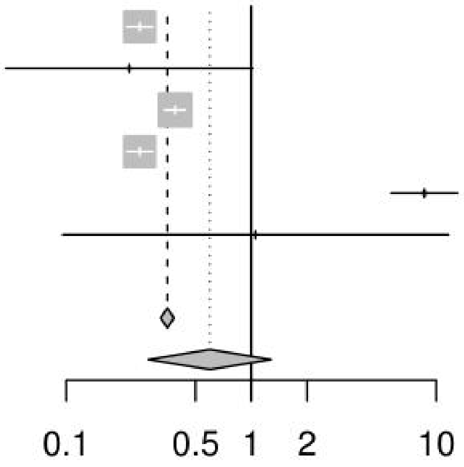

n

Study	Experimental Events	Experimental Total	Control Events	Control Total	OR	95%-CI
Tenforde et al.(BNT162b2/mRNA−1273)	2	354	10	1002	0.56	[0.12; 2.58]
Ranzani et al.(CoronaVac)	7040	15932	21816	38385	0.60	[0.58; 0.62]
Vignier et al.(BNT162b2)	6	12	3	7	1.33	[0.20; 8.71]
Hitchings et al.(ChAdOx1)	20897	37713	90797	159950	0.95	[0.93; 0.97]
Bruxvoort et al.(mRNA−1273)	8	140	340	1948	0.29	[0.14; 0.59]
		**54151**		**201292**		
**Random effects model**					**0.65**	**[0.44; 0.95]**

Heterogeneity: $I^2 = 99\%$, $\tau^2 = 0.1037$, $p < 0.01$

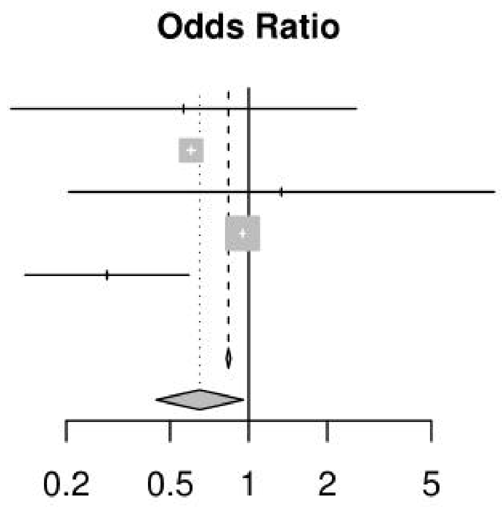

o

Study	Experimental Events	Experimental Total	Control Events	Control Total	OR	95%-CI
Clemens et al.(ChAdOx1)	16	4772	51	4661	0.30	[0.17; 0.53]
Ranzani et al.(CoronaVac)	3620	9046	21816	38385	0.51	[0.48; 0.53]
Vignier et al.(BNT162b2)	15	25	3	7	2.00	[0.37; 10.92]
Tenforde et al.(BNT162b2/mRNA−1273)	2	475	10	1002	0.42	[0.09; 1.92]
Duerr et al.(BNT162b2/mRNA−1273/JNJ−78436735)	1	76	3	76	0.32	[0.03; 3.19]
Hitchings et al.(ChAdOx1)	983	2370	90797	159950	0.54	[0.50; 0.59]
Dunkle et al.(NVX−CoV2373)	0	17312	2	8140	0.09	[0.00; 1.96]
Bruxvoort et al.(mRNA−1273)	9	561	340	1533	0.06	[0.03; 0.11]
		**34637**		**213754**		
**Random effects model**					**0.39**	**[0.30; 0.50]**

Heterogeneity: $I^2 = 86\%$, $\tau^2 = 0.0486$, $p < 0.01$

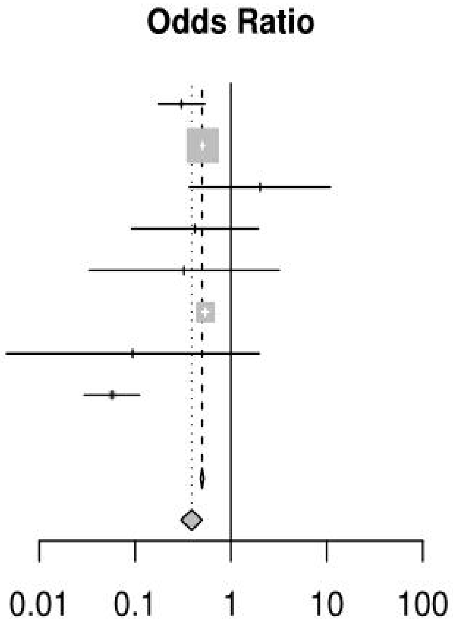

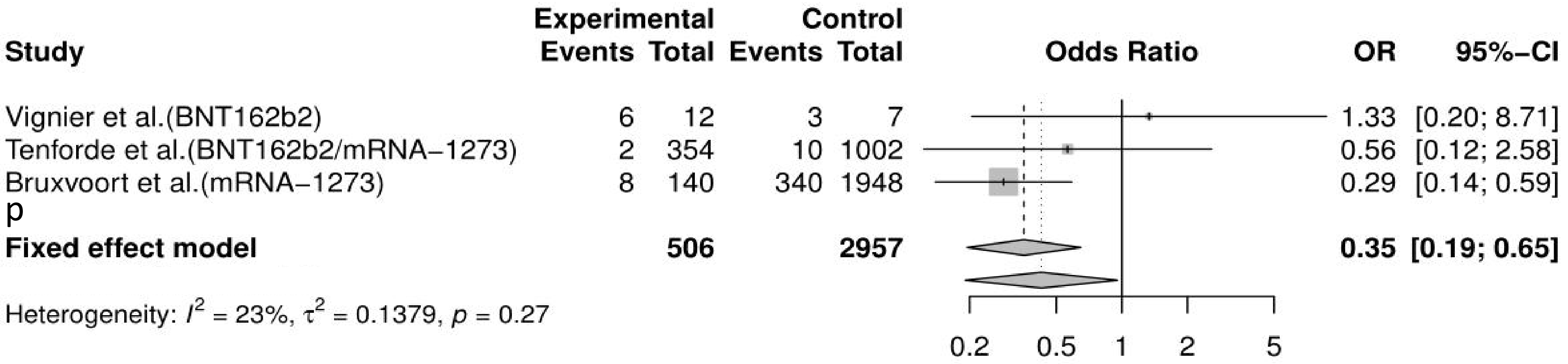
Study
Experimental Events Total
Control Events Total
Odds Ratio
OR 95%-CI
Vignier et al.(BNT162b2) 6 12 3 7 1.33 [0.20; 8.71]
Tenforde et al.(BNT162b2/mRNA-1273) 2 354 10 1002 0.56 [0.12; 2.58]
Bruxvoort et al.(mRNA-1273) 8 140 340 1948 0.29 [0.14; 0.59]
p
Fixed effect model 506 2957 0.35 [0.19; 0.65]
Heterogeneity: I^2 = 23%, τ^2 = 0.1379, p = 0.27
0.2
0.5
1
2
5

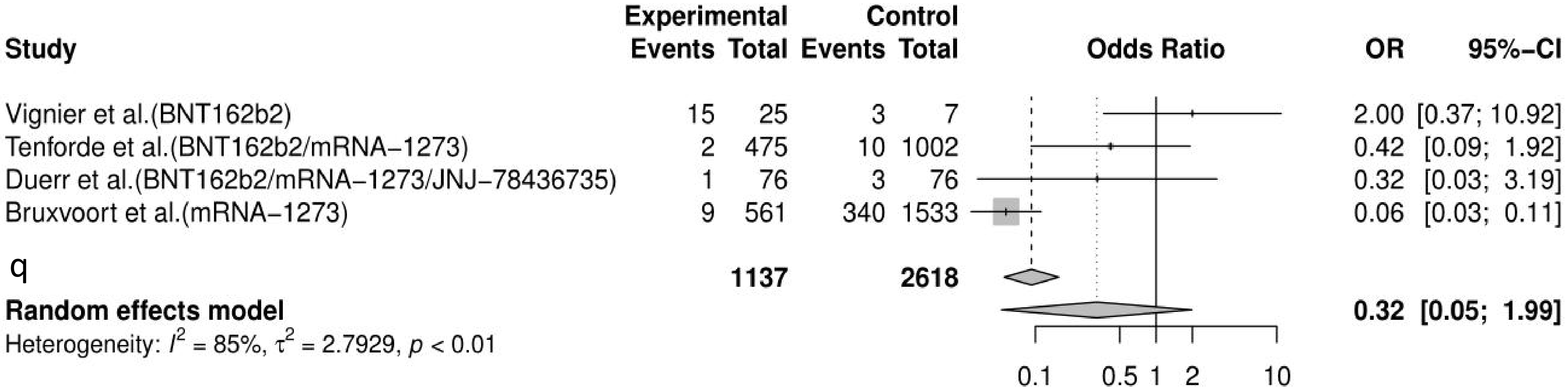
Study
Experimental Events Total
Control Events Total
Odds Ratio
OR 95%-CI
Vignier et al.(BNT162b2) 15 25 3 7 2.00 [0.37; 10.92]
Tenforde et al.(BNT162b2/mRNA-1273) 2 475 10 1002 0.42 [0.09; 1.92]
Duerr et al.(BNT162b2/mRNA-1273/JNJ-78436735) 1 76 3 76 0.32 [0.03; 3.19]
Bruxvoort et al.(mRNA-1273) 9 561 340 1533 0.06 [0.03; 0.11]
q
1137 2618
Random effects model 0.32 [0.05; 1.99]
Heterogeneity: I^2 = 85%, τ^2 = 2.7929, $p < 0.01$
0.1
0.5
1
2
10

Study	Experimental Events	Experimental Total	Control Events	Control Total	OR	95%-CI
Clemens et al.(ChAdOx1)	16	4772	51	4661	0.30	[0.17; 0.53]
Hitchings et al.(ChAdOx1)	983	2370	90797	159950	0.54	[0.50; 0.59]
r		**7142**		**164611**		
**Random effects model**					**0.43**	**[0.25; 0.75]**

Heterogeneity: $I^2 = 74\%$, $\tau^2 = 0.1226$, $p = 0.05$

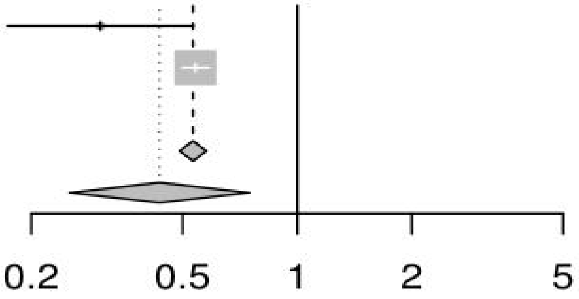

Study	Experimental Events	Experimental Total	Control Events	Control Total	OR	95%-CI
Bernal et al.(BNT162b2)	344	7036	4891	58253	0.56	[0.50; 0.63]
Bernal et al.(ChAdOx1)*	1137	25667	4891	58253	0.51	[0.47; 0.54]
Grant et al.(BNT162b2)	1160	2149	4274	6673	0.66	[0.60; 0.73]
Tang et al. (BNT162b2)	39	293	2744	13691	0.61	[0.44; 0.86]
Tang et al. (mRNA−1273)*	21	280	2760	13788	0.32	[0.21; 0.51]
Tang et al. (BNT162b2/mRNA−1273)**	61	569	2873	17212	0.60	[0.46; 0.78]
Pouwels et al.(BNT162b2)	422	42503	611	77810	1.27	[1.12; 1.44]
Pouwels et al.(ChAdOx1)*	214	70609	611	77810	0.38	[0.33; 0.45]
Pouwels et al.(mRNA−1273)**	32	6244	611	77810	0.65	[0.46; 0.93]
Chemaitelly et al.(BNT162b2)	54	167	4266	8473	0.47	[0.34; 0.65]
Li et al.(CoronaVac)	25	113	37	142	0.81	[0.45; 1.44]
Thiruvengadam et al.(ChAdOx1)	157	2451	181	1994	0.69	[0.55; 0.86]
Bruxvoort et al.(mRNA−1273)	15	351	1795	9969	0.20	[0.12; 0.34]
Reis et al.(BNT162b2)	863	126405	329	126967	2.65	[2.33; 3.01]
s		**284837**		**548845**		
**Random effects model**					**0.62**	**[0.45; 0.85]**

Heterogeneity: $I^2 = 98\%$, $\tau^2 = 0.3453$, $p < 0.01$

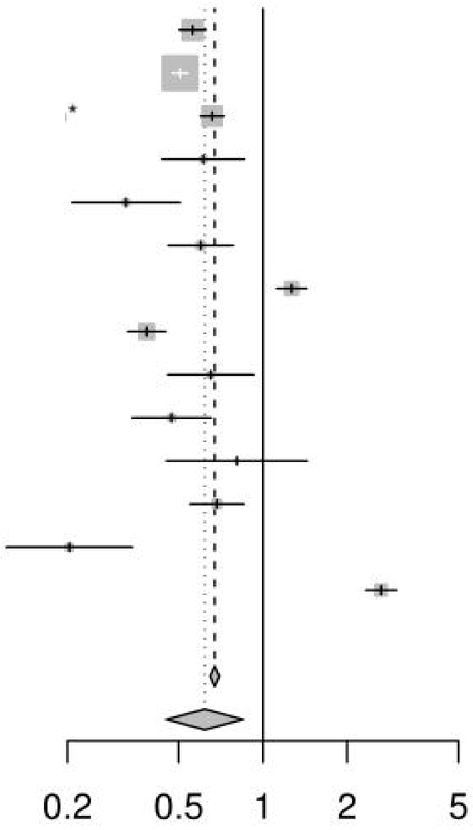

Study	Experimental Events	Experimental Total	Control Events	Control Total	OR	95%-CI
Bernal et al.(BNT162b2)	28	6412	4891	58253	0.05	[0.03; 0.07]
Bernal et al.(ChAdOx1)*	46	2071	4891	58253	0.25	[0.18; 0.33]
Grant et al.(BNT162b2)	2202	3927	4274	6673	0.72	[0.66; 0.78]
Grant et al.(ChAdOx1/BNT162b2)*	153	247	4274	6673	0.91	[0.70; 1.19]
Tang et al. (BNT162b2)	998	6590	2848	13233	0.65	[0.60; 0.70]
Tang et al. (mRNA–1273)*	150	1718	2797	13380	0.36	[0.30; 0.43]
Tang et al. (BNT162b2/mRNA–1273)**	1174	8036	2976	13604	0.61	[0.57; 0.66]
Pouwels et al.(BNT162b2)	488	227907	611	77810	0.27	[0.24; 0.31]
Pouwels et al.(ChAdOx1)*	1328	379985	611	77810	0.44	[0.40; 0.49]
Chemaitelly et al.(BNT162b2)	650	1768	12876	25284	0.56	[0.51; 0.62]
Li et al.(CoronaVac)	12	109	37	142	0.35	[0.17; 0.71]
Williams et al.(ChAdOx1–S)	18	30	3	6	1.50	[0.26; 8.71]
Desai et al.(BBV152)	186	449	535	934	0.53	[0.42; 0.66]
Bruxvoort et al.(mRNA–1273)	232	4820	1795	7342	0.16	[0.14; 0.18]
Reis et al.(BNT162b2)	75	46815	818	46384	0.09	[0.07; 0.11]
Olson et al.(BNT162b2)	6	179	93	285	0.07	[0.03; 0.17]
t		**691063**		**406066**		
**Random effects model**					**0.32**	**[0.24; 0.43]**

Heterogeneity: $I^2 = 98\%$, $\tau^2 = 0.3147$, $p < 0.01$

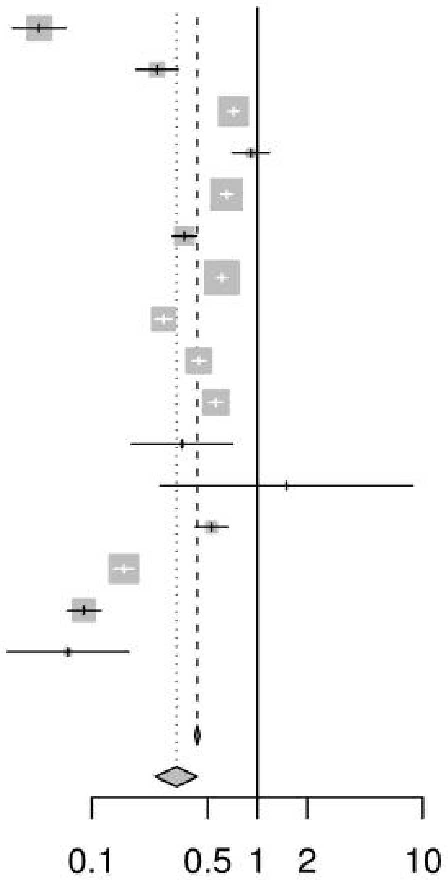

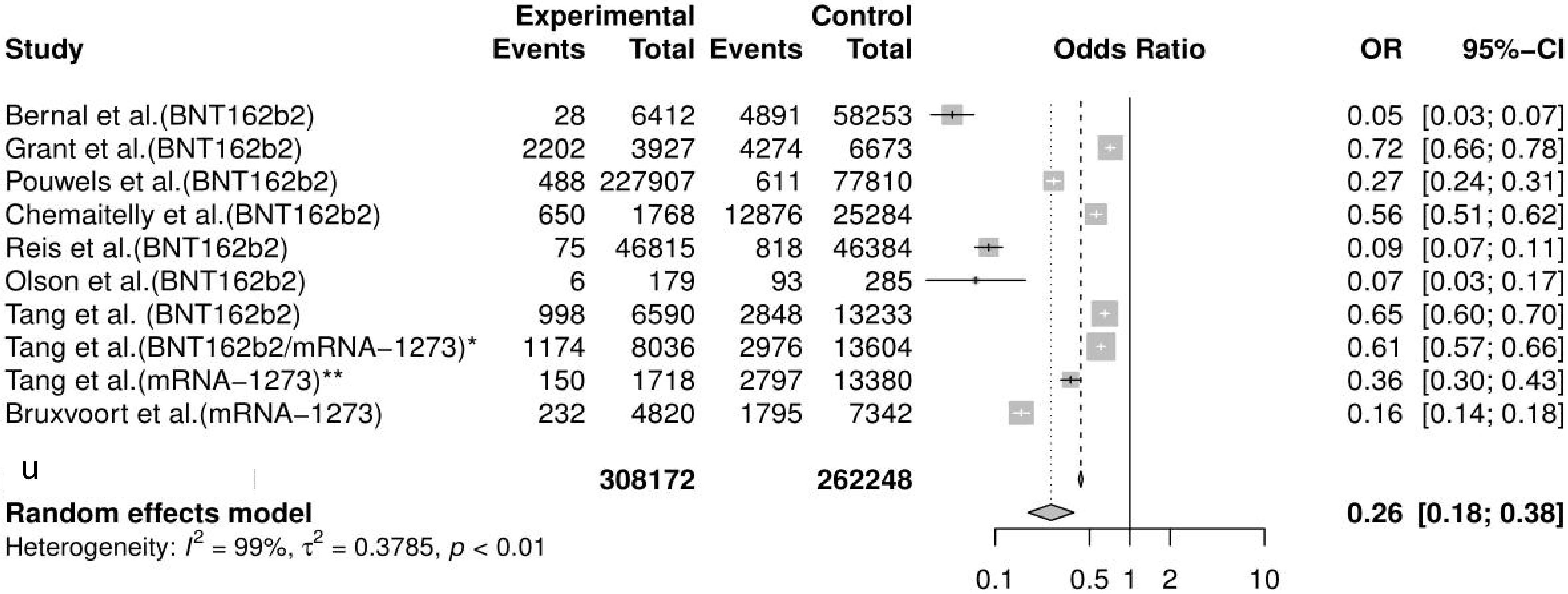

Study
Experimental Events Total
Control Events Total
Odds Ratio
OR 95%−CI
Bernal et al.(BNT162b2) 28 6412 4891 58253 0.05 [0.03; 0.07]
Grant et al.(BNT162b2) 2202 3927 4274 6673 0.72 [0.66; 0.78]
Pouwels et al.(BNT162b2) 488 227907 611 77810 0.27 [0.24; 0.31]
Chemaitelly et al.(BNT162b2) 650 1768 12876 25284 0.56 [0.51; 0.62]
Reis et al.(BNT162b2) 75 46815 818 46384 0.09 [0.07; 0.11]
Olson et al.(BNT162b2) 6 179 93 285 0.07 [0.03; 0.17]
Tang et al. (BNT162b2) 998 6590 2848 13233 0.65 [0.60; 0.70]
Tang et al.(BNT162b2/mRNA−1273)* 1174 8036 2976 13604 0.61 [0.57; 0.66]
Tang et al.(mRNA−1273)** 150 1718 2797 13380 0.36 [0.30; 0.43]
Bruxvoort et al.(mRNA−1273) 232 4820 1795 7342 0.16 [0.14; 0.18]
u
308172 262248
Random effects model 0.26 [0.18; 0.38]
Heterogeneity: I^2 = 99%, τ^2 = 0.3785, p < 0.01
0.1 0.5 1 2 10

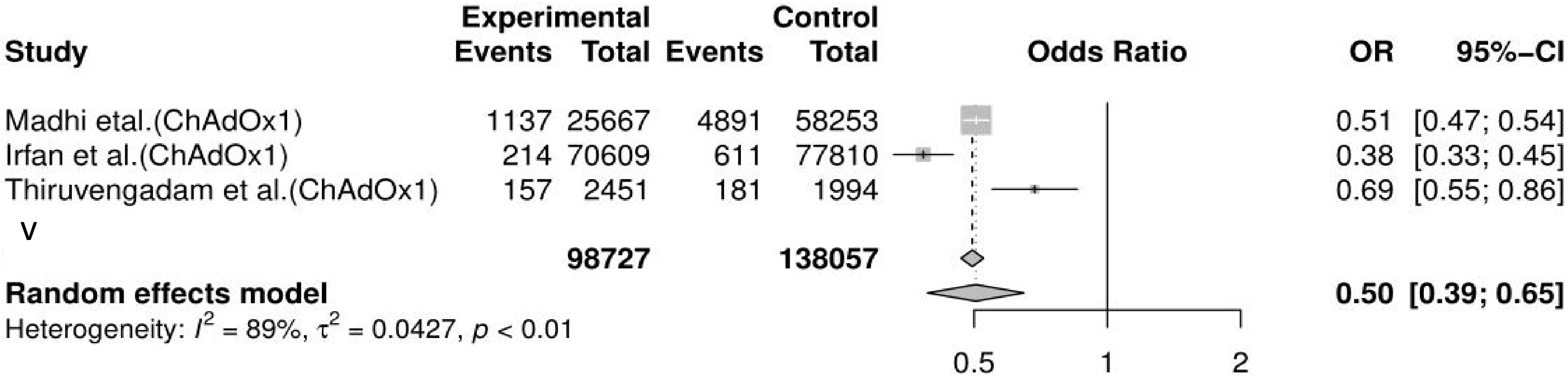

Study
Experimental Events Total
Control Events Total
Odds Ratio
OR 95%−CI
Madhi etal.(ChAdOx1) 1137 25667 4891 58253 0.51 [0.47; 0.54]
Irfan et al.(ChAdOx1) 214 70609 611 77810 0.38 [0.33; 0.45]
Thiruvengadam et al.(ChAdOx1) 157 2451 181 1994 0.69 [0.55; 0.86]
v
98727 138057
Random effects model 0.50 [0.39; 0.65]
Heterogeneity: I^2 = 89%, τ^2 = 0.0427, p < 0.01
0.5 1 2

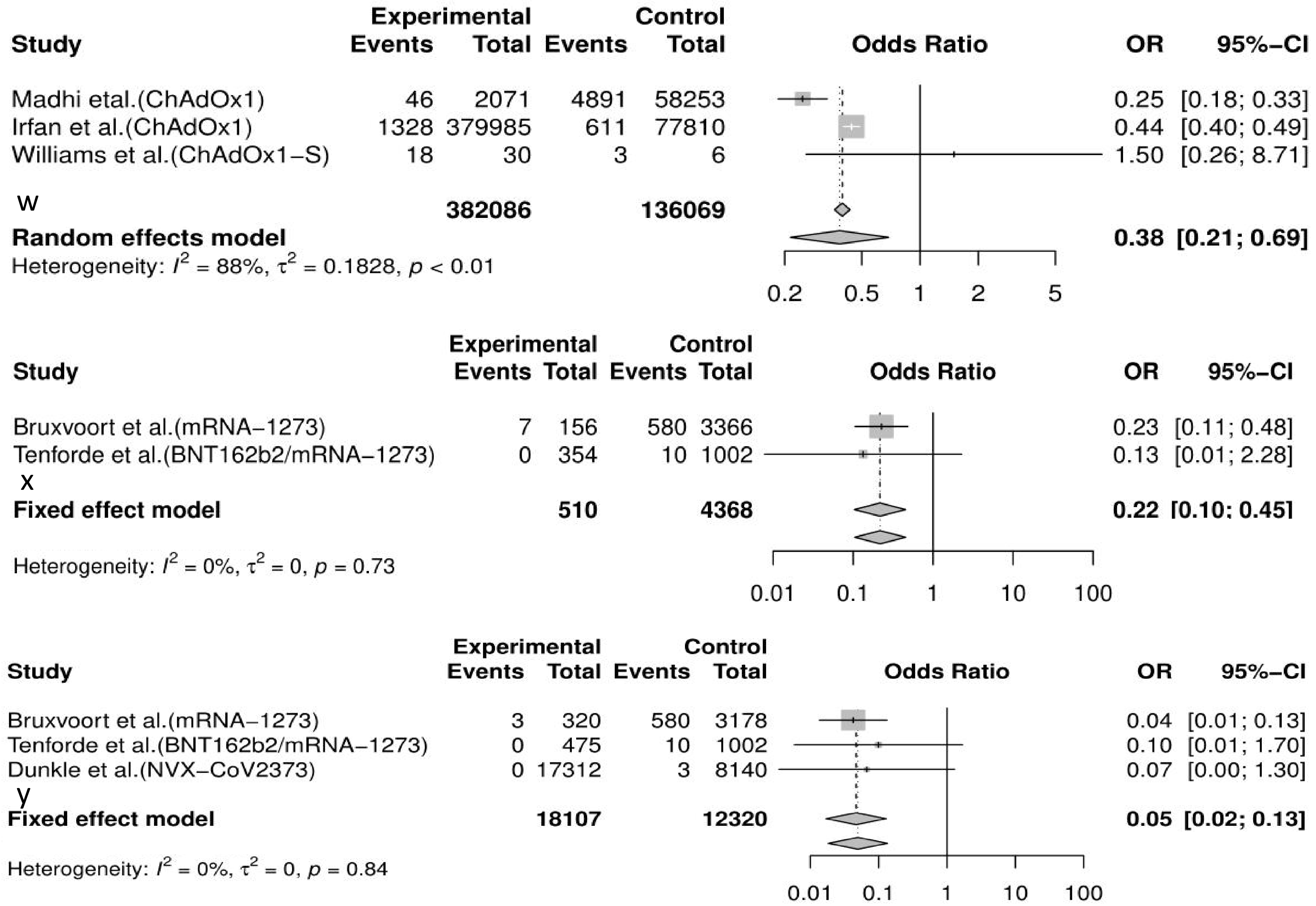
Study
Experimental Events
Experimental Total
Control Events
Control Total
Odds Ratio
OR
95%−CI
Madhi etal.(ChAdOx1) 46 2071 4891 58253 0.25 [0.18; 0.33]
Irfan et al.(ChAdOx1) 1328 379985 611 77810 0.44 [0.40; 0.49]
Williams et al.(ChAdOx1−S) 18 30 3 6 1.50 [0.26; 8.71]
w
382086 136069
Random effects model 0.38 [0.21; 0.69]
Heterogeneity: I^2 = 88%, τ^2 = 0.1828, p < 0.01
0.2 0.5 1 2 5
Study
Experimental Events
Experimental Total
Control Events
Control Total
Odds Ratio
OR
95%−CI
Bruxvoort et al.(mRNA−1273) 7 156 580 3366 0.23 [0.11; 0.48]
Tenforde et al.(BNT162b2/mRNA−1273) 0 354 10 1002 0.13 [0.01; 2.28]
x
Fixed effect model 510 4368 0.22 [0.10; 0.45]
Heterogeneity: I^2 = 0%, τ^2 = 0, p = 0.73
0.01 0.1 1 10 100
Study
Experimental Events
Experimental Total
Control Events
Control Total
Odds Ratio
OR
95%−CI
Bruxvoort et al.(mRNA−1273) 3 320 580 3178 0.04 [0.01; 0.13]
Tenforde et al.(BNT162b2/mRNA−1273) 0 475 10 1002 0.10 [0.01; 1.70]
Dunkle et al.(NVX−CoV2373) 0 17312 3 8140 0.07 [0.00; 1.30]
y
Fixed effect model 18107 12320 0.05 [0.02; 0.13]
Heterogeneity: I^2 = 0%, τ^2 = 0, p = 0.84
0.01 0.1 1 10 100

Study	Experimental Events	Experimental Total	Control Events	Control Total	OR	95%-CI
Tenforde et al.(BNT162b2/mRNA–1273)	0	354	10	1002	0.13	[0.01; 2.28]
Bruxvoort et al.(mRNA–1273)	7	156	580	3366	0.23	[0.11; 0.48]
**Fixed effect model**		**510**		**4368**	**0.22**	**[0.10; 0.45]**

z

Heterogeneity: $I^2 = 0\%$, $\tau^2 = 0$, $p = 0.73$

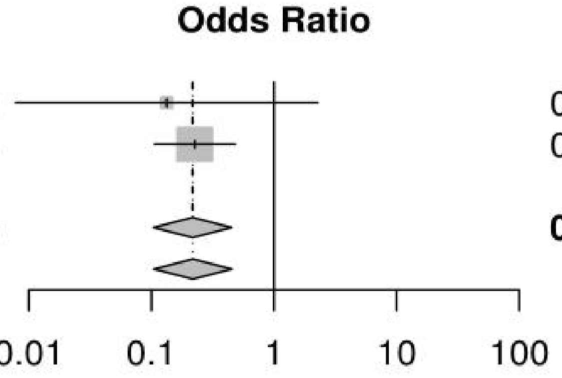

Study	Experimental Events	Experimental Total	Control Events	Control Total	OR	95%-CI
Tenforde et al.(BNT162b2/mRNA–1273)	0	475	10	1002	0.10	[0.01; 1.70]
Bruxvoort et al.(mRNA–1273)	3	320	580	3178	0.04	[0.01; 0.13]
**Fixed effect model**		**795**		**4180**	**0.05**	**[0.02; 0.13]**

aa

Heterogeneity: $I^2 = 0\%$, $\tau^2 = 0$, $p = 0.58$

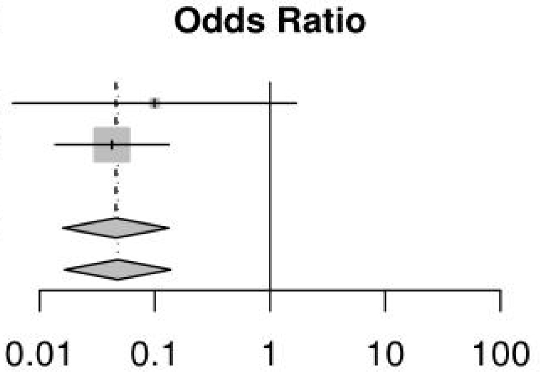

bb

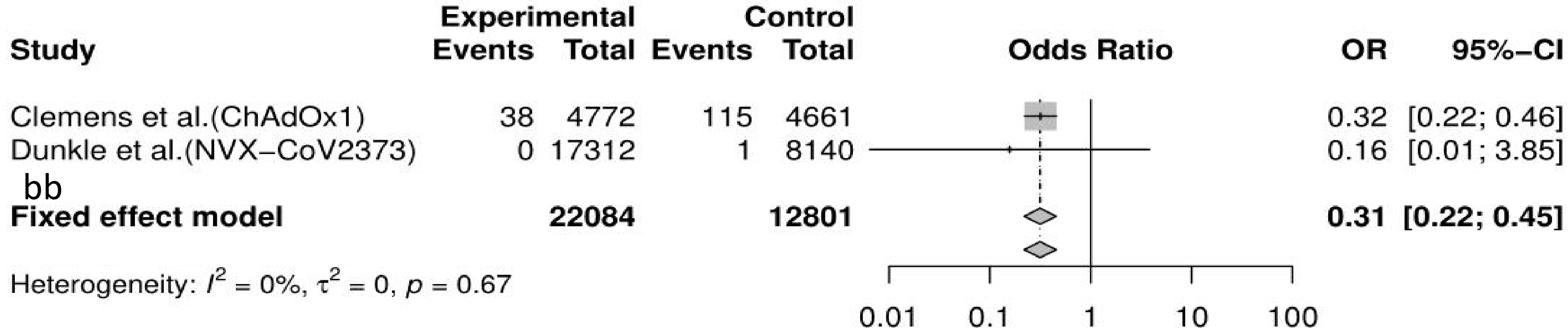
Study
Experimental Events Total
Control Events Total
Odds Ratio
OR 95%-CI
Clemens et al.(ChAdOx1) 38 4772 115 4661 0.32 [0.22; 0.46]
Dunkle et al.(NVX-CoV2373) 0 17312 1 8140 0.16 [0.01; 3.85]
Fixed effect model 22084 12801 0.31 [0.22; 0.45]
Heterogeneity: $I^2 = 0\%$, $\tau^2 = 0$, $p = 0.67$
0.01 0.1 1 10 100

cc

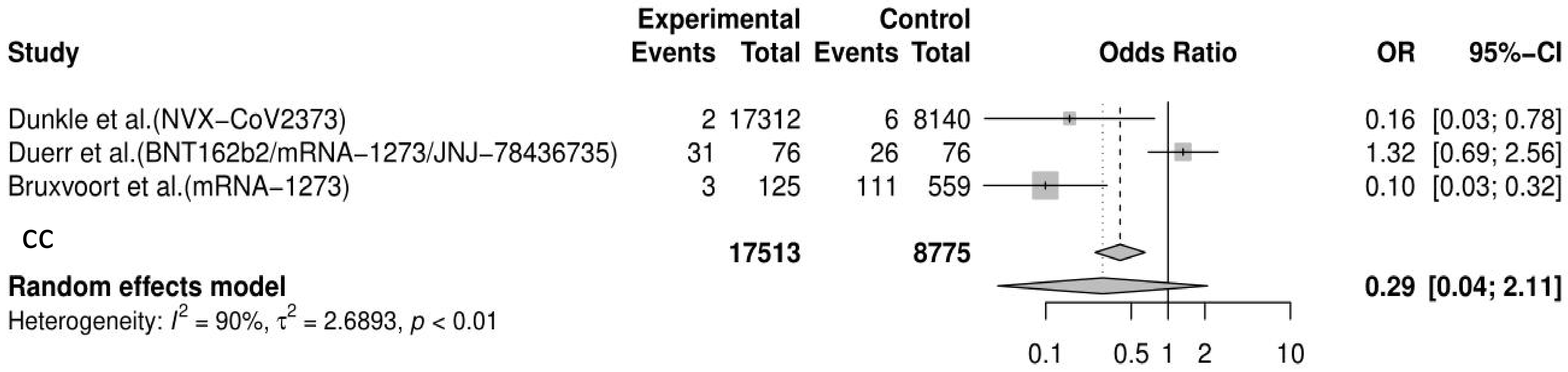
Study
Experimental Events Total
Control Events Total
Odds Ratio
OR 95%-CI
Dunkle et al.(NVX-CoV2373) 2 17312 6 8140 0.16 [0.03; 0.78]
Duerr et al.(BNT162b2/mRNA-1273/JNJ-78436735) 31 76 26 76 1.32 [0.69; 2.56]
Bruxvoort et al.(mRNA-1273) 3 125 111 559 0.10 [0.03; 0.32]
17513 8775
Random effects model 0.29 [0.04; 2.11]
Heterogeneity: $I^2 = 90\%$, $\tau^2 = 2.6893$, $p < 0.01$
0.1 0.5 1 2 10

dd

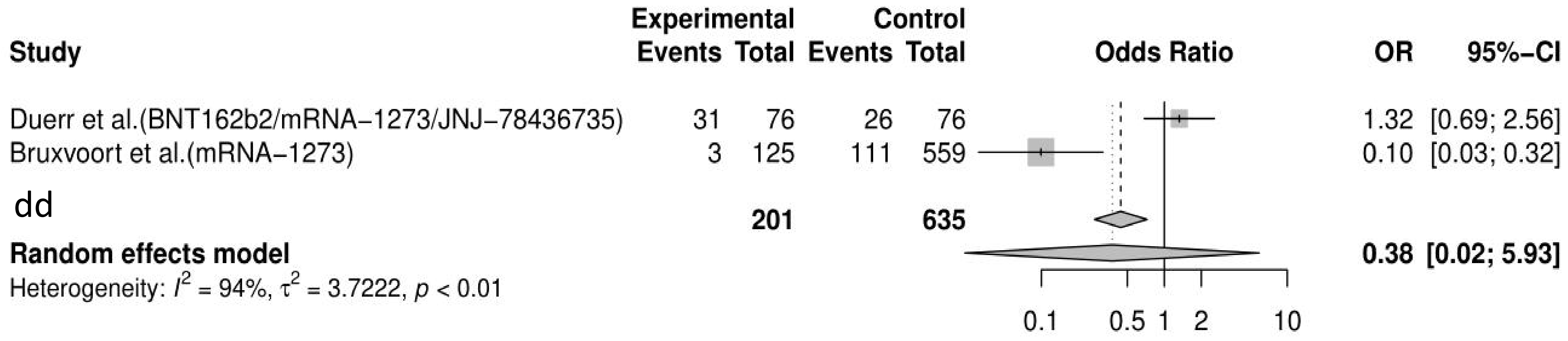
Study
Experimental Events Total
Control Events Total
Odds Ratio
OR 95%-CI
Duerr et al.(BNT162b2/mRNA-1273/JNJ-78436735) 31 76 26 76 1.32 [0.69; 2.56]
Bruxvoort et al.(mRNA-1273) 3 125 111 559 0.10 [0.03; 0.32]
201 635
Random effects model 0.38 [0.02; 5.93]
Heterogeneity: $I^2 = 94\%$, $\tau^2 = 3.7222$, $p < 0.01$
0.1 0.5 1 2 10
