# Supplementary material for: Real-Word Effectiveness of Global COVID-19 Vaccines Against SARS-CoV-2 Variants: A Systematic Review and Meta-Analysis
Source: Front Med (Lausanne). 2022 May 19;9:820544. doi: 10.3389/fmed.2022.820544 (PMC9160927; doi:10.3389/fmed.2022.820544)
Supplement: Supplementary Table 1 — Characteristics of the studies included. [file Table_1.pdf]

Supplementary table 1: Characteristics of the Studies included

Study and Year	Sample size	study design	Participating Countries	Age (Mean/Median and Range/SD)	Male (%)	Vaccine Name (dose)	Vaccine Type	Vaccine Developer	Comparator	Characteristics of Vaccine Recipients	Number of Scheduled Doses (Time of Inoculations)	Study Duration	Variants
Madhi et al. 2021[20]	2026	A multicenter, double-blind, randomized, controlled trial	South Africa	30(18-65)	1145(56.5%)	ChAdOx1(AZD1222)(5× 10^{10} viral particles)	Virus-vectored vaccine	AstraZeneca	Saline placebo	HIV-negative adults	Prime and boost inoculation (0, 21-35 days)	June 24 and November 9, 2020	B.1.351 (501Y.V2)/ D614G(B1)
Shinde et al. 2021[21]	6324	A randomized, observer-blinded, placebo-controlled, phase 2a–b trial	South Africa	32.0（13.0）	2518 (57.4%)	NVX-CoV2373(5 μg of recom Binant spike protein)	Recombinant subunit vaccine	Novavax	Saline placebo	HIV–negative or stable HIV-positive adults	Prime and boost inoculation (0, 21 days)	August 17, 2020, through November 25, 2020,	B.1.351 (501Y.V2)/ D614G(B1)

Hitchings et al. 2021[22]	1056	A matched test-negative case-control study	Brazil	43.1 (NA)	271(25.7%)	CoronaVac (NA)	Inactivated vaccine	Sinovac	Symptomatic and Asymptomatic population	Healthcare workers	Prime and/or boost inoculation (NA)	19 January, 2021 to 25 March, 2021	P.1 （B.1.1.28）
Laith et al. 2021[23]	54338	A test negative case–control study	Qatar	NA	NA	BNT162b2 (NA)	mRNA vaccine	Pfizer-BioNTech	Unvaccinated Population	Persons In Qatar	Prime and boost inoculation (0, ≥14 days)	December 21, 2020. to March 31, 2021	B.1.1.7/B.1.351
Bernal et al. 2021[24]	12675	A test negative case control Study	India	NA	6200(48.9%)	BNT162b2(NA) /ChAdOx1(AZD1222,NA)	mRNA/Virus-vectored vaccine	Pfizer-BioNTech /AstraZeneca	Unvaccinated Population	Persons in India	Prime and boost inoculation (0, ≥21 days)	17 May 2021 to 16 May 2021	B.1.617.2/B.1.1.7
Tiraboschi et al. 2021[25]	6904	A cross-sectional study	Italy	NA	NA	BNT162b2 (NA)	mRNA vaccine	Pfizer-BioNTech	Symptomatic and Asymptomatic population	Healthcare workers in Brescia	Prime and boost inoculation (0, 21 days)	25th January 2021 to13th April 2021	B.1.1.7/B1.525
Hall et al.	23324	A prospective	England	46.1	3632(16	BNT162b2	mRNA	Pfizer-Bio	Unvaccinat	Staff (aged ≥18	Prime and	Dec 7,	B.1.1.7

2021[26]		cohort study		(36.0–54.1)	%)	(NA)	vaccine	NTech	ed Population	years) working in publicly-funded hospitals	boost inoculation (0, ＜19 days)	2020, to Feb 5, 2021.	
Jacobson et al. 2021[27]	189	A Cross-sectional study	USA	40.8 (11.2)	64 (33.9%)	BNT162b2 (NA)	mRNA vaccine	Pfizer-BioNTech	Infection with wild-type Or B.1.427/B.1.429	Healthcare personnel	Prime and boost inoculation (NA)	December 2020 to March 2021	B.1.427/B.1.429
Haas et al. 2021[28]	4714932	An observational study	Israel	NA(16 and older)	310788 (72·2%)	BNT162b2 (NA)	mRNA vaccine	Pfizer-BioNTech	Unvaccinated Population	Residents in Israel	Prime and boost inoculation (0, 21 days)	Jan 24 to April 3, 2021)	B.1.1.7
Emary et al. 2021[29]	520	A single-blind, multicentre, randomised phase 2/3 trial	UK	NA(18 -55)	213 (41%)	ChAdOx1(AZD1222,NA)	Virus-vectored vaccine	AstraZeneca	A meningococcal conjugate control (MenACWY) vaccine	Volunteers in UK	Prime and boost inoculation (NA)	Oct 1, 2020, and Jan 14, 2021	B.1.1.7
Bailly et al.	31	An	France	87.0(8.2)	11	BNT162b2(N	mRNA	Pfizer-Bio	Non-vaccin	Residents	Prime and	March	B.1.351

2021[30]		observational cohort study			(35.5%)	A)	vaccine	NTech	ated residents	in a nursing home	boost inoculation (NA)	8th to March 29th,	(501Y.V2)
Cavanaugh et al. 2021[31]	199	A case-control study	USA	NA(NA)	NA	BNT162b2(NA)	mRNA vaccine	Pfizer-BioNTech	Unvaccinated Population	Health care personnel And residents in a skilled nursing facilities	Prime and/or boost inoculation (NA)	NA	R.1
Kustin et al. 2021[32]	842	A case-control study	Israel	NA(＞16)	320(40.4%)	BNT162b2(NA)	mRNA vaccine	Pfizer-BioNTech	Unvaccinated Population	Individuals in Clalit health services in Israel	Prime and/or boost inoculation (NA)	December 20, 2020-mid March 2021	B.1.1.7/B.1.351
Magalis et al. 2021[33]	409	An observational cohort study	USA	34.0(＜35)	40 (10%)	BNT162b2(NA)	mRNA vaccine	Pfizer-BioNTech	NA	Population in Alachua County, Florida	2 standard doses (NA)	Between February and March 2021	B.1.1.7/B.1.377/B.1.429/B.1
Faria et al. 2021[34]	142	An observational	Brazil	NA(NA)	NA	CoronaVac (600 SU)	Inactivated vaccine	Sinovac Life	NA	Healthcare workers in São	Prime and boost	From 23 February	P1, B.1.1.7, and

		cohort study						Sciences		Paulo	inoculation (0, 2-4weeks)	,2020 through 28 March, 2021	B.1.351
Irfan et al. 2021[35]	2026	A multicenter, randomized, placebo-controlled trial	South Africa	30(18-<65)	115(57%)	ChAdOx1(AZD1222)(0.33 to 0.5 mL)	Virus-vectored vaccine	AstraZeneca	Placebo(0.9 % sodium chloride)	Population had no or well-controlled chronic medical conditions	2 standard doses (0, 21-35 days)	NA	B.1.351 (501Y.V2)
Grant et al.2021[36]	13728	A nationwide case-control study	France	NA(NA)	3940(31.2%)	ChAdOx1(AZD1222,NA) and BNT162b2(NA)	Virus-vectored Vaccine and mRNA vaccine	AstraZeneca/Pfizer-BioNTech	Unvaccinated Population	French population from Ipsos(a market research and public opinion specialist company)	Prime and/or boost inoculation (NA)	Between 23 May and 13 August 2021	B.1.617.2
Tang et al. 2021[37]	56561	A matched test-negative case-control study	Qatar	NA	35747(63.2%)	BNT162b2(NA), mRNA-1273 vaccine(NA)	mRNA vaccine	Pfizer-BioNTech / Moderna	Unvaccinated Population	Qatar' population	Prime and/or boost inoculation (NA)	From 21 December 2020 to 7 September 2021	B.1.617.2

Rovida et al.2021[38]	3616	An observational cohort study	Italy	NA	NA	BNT162b2(NA)	mRNA vaccine	Pfizer-BioNTech	Unvaccinated Healthcare workers	Healthcare workers of of San Matteo hospital, Pavia, Italy	2 standard doses (NA)	From January 18 to April 19 2021	B.1.1.7
Pouwels et al.2021[39]	3565037	An observational cohort study	UK	NA	NA	BNT162b2(NA),ChAdOx1 (AZD1222,NA),mRNA-1273 vaccine(NA)	mRNA Vaccine/Virus-vectored vaccine	Pfizer-BioNTech/AstraZeneca /Moderna	Unvaccinated Population in UK	Population in UK	Prime or boost inoculation (NA)	From 1 Dec 2020 to 2 August 2020	B.1.1.7/B.1.617.2
Trunfio et al.2021[40]	6800	An observational cohort study	Italy	NA	NA	BNT162b2(NA)	mRNA Vaccine	Pfizer-BioNTech	NA	Healthcare workers in the entire health district Città di Torino	Prime or boost inoculation (NA)	From 27 December 2020 until 1 April 2021	B.1.1.7
Heath et al.2021[41]	14039	A randomized, observer-blinded, placebo-controlled trial	UK	56 (18–84)	7238 (51.6%)	NVX-CoV2373(5 μg of recom Binant spike protein)	Recombinant subunit vaccine	Novavax	Saline placebo	Adults in the United Kingdom	Prime and boost inoculation (21 days apart)	From September 28 to November 28, 2020	B.1.1.7
Yi et al.2022[42]	58	An observational cohort study	Korean	NA	16 (27.6%)	BNT162b2(NA)	mRNA Vaccine	Pfizer-BioNTech	Unvaccinated Population	Population in Korean	Prime and boost inoculation	From August 3 to	B.1.617.2

									in Korean		(mean140days apart)	August 10 2021	
Chemaitelly et al.2021[43]	486138	A matched test-negative case-control study	Qatar	NA	474306(68.0%)	BNT162b2(NA)	mRNA Vaccine	Pfizer-BioNTech	Unvaccinated Population in Qatar	Population in Qatar	Prime or boost inoculation (NA)	from January 1 to September 5, 2021	B.1.617.2, B.1.351, B.1.617.2
Li et al.2021[44]	366	A test-negative case–control study	China	NA	63 (41.2%)	CoronaVac(NA)	inactivated vaccine	NA	Unvaccinated Population in Guanzhou, China	Population in Guanzhou China	Prime or boost inoculation (NA)	From February 2 through May 1, 2021	B.1.617.2
Clemens et al.2021[45]	9433	A matched multi-centre study	Brazil	NA	4268(45.2%)	ChAdOx1(NA)	Virus-vectored vaccine	AstraZeneca	Unvaccinated participants in Brazil	Population in Brazil	Prime and boost inoculation (NA)	Between June 23, 2020 and December 1, 2020	B.1.1.33, B.1.1.28, P.2, P.1, B.1.617.2
Thiruvengadam et al.2021[46]	5143	A test-negative, case-control study	India	NA	3430(66.7%)	ChAdOx1(NA)	Virus-vectored vaccine	AstraZeneca	Unvaccinated participants in India	Population in India	Prime or boost inoculation (NA)	Between April 1, 2021, and May 31, 2021	B.1.617.2

Tenforde et al.2021[47]	1831	A multicenter case-control study	US	NA	902(50.7%)	BNT162b2(NA), mRNA-1273 vaccine(NA)	mRNA vaccine	Pfizer-BioNTech / Moderna	Unvaccinated participants in US	Population in US	Prime or boost inoculation (NA)	Form March 11 to May 5, 2021	B.1.1.7，P.1，B.1.429, B.1.351, etc.
Williams et al.[48]	42	An observational cohort study	England	NA	14(33.3%)	ChAdOx1-S(NA)	Virus-vectored vaccine	AstraZeneca	Unvaccinated population in a care home, London, England	Vaccinated population in a care home, London, England	Prime or boost inoculation (NA)	Since summer 2020,	B.1.617.2
Lefèvre et al.2021[49]	378	An observational cohort study	France	NA	NA	BNT162b2(NA)	mRNA vaccine	Pfizer-BioNTech	NA	Residents in longterm care facilities in France	Prime and boost inoculation (NA)	Between Jan 15 and April 16, 2021	B.1.351
Desai et al.2021[50]	2136	A test-negative, case-control study	India	NA	NA	BBV152	inactivated vaccine	Bharat Biotech	Unvaccinated population in India	Population in India	Prime and boost inoculation (at least 14 days)	Between April 15 and May 15, 2021	B.1.617.2
Duerr et al.2021[51]	152	An observational case control study	US	NA	NA	BNT162b2/mRNA-1273/JNJ-78436735	mRNA vaccine	Pfizer-BioNTech/Moderna/Janssen	Unvaccinated population in New	Population in New York City	Prime and boost inoculation (NA)	Between February 1 and April 30,	B.1.1.7, B.1.526, P.1

									York City			2021,	
Bruxvoort et al.2021[52]	48918	A test negative case-control study.	UK	NA	NA	mRNA-1273	mRNA vaccine	Moderna	Test negative controls on age, sex, etc.	Adult Kaiser Permanente Southern California (KPSC)members	Prime and/or boost inoculation (≥14 days)	From 1 March 2021 to 27 July 2021	B.1.1.7, B.1.617.2, B.1.427, B.1.429, P.1, etc.
Hitchings et al.2021[53]	138673	A matched, test-negative case-control study	Brazil	NA	63493(45.8%)	ChAdOx1	Virus-vectored vaccine	AstraZeneca	Unvaccinated participants in Brazil	Older adults in São Paulo state, Brazil	Prime and/or boost inoculation (NA)	From Oct 1, 2020 to July 2, 2021	P.1
Dunkle et al.2021[54]	29949	A phase 3, randomized, observer-blinded, placebo-controlled trial	the United States and Mexico	47.0 (18–95)	13181 (51.8%)	NVX-CoV2373	Recombinant subunit vaccine	Novavax	Adults receive two doses of placebo	Adults receive two doses of NVX-CoV2373	Prime and boost inoculation (≥21 days)	Between December 27, 2020, and February 18, 2021	B.1.1.7, B.1.351, B.1.526, P.1, B.1.429, B.1.617.1, P.2 , B.1.2
Ranzani et al.2021[55]	74363	A test negative case-control study	Brazil	NA	34472(46.4%)	CoronaVac	Inactivated vaccine	Sinovac	Adults aged ≥70 years who were residents of São Paulo state	Symptomatic covid-19 in the elderly population of São Paulo state	Prime and/or boost inoculation (NA)	From 17 January 2021 to 29 April 2021	P.1

Dickerman et al.2022[56]	439684	A matched, case-control study	US	69 (60–74)	407452 (92.7%)	BNT162b2/mRNA-1273	mRNA vaccine	Pfizer-BioNTech / Moderna	U.S. Veterans	U.S. Veterans	Prime inoculation (NA)	Between January 4 and May 14, 2021	B.1.1.7
Vignier et al.2021[57]	44	An observational cohort study	French Guiana	NA	42(95.5%)	BNT162b2	mRNA vaccine	Pfizer-BioNTech	Invaccinated gold miners	Vaccinated gold miners	Prime and/or boost inoculation (NA)	Between June 2, 2021 And June 15, 2021	P.1 (Gamma)
Abu-Raddad et al.2021[58]	295166	A test-negative case–control study	Qatar	NA	NA	BNT162b2	mRNA vaccine	Pfizer-BioNTech	Invaccinated population in Qatar	Vaccinated population in Qatar	Prime and/or boost inoculation (14 days)	From 1 February to 31 March 2021	B.1.1.7，B.1.351
Reis et al.2022[59]	188708	A matched, case-control study	Israel	NA	92450(49%)	BNT162b2	mRNA vaccine	Pfizer-BioNTech	Invaccinated adolescents	Vaccinated adolescents	Prime and/or boost inoculation (NA)	Between June 8 and September 14, 2021	B.1.617.2
Olson et al.2021[60]	464	A test-negative, case-control	US	15(14-17)	253(55.7%)	BNT162b2	mRNA vaccine	Pfizer-BioNTech	Invaccinated Persons	Vaccinated Persons Aged	Prime and boost	During June	B.1.617.2

		study							Aged 12-18 Years	12-18 Years	inoculation (NA)	1–September 30, 2021	
Collie et al.2022[61]	78173	A test-negative, case-control study	South African	NA	NA	BNT162b2	mRNA vaccine		Invaccinated population	Vaccinated populatio	Prime and boost inoculation (NA)		B.1.1.529 (omicron)
Eyre et al.2022[62]	108498	A retrospective observational cohort study	England	NA	NA	ChAdOx1/BNT162b2	Virus-Vectored/mRNA vaccine	AstraZeneca/Pfizer-BioNTech	Invaccinate Population in England	VaccinatePopulation in England	Prime OR boost inoculation (NA)	NA	B.1.1.7 (Alpha)
Mor et al.2022[63]	3050	A retrospective observational cohort study	Israel	NA	NA	BNT162b2	mRNA vaccine	Pfizer-BioNTech	Invaccinate Population in Israel	accinatePopulation in Israel	Boost inoculation (NA)	During winter 2020–2021	B.1.1.7(alpha)，B.1.351(Beta)
