# Supplementary material for: Real-Word Effectiveness of Global COVID-19 Vaccines Against SARS-CoV-2 Variants: A Systematic Review and Meta-Analysis
Source: Front Med (Lausanne). 2022 May 19;9:820544. doi: 10.3389/fmed.2022.820544 (PMC9160927; doi:10.3389/fmed.2022.820544)
Supplement: Supplementary Table 2 — Results of the Egger test, the heterogeneity test, and the meta-analysis. [file Table_2.pdf]

Supplementary table 2: Results of the Egger test, the heterogeneity test and the meta-analysis

Groups	No.of studies	Egger test		Heterogeneity test			Results of the meta-analyses			
		t	p	I^2	τ^2	p	Model	Single-arm analysis*	Two-arm analysis#	
									OR	VE
Incidence of variants post first vaccine	6	1.86	0.12	100%	0.0351	0	Random effect model	0.07[0.01;0.15]		
Incidence of variants post second vaccine	21	4.32	0.0003	100%	0.0106	0	Random effect model	0.03[0.02;0.04]		
Incidence of variants post first mRNA vaccine	4	2.17	0.1618	100%	0.0478	0	Random effect model	0.07[0.00; 0.21]		
Incidence of variants post second mRNA vaccine	10	2.25	0.0548	100%	0.0062	0	Random effect model	0.06[0.04; 0.09]		
Incidence of variants post second viral vector vaccine	4	0.39	0.7358	93%	0.0011	< 0.01	Random effect model	0.02[0.01; 0.02]		
Incidence of variants post second protein subunit vaccine	2	-	-	99%	0.0044	< 0.01	Random effect model	0.03[0.00; 0.03]		
Incidence of variants post second inactivated vaccine	2	-	-	96%	0.0196	< 0.01	Random effect model	0.37[0.19; 0.57]		
Incidence of B.1.1.7(Alpha) variant post first vaccine	11	2.83	0.0142	100%	0.0089	0	Random effect model	0.07[0.05; 0.10]		
Incidence of B.1.1.7(Alpha) variant post second vaccine	22	3.32	0.0031	100%	0.0032	0	Random effect model	0.04[0.03; 0.05]		
Incidence of B.1.1.7(Alpha) variant post first mRNA vaccine	8	3.29	0.0072	100%	2.1904	0	Random effect model	0.16[0.15; 0.16]		
Incidence of B.1.1.7(Alpha) variant post second mRNA vaccine	11	1.98	0.0794	100%	0.0127	0	Random effect model	0.09[0.06; 0.14]		
Incidence of B.1.1.7(Alpha) variant post first viral vector vaccine	3	1.58	0.3584	100%	0.0009	0	Random effect model	0.10[0.07; 0.14]		
Incidence of B.1.1.7(Alpha) variant post second viral vector vaccine	4	2.10	0.1701	98%	0.0001	0	Random effect model	0.00[0.00; 0.01]		
Incidence of B.1.1.7(Alpha) variant post second protein subunit vaccine	2	-	-	86%	0.0001	< 0.01	Random effect model	0.00[0.00; 0.00]		
Incidence of B.1.351 (Beta) variant post first vaccine	4	2.69	0.1147	100%	0.0986	0	Random effect model	0.35[0.04; 0.66]		
Incidence of B.1.351 (Beta) variant post second vaccine	12	3.40	0.0067	100%	0.0575	0	Random effect model	0.09[0.03; 0.19]		
Incidence of B.1.351 (Beta) variant post first mRNA vaccine	4	-1.21	0.3506	100%	0.0415	< 0.01	Random effect model	0.30[0.14; 0.50]		
Incidence of B.1.351 (Beta) variant post second mRNA vaccine	6	-32.80	<0.000	99%	0.0398	< 0.01	Random effect model	0.10[0.03; 0.22]		
Incidence of B.1.351 (Beta) variant post second viral vector vaccine	2	-	-	0	0	0.35	Fixed effect model	0.02[0.02; 0.03]		

Incidence of B.1.351 (Beta) variant post second protein subunit vaccine	2	-	-	98%	0.0041	< 0.01	Random effect model	0.00[0.00; 0.02]		
Incidence of P.1 (Gamma) variant post first vaccine	10	0.63	0.5463	100%	0.1408	0	Random effect model	0.14[0.02; 0.34]		
Incidence of P.1 (Gamma) variant post second vaccine	4	-1.64	0.1995	85%	2.7929	< 0.01	Random effect model	0.09[0.06; 0.16]		
Incidence of P.1 (Gamma) variant post first mRNA vaccine	3	4.11	0.1518	94%	0.0338	< 0.01	Random effect model	0.09[0.00; 0.26]		
Incidence of P.1 (Gamma) variant post second mRNA vaccine	4	1.81	0.2126	95%	0.0231	< 0.01	Random effect model	0.06[0.01; 0.16]		
Incidence of P.1 (Gamma) variant post second inactivated vaccine	3	-0.38	0.7672	99%	0.0079	< 0.01	Random effect model	0.36[0.26; 0.46]		
Incidence of P.1 (Gamma) variant post second viral vector vaccine	2	-	-	100%	7.2064	< 0.01	Random effect model	0.05[0.00; 0.67]		
Incidence of B.1.617.2(Delta) variant post first vaccine	12	3.51	0.0032	100%	0.0132	0	Random effect model	0.14[0.11; 0.18]		
Incidence of B.1.617.2(Delta) variant post second vaccine	11	2.59	0.0236	100%	0.0095	0	Random effect model	0.08[0.05; 0.11]		
Incidence of B.1.617.2(Delta) variant post first mRNA vaccine	9	2.29	0.0619	100%	0.0356	0	Random effect model	0.09[0.03; 0.18]		
Incidence of B.1.617.2(Delta) variant post second mRNA vaccine	8	2.16	0.0629	100%	0.0154	0	Random effect model	0.09[0.05; 0.14]		
Incidence of B.1.617.2(Delta) variant post first viral vector vaccine	3	84.31	0.0076	99%	0.0089	< 0.01	Random effect model	0.06[0.02; 0.14]		
Incidence of B.1.617.2(Delta) variant post second viral vector vaccine	3	0.95	0.5147	100%	0.0126	0	Random effect model	0.03[0.00; 0.09]		
Incidence of B.1.427(Epsilon) variant post first vaccine	3	0.56	0.6762	97%	3.5517	< 0.01	Random effect model	0.00[0.00; 0.04]		
Incidence of B.1.427(Epsilon) variant post second vaccine	5	0.34	0.7577	83%	5.8885	< 0.01	Random effect model	0.00[0.00; 0.01]		
Incidence of B.1.427(Epsilon) variant post first mRNA vaccine	3	0.56	0.6762	97%	3.5517	< 0.01	Random effect model	0.00[0.00; 0.04]		
Incidence of B.1.427(Epsilon) variant post second mRNA vaccine	2	-	-	0%	1.5772	1.00	Fixed effect model	0.00[0.00; 0.04]		
Incidence of P.2 (Zeta) variant post second vaccine	2	-	-	0%	21.5506	1.00	Fixed effect model	0.00[0.00; 0.22]		
Incidence of B.1.526 (Iota) variant post second vaccine	2	-	-	99%	19.6635	< 0.01	Random effect model	0.01[0.00; 0.80]		
Incidence of B.1.526 (Iota) variant post second mRNA vaccine	2	-	-	96%	2.7961	< 0.01	Random effect model	0.12[0.01; 0.59]		
Efficacy of vaccines against variants post first dose	5	-0.63	0.5617	98%	0.1213	< 0.01	Random effect model		0.60[0.58;0.62]	0.40[0.38,0.42]
Efficacy of vaccines against variants post second dose	14	3.26	0.0062	100%	3.7957	0	Random effect model		0.04[0.02;0.07]	0.96[0.93;0.98]
Efficacy of mRNA vaccine against variants post first dose	3	-0.81	0.5682	95%	0.0583	< 0.01	Random effect model		0.65[0.49; 0.87]	0.35[0.13;0.51]
Efficacy of mRNA vaccine against variants post second dose	7	2.25	0.0548	100%	4.3658	0	Random effect model		0.15[0.03; 0.72]	0.85[0.28;0.97]
Efficacy of viral vector vaccine against variants post second dose	4	0.02	0.9862	76%	0.0984	< 0.01	Random effect model		0.34[0.23; 0.49]	0.66[0.51;0.77]
Efficacy of vaccines against B.1.1.7(Alpha) variant post first dose	9	1.80	0.1028	100%	1.1959	0	Random effect model		0.34[0.18; 0.64]	0.66[0.36;0.82]

Efficacy of vaccines against B.1.1.7(Alpha) variant post second dose	18	4.06	0.0007	100%	2.8773	0	Random effect model	0.10[0.05; 0.21]	0.90[0.79;0.95]
Efficacy of mRNA vaccine against B.1.1.7(Alpha) variant post first dose	8	1.76	0.1283	100%	2.1904	0	Random effect model	0.36[0.13; 1.00]	0.64[0.00;0.87]
Efficacy of mRNA vaccine against B.1.1.7(Alpha) variant post second dose	13	3.48	0.0052	100%	2.5342	0	Random effect model	0.11[0.05; 0.26]	0.89[0.74;0.95]
Efficacy of subunit vaccine against B.1.1.7(Alpha) variant post second dose	2	-	-	7%	0.0160	0.30	Fixed effect model	0.11[0.06; 0.20]	0.89[0.80;0.94]
Efficacy of viral vector vaccine against B.1.1.7(Alpha) variant post second dose	4	1.42	0.2916	100%	11.6941	< 0.01	Random effect model	0.06[0.00; 1.70]	0.94[0.30;1.00]
Efficacy of vaccines against B.1.351(Beta) variant post first dose	4	1.52	0.2686	0%	0	0.47	Fixed effect model	0.84[0.80; 0.89]	0.16[0.11;0.20]
Efficacy of vaccines against B.1.351(Beta) variant post second dose	9	0.97	0.3640	97%	0.7131	< 0.01	Random effect model	0.58[0.30; 1.12]	0.42[0.00;0.70]
Efficacy of mRNA vaccine against B.1.351(Beta) variant post first dose	4	1.52	0.2686	0%	0	0.47	Fixed effect model	0.84[0.80; 0.89]	0.16[0.11;0.20]
Efficacy of mRNA vaccine against B.1.351(Beta) variant post second dose	6	1.04	0.3577	98%	0.7275	< 0.01	Random effect model	0.60[0.28; 1.28]	0.40[0.00;0.72]
Efficacy of vaccines against P.1 (Gamma) variant post first dose	5	-0.48	0.6639	99%	0.1037	< 0.01	Random effect model	0.65[0.44; 0.95]	0.35[0.05;0.56]
Efficacy of vaccines against P.1 (Gamma) variant post second dose	8	-1.05	0.3345	86%	0.0486	< 0.01	Random effect model	0.39[0.30; 0.50]	0.61[0.50;0.70]
Efficacy of mRNA vaccine against P.1 (Gamma) variant post first dose	3	3.09	0.1996	23%	0.1379	0.27	Fixed effect model	0.43[0.19; 0.95]	0.57[0.05;0.81]
Efficacy of mRNA vaccine against P.1 (Gamma) variant post second dose	4	2.18	0.1605	85%	2.7929	< 0.01	Random effect model	0.32[0.05; 1.99]	0.68[0.00;0.95]
Efficacy of viral vector vaccine against P.1 (Gamma) variant post second dose	2	-	-	74%	0.1226	0.05	Random effect model	0.43[0.25; 0.75]	0.57[0.25;0.75]
Efficacy of vaccines against B.1.617.2(Delta) variant post first dose	12	0.18	0.8630	98%	0.3453	< 0.01	Random effect model	0.62[0.45; 0.85]	0.38[0.15;0.55]
Efficacy of vaccines against B.1.617.2(Delta) variant post second dose	12	-1.02	0.3233	98%	0.3147	< 0.01	Random effect model	0.32[0.24; 0.43]	0.68[0.57;0.76]
Efficacy of mRNA vaccine against B.1.617.2(Delta) variant post second dose	8	-2.17	0.0617	99%	0.3785	< 0.01	Random effect model	0.26[0.18; 0.38]	0.74[0.62;0.82]
Efficacy of viral vector vaccine against B.1.617.2(Delta) variant post first dose	3	-0.14	0.9121	89%	0.0427	< 0.01	Random effect model	0.50[0.39; 0.65]	0.50[0.35;0.61]
Efficacy of viral vector vaccine against B.1.617.2(Delta) variant post second dose	3	1.14	0.4590	88%	0.1828	< 0.01	Random effect model	0.38[0.21; 0.69]	0.62[0.31;0.79]
Efficacy of vaccines against B.1.427(Epsilon) variant post first dose	2	-	-	0%	0	0.73	Fixed effect model	0.22[0.10; 0.46]	0.78[0.54;0.90]
Efficacy of vaccines against B.1.427(Epsilon) variant post second dose	3	-1.82	0.3195	0%	0	0.84	Fixed effect model	0.05[0.02; 0.13]	0.95[0.87;0.98]

Efficacy of mRNA vaccine against B.1.427(Epsilon) variant post first dose	2	-	-	0%	0	0.73	Fixed effect model	0.22[0.10; 0.46]	0.78[0.54;0.90]
Efficacy of mRNA vaccine against B.1.427(Epsilon) variant post second dose	2	-	-	0%	0	0.58	Fixed effect model	0.05[0.02; 0.14]	0.95[0.86;0.98]
Efficacy of vaccines against P.2(Zeta) variant post second dose	2	-	-	0%	0	0.67	Fixed effect model	0.31[0.22; 0.45]	0.69[0.55;0.78]
Efficacy of vaccines against B.1.526 (Iota) variant post second dose	3	-0.17	0.8913	90%	2.6893	< 0.01	Random effect model	0.29[0.04; 2.11]	0.71[0.00;0.96]
Efficacy of mRNA vaccines against B.1.526 (Iota) variant post second dose	2	-	-	94%	3.7222	< 0.01	Random effect model	0.38[0.02; 5.93]	0.62[0.00;0.98]

OR: Odds ratio, VE: Vaccine effectiveness
